# An unexpected role of Nogo-A as regulator of tooth enamel formation

**DOI:** 10.1038/s41368-024-00323-x

**Published:** 2024-10-20

**Authors:** Pierfrancesco Pagella, Chai Foong Lai, Laurence Pirenne, Claudio Cantù, Martin E. Schwab, Thimios A. Mitsiadis

**Affiliations:** 1 Orofacial Development and Regeneration, Institute of Oral Biology, University of Zürich, Zürich, Switzerland; 2 Laboratory of Molecular Materials, Division of Biophysics and Bioengineering, Department of Physics, Chemistry, and Biology (IFM), Linköping University, Linköping, Sweden; 3 Wallenberg Centre for Molecular Medicine, Linköping University, Linköping, Sweden; 4 Department of Biomedical and Clinical Sciences, Division of Molecular Medicine and Virology; Faculty of Medicine and Health Sciences, Linköping University, Linköping, Sweden; 5 Institute for Regenerative Medicine, University of Zürich, Zürich, Switzerland

**Keywords:** Mutagenesis, Dental diseases

## Abstract

Neurite outgrowth inhibitor A (Nogo-A) is a major player in neural development and regeneration and the target of clinical trials aiming at promoting the regeneration of the central nervous system upon traumatic and ischemic injury. In this work, we investigated the functions of Nogo-A during tooth development to determine its role in dental physiology and pathology. Using immunohistochemistry and in situ hybridization techniques, we showed that Nogo-A is highly expressed in the developing mouse teeth and, most specifically, in the ameloblasts that are responsible for the formation of enamel. Using both *Nogo-A* knockout and *K14-Cre;Nogo-A fl/fl* transgenic mice, we showed that Nogo-A deletion in the dental epithelium leads to the formation of defective enamel. This phenotype is associated with overexpression of a set of specific genes involved in ameloblast differentiation and enamel matrix production, such as *amelogenin*, *ameloblastin* and *enamelin*. By characterising the interactome of Nogo-A in the dental epithelium of wild-type and mutant animals, we found that Nogo-A directly interacts with molecules important for regulating gene expression, and its deletion disturbs their cellular localisation. Furthermore, we demonstrated that inhibition of the intracellular, but not cell-surface, Nogo-A is responsible for gene expression modulation in ameloblasts. Taken together, these results reveal an unexpected function for Nogo-A in tooth enamel formation by regulating gene expression and cytodifferentiation events.

## Introduction

Nogo proteins are myelin-derived inhibitors of neurite outgrowth encoded by the *Reticulon-4 (RTN4)* gene.^1^ Alternative splicing of *RTN4* generates the Nogo-A (200 kD), Nogo-B (55 kD) and Nogo-C (25 kD) membrane-binding isoforms that share a conserved C-terminal domain but contain different N-terminal domains. Nogo-A, the most studied of the three isoforms, contains a specific N-terminal extracellular domain (named Nogo-A-Δ20).^2^ The C‐terminal part includes the Nogo-66 amino acid loop and an endoplasmic reticulum (ER) retention motif.^3^ The major portion of Nogo-A is located intracellularly at the endoplasmic reticulum (ER) membrane.^4^ where it can act as modulator of the ER curvature.^5^ A small portion of Nogo-A is also present on the cell surface, where it exerts signalling functions by interacting with multi-subunit receptor complexes, including NgR1 (Nogo Receptor1), PirB (Paired immunoglobulin-like receptor B),^6^ S1PR2 (sphingosine1-phosphate receptors2),^7^ and heparan sulphate proteoglycans (HSPGs).^8^ NgR1 usually forms complexes with other transmembrane receptors including LINGO-1 (immunoglobulin-like domain-containing protein-1), p75NTR (p75 neurotrophin receptor) and TROY (TNF receptor superfamily member 19).^9–11^ Nogo-66 binding to the various NgR1 complexes activates RhoA and ROCK (Rho-Associated Kinase) signalling that promotes actin disassembly and microtubule depolymerization.^12–14^

Nogo-A is a major player in the control of neural development and regeneration.^4,15–17^ While central nervous system (CNS) injuries lead to Nogo-A upregulation at the wounded sites,^18^ which inhibits axonal regeneration,^4,15^ the process of neuronal regeneration can be re-established upon blockage of Nogo-A function by either genetic deletion or antibody/peptide administration.^4,19,20^ Besides its neuronal functions, Nogo-A is a negative regulator of angiogenesis, vascular remodelling, and vessel network architecture in the CNS.^21,22^ Nogo-A thus represents a promising therapeutic target both for CNS regeneration and for angiogenesis-linked brain pathologies.^23^ Despite the high clinical relevance of Nogo-A, very little is known about its functions outside the CNS. Few recent studies have indicated an additional role for Nogo-A in inflammatory processes and diseases due to its activation in macrophages.^24^

We recently showed that Nogo-A modulates trigeminal neuron differentiation, affecting both the number and patterning of neurons in the dental pulp of rodent teeth.^25^ Furthermore, we observed that Nogo-A is expressed in adult human dental pulp and that its inhibition modulates the differentiation of human dental pulp stem cells towards the osteogenic, adipogenic, and neurogenic lineages in vitro.^26^ Based on these considerations, we set out to investigate the expression and functions of Nogo-A during tooth development in transgenic mouse models. Teeth develop through a complex set of interactions between the dental epithelium and the neural crest-derived mesenchyme.^27^ Differentiation of mesenchymal cells gives rise to the dental pulp and the dentin-producing odontoblasts. Cells of the dental epithelium differentiate into functional ameloblasts that produce and secrete the extracellular matrix of enamel, which displays unique mechanical properties and constitutes the hardest tissue of the vertebrate body.^28^ Enamel formation relies on a series of coordinated and concerted processes that include the secretion of enamel matrix proteins such as amelogenin, ameloblastin, and enamelin, the mineralization of the enamel matrix, and the degradation of the enamel matrix proteins by specific proteases such as Klk4 and Mmp20.^28^

We showed here that Nogo-A is also expressed in the dental epithelium and that its deletion in this tissue leads to enamel defects. We further demonstrate that these effects are exclusively mediated by intracellular Nogo-A via modulation of the expression of genes fundamental for amelogenesis. Taken together, the present findings reveal a yet unknown function for Nogo-A as a modulator of tooth enamel formation.

## Results

### Nogo-A expression during tooth development

**Fig. 1:**
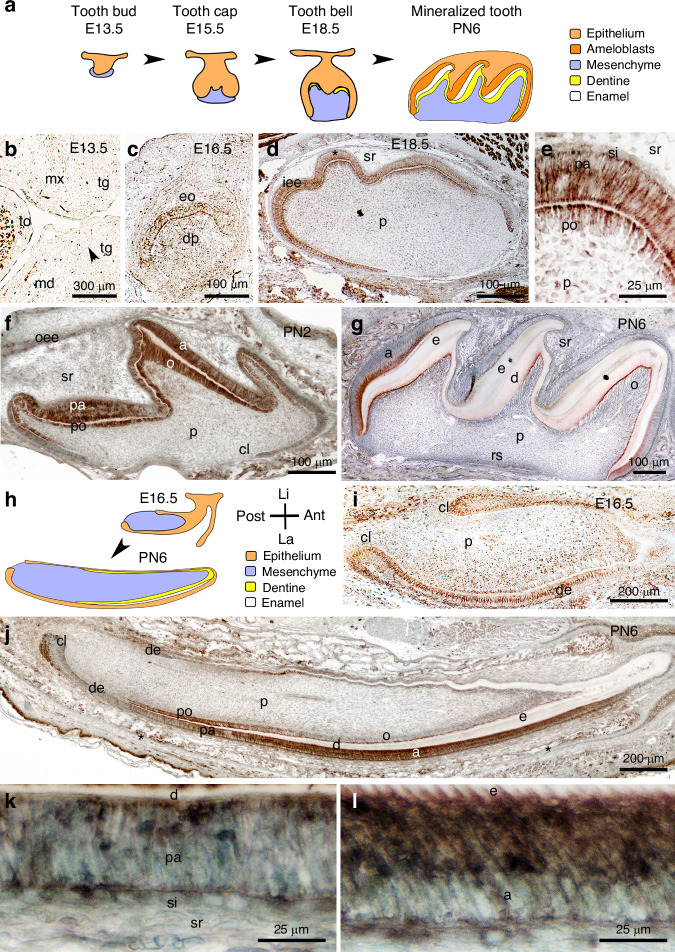
Nogo-A expression during tooth development. **a** Schematic representation of molar development. Immunohistochemistry against Nogo-A (brown colour) during the first molar development. Nogo-A localisation at embryonic days 13.5 (**b**), 16.5 (**c**), and 18.5 (**d**, **e**), and postnatal days 2 (**f**) and 6 (**g**). Asterisk in (**d**) indicates the magnified area showing Nogo-A distribution in preameloblasts and preodontoblasts (**e**). **h** Schematic representation of embryonic (E16.5) and postnatal (PN6) mouse incisors. Immunohistochemistry showing Nogo-A distribution in the lower incisor at embryonic day 16.5 (**I**) and postnatal day 6 (**j**–**l**). Asterisks in (**j**) indicate the areas of higher magnifications showing Nogo-A distribution in preameloblasts (**k**) and ameloblasts (**l**). Abbreviations: a ameloblasts, Ant anterior part, cl cervical loop, d dentine, de dental epithelium, dm dental mesenchyme, dp dental papilla, E embryonic day, e enamel, eo enamel organ, iee inner enamel epithelium, La labial part, Li lingual part, md mandibular process, mx maxillary process, o odontoblasts, oee outer enamel epithelium, p dental pulp, pa preameloblast, PN postnatal day, po preodontoblasts, Post posterior part, rs root sheath, si stratum intermedium, sr stellate reticulum, tg tooth germ, to tongue. Immunostainings for Nogo-A have been performed on >3 samples for each developmental stage

We observed that Nogo-A is expressed in several developing orofacial organs, including teeth (Fig. 1). Expression of Nogo-A was low in molars at E13.5 (cap stage, Fig. 1a, b), while at this stage it was strong in the trigeminal axons approaching the tooth germ (Fig. 1b). Nogo-A expression was clearly detectable, both at the protein (Fig. 1c) and mRNA level (Supplementary Fig. S1b), in the molar epithelium and mesenchyme from E16.5 on, coinciding with the onset of cytodifferentiation.^27,29^ At E18.5, Nogo-A was highly expressed both in pre-odontoblasts and pre-ameloblasts (Protein: Fig. 1d, e; mRNA: Supplementary Fig. S1c). The strong expression of Nogo-A persisted during early postnatal stages in differentiating pre-odontoblast and pre-ameloblasts, and in differentiated odontoblasts and ameloblasts (Fig. 1f). Lower levels of Nogo-A were detected in other cells of the dental epithelium, while little to no expression was detected in the developing dental pulp (Fig. 1f). At postnatal day 6, Nogo-A was expressed at high levels in ameloblasts, while its expression disappeared in reduced ameloblasts (Fig. 1g). A similar expression pattern was observed in the incisors (Fig. 1h–l). At E16.5, Nogo-A was expressed throughout the dental epithelium, as well as within differentiating pre-odontoblasts (Protein: Fig. 1i; mRNA: Supplementary Fig. S1d–f). After birth, Nogo-A expression showed distinctive patterns in the dental epithelium and the mesenchyme. Within the dental epithelium, Nogo-A was expressed at high levels in the labial cervical loop, in pre-ameloblasts, and in ameloblasts, while it was not expressed in transit amplifying cells (Fig. 1j–l). Within the odontoblastic lineage, Nogo-A was strongly expressed in pre-odontoblasts, while it was downregulated in differentiated odontoblasts (Fig. 1j). We also assessed the expression of known receptors and coreceptors of Nogo-A (S1PR2, NgR1, P75/NGFR) in the dental epithelium of postnatal mouse incisors. S1PR2 labelling was restricted to ameloblasts (Supplementary Fig. S2), while NgR1 staining was absent from dental epithelium (Supplementary Fig. S2). P75/NGFR staining was observed in pre-ameloblasts, but not in functional ameloblasts (Supplementary Fig. S2).

### Nogo-A deletion leads to the formation of defective enamel

**Fig. 2:**
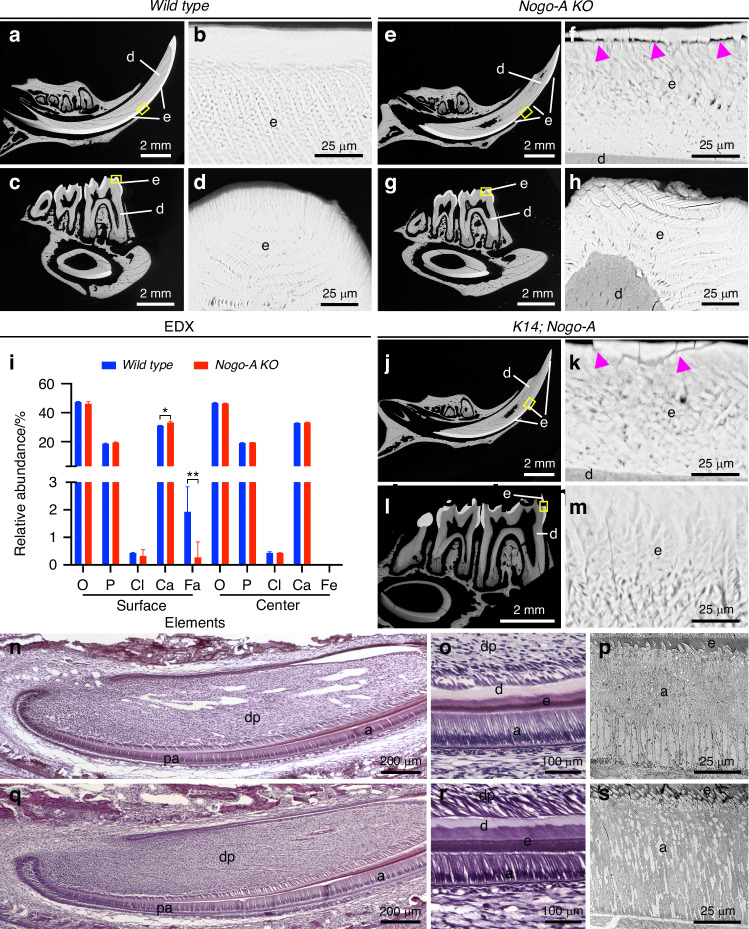
Nogo-A deletion leads to the formation of defective enamel. **a–h** Scanning electron microscopy (SEM) analysis of enamel structure in teeth of wild-type **a**–**d** and Nogo-A KO (**e**–**h**) mice. The yellow rectangles in (**a**), (**c**), (**e**), and (**g**) show the areas of higher magnifications in (**b**), (**d**), (**f**), and (**h**), respectively. Purple arrowheads in (**f**) highlight cracks in the enamel. **i** Energy-dispersive X-ray spectroscopy (EDX) analysis of the composition of enamel of wild-type and Nogo-A KO mice. **j–m** SEM analysis of enamel structure in teeth of K14:cre;Nogo-A KO mice. The yellow rectangles in (**j**) and (**l**) show the areas of higher magnifications in (**k**) and (**m**), respectively. Purple arrowheads in (**k**) highlight cracks in the enamel. **n, o** Hematoxylin-eosin staining of lower incisors of newborn wild-type mice. **p** Transmission electron microscopy (TEM) analysis of ameloblasts from lower incisors of wild-type adult mice. **q, r** Hematoxylin-eosin staining of lower incisors of new-born Nogo-A KO mice. **s** TEM analysis of ameloblasts from lower incisors of Nogo-A KO adult mice. Abbreviations: a ameloblasts, d dentine, dp dental pulp, e enamel, pa preameloblasts. Scale bars: **a**, **b**, **e**, **g**, **j**, **l**: 2 mm; **b**, **d**, **i**, **h**, **k**, **m**, **p**, s: 25 μm; **n**, **q**: 200 μm; **o**, **r**: 100 μm

The expression pattern is suggestive of a role for Nogo-A in tooth development. To study the effects of Nogo-A loss on tooth formation, we exploited a mouse mutant strain carrying a genetic deletion of the Nogo-A-specific exon 3, which leads to a complete and specific loss of Nogo-A (*Nogo-A KO*).^30^ Teeth in the *Nogo-A KO* mice were present, they appeared macroscopically normal and were functional under laboratory conditions. Backscatter scanning electron microscopy (SEM), however, showed major changes in enamel structure of non-decalcified molars and incisors of adult (2 months old) *Nogo-A KO* mice. Wild type teeth showed a highly organized and compact enamel structure, characterized by a highly spatially ordered disposition of rod and interrod crystals (Fig. 2a–d). On the contrary, teeth from all *Nogo-A KO* animals analysed (*n* = 6) showed a significant alteration in the fine structure of enamel. In the lower incisors, the enamel structure was compromised from the point of eruption to the tip of the incisor (Fig. 2e). In particular, rod crystals had highly variable orientation and dimensions (Fig. 2f). Similarly, the rods of molars enamel were not well organized in *Nogo-A KO* mice when compared to the those of the wild type mice (Fig. 2g, h). The mechanical properties of enamel depend both on its structure and chemical composition, and therefore we analysed the mineral composition of the enamel of wild type and *Nogo-A KO* lower incisors using energy-dispersive X-ray spectroscopy (EDX). EDX of wild type enamel showed iron enrichment at the surface, which accounted for approximately 1.5% of total enamel mass in erupting wild type incisors (Fig. 2i). By contrast, iron content was significantly reduced in the surface of the enamel of erupting incisors from *Nogo-A KO* mice. The relative abundance of all other elements was not significantly affected in the enamel of the mutant incisors, apart from calcium that was slightly increased ( +10%; Fig. 2i).

### Deletion of Nogo-A in the dental epithelium causes enamel defects

The phenotype described above was caused by the constitutive deletion of Nogo-A and did not allow us to discriminate which cell population required Nogo-A to guarantee proper enamel formation. Nogo-A is indeed expressed in several tissues involved in orofacial development and homeostasis, including nerve fibres and blood vessels.^31^ To test whether the enamel defects observed in *Nogo-A KO* mice could be associated with a function of Nogo-A in the dental epithelium, we analysed the teeth of *K14-Cre; Nogo-A fl/fl (K14;Nogo-A*) mice, where the K14-cre drivers leads to the selective deletion of Nogo-A in the dental epithelium^32^ (*n* = 6). SEM analysis showed that the deletion of Nogo-A in the dental epithelium led to enamel defects comparable to those observed in the systemic *Nogo-A KO* mice (Fig. 2j–m), thus indicating that Nogo-A possesses epithelium-specific roles.

We further characterized dental epithelium of Nogo-A mutant teeth with obvious enamel defects using histological analysis and transmission electron microscopy. Unlike the homogeneous palisade of tightly adherent, columnar, polarized ameloblasts in the wild-type dental epithelium (Fig. 2n–p), Nogo-A KO ameloblasts showed varying degrees of, impaired cell-cell adhesion (Fig. 2q–s). In the most severe cases, large spaces were observed within Nogo-A KO ameloblasts (Fig. 2s).

### Nogo-A is localized in ameloblasts endoplasmic reticulum and cytoplasm

**Fig. 3:**
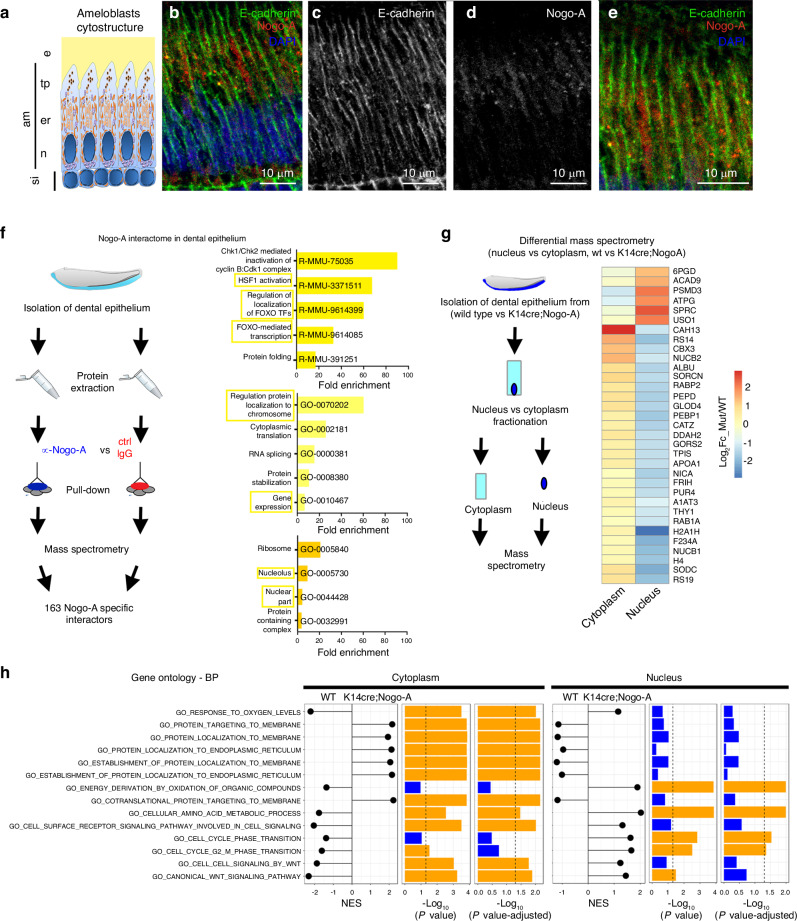
Nogo-A interacts with proteins involved in gene regulation, and Nogo-A deletion affects protein nuclear/cytoplasmic localisation. **a–e** Immunofluorescent microscopy analysis of Nogo-A subcellular localization in ameloblasts. **a** schematic representation of ameloblasts cytostructure. **b** Immunofluorescent staining showing Nogo-A (red colour) intracellular localisation in ameloblasts. Green colour: E-cadherin (marker of ameloblasts plasma membrane); blue colour: DAPI. **c, d** single-channel images of (**b**). **e**: higher magnification showing the mostly intracellular localization of Nogo-A within ameloblasts. Abbreviations: am ameloblasts, e enamel, er endoplasmic reticulum/Golgi apparatus, n nucleus, si stratum intermedium, tp Tomes’ processes. Scale bars: 10 μm **f** Experimental plan for the characterization of Nogo-A interactome in the dental epithelium and gene ontology analysis of Nogo-A specific interactors. Top histogram: reactome pathways; middle histogram: biological processes; lower histogram: cellular components. **g** Experimental plan for the study of nuclear/cytoplasmic protein localisation in the dental epithelium of *wild type* and *K14; Nogo-A* mutant incisors, and heatmap showing proteins whose relative nuclear/cytoplasmic localisation is affected in the epithelium of *K14; Nogo-A* mutant incisors. *P* < 0.05. **h** Gene Ontology (GO) analysis of proteins whose relative nuclear/cytoplasmic abundance is affected in the epithelium of *K14; Nogo-A* mutant incisors. NES indicates the Normalized Enrichment Score of the named GO term in the cytoplasm (“cytoplasm” column) or nucleus (“nucleus” column) in *wild type* or *K14; Nogo-A* mutant incisors. Coloured bars indicate -log_10_(*P* value) and -log_10_ (*P* value-adjusted)

The effects of Nogo-A deletion on enamel formation and ameloblasts adhesion led us to investigate in depth the function of Nogo-A in the dental epithelium. To this aim, we first analysed in deeper detail the expression of Nogo-A. We thus performed double immunostaining against Nogo-A and E-Cadherin, marker of the ameloblasts membrane.^33^ Nogo-A was nearly undetectable on the plasma membrane, while it was abundantly expressed and localized within ameloblasts cytoplasm (Fig. 3a–e). Closer inspection showed that Nogo-A was highly concentrated adjacent to the apical side of the ameloblast nuclei (Fig. 3b–e), where the endoplasmic reticulum (ER) and the Golgi apparatus are located (Fig. 3a). By contrast, Nogo-A staining was nearly absent from the apical and basal ends of ameloblasts (Fig. 3b–e). As Nogo-A is known to localize predominantly at the ER,^34^ this data suggests that Nogo-A in ameloblasts is localized at the ER and at the border of the ER with the nuclear membrane.

### Nogo-A interactome suggests involvement in proteins nuclear localisation and modulation of gene expression

We set out to investigate in detail the localisation of Nogo-A and its potential molecular partners by determining its interactome in the dental epithelium. For this purpose, we isolated the dental epithelium of lower incisors from *wild type* mouse pups (PN6) and performed total protein extracts. We then immuno-precipitated Nogo-A with the widely validated anti-Nogo-A 11C7 antibody^1^ and performed a mass spectrometry analysis on the eluted protein fraction. Immunoprecipitation with matched isotype IgGs was used as a control (Fig. 3f). We used a highly stringent approach, as we considered putative interactors only proteins immunoprecipitated in duplicate with the 11C7 antibody, but not with the control IgG. This approach allowed us to identify 163 proteins as Nogo-A putative interactors (Supplementary Fig. S3, Supplementary File S1). This group of proteins is divided into four main functional hubs: spliceosome, ribosome, components of the mTOR/PI3K/Akt pathway, and heat shock proteins (Supplementary Fig. S3). Notably, gene ontology analysis highlighted the presence in the Nogo-A interactome of many molecules involved in the regulation of gene expression, in the nuclear localisation of transcription factors, and in regulation of protein localisation to chromosomes (Fig. 3f).

### Deletion of Nogo-A induces alterations in the nuclear and cytoplasmic localisation of many proteins

The interactome of Nogo-A in dental epithelial cells highlights a prevalently intracellular localisation and a strong interaction with proteins destined to the nucleus or involved in protein nuclear localisation. Based on these observations, we investigated whether the deletion of Nogo-A in the dental epithelium leads to alterations in the relative cytoplasmic or nuclear localisation of proteins. For this purpose, we isolated ameloblasts from the lower incisors of *K14;Nogo-A* PN6 pups and from control littermates. We then performed nuclear vs cytoplasm fractionation and subjected the two phases to proteomics analysis (Fig. 3g). We observed alterations in the relative nuclear/cytoplasmic distribution of several classes of proteins upon Nogo-A deletion. Proteins involved in fundamental processes, such as protein localisation to the endoplasmic reticulum or to the membranes, were enriched in the cytoplasm and depleted in the nucleus of *K14;Nogo-A* ameloblasts, when compared to wild type ameloblasts (Fig. 3g, h). Conversely, proteins involved in cell cycle regulation and mediation of key signalling pathways such as Wnt were reduced in the cytoplasm and enriched in the nucleus of *K14;Nogo-A* ameloblasts compared to control littermates (Fig. 3g, h). Taken together, these results suggest that deletion of Nogo-A affects the localisation and transport of proteins between the cytoplasm and the nucleus.

### Nogo-A regulates the expression of genes encoding proteins required for ameloblasts differentiation and enamel formation

**Fig. 4:**
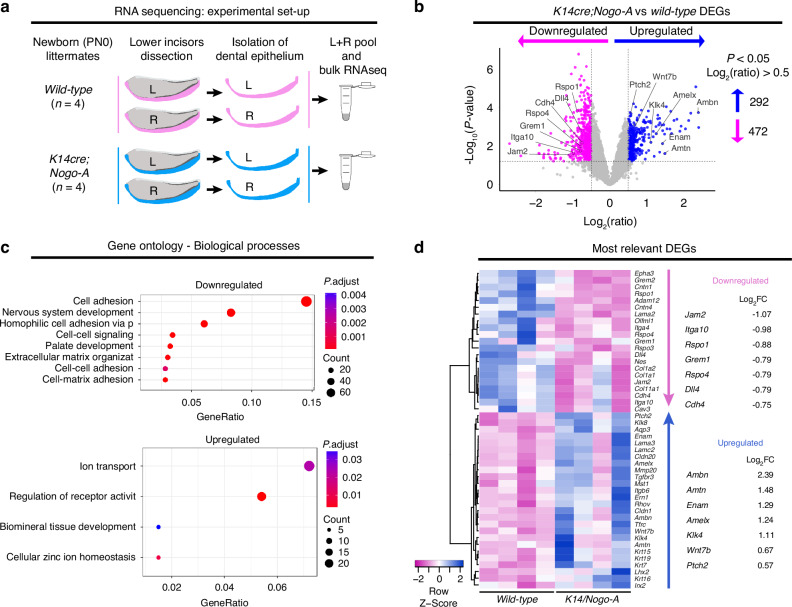
Nogo-A regulates the expression of gene clusters. **a** Experimental strategy. *n* = 4 K14;Nogo-A and *n* = 4 wild-type (control) new-born pups were analysed. The dental epithelia isolated from the left and right lower incisors of each pup were pooled and processed as one replicate. **b** Volcano plot showing significantly downregulated (red arrow) and upregulated (green arrow) genes in K14;Nogo-A dental epithelium. The thresholds were *P* < 0.05 and log_2_ (ratio) > 0.5. **c** Gene ontology analysis showing the biological processes significantly upregulated (up) and downregulated (down). **d** Heatmap showing selected differentially expressed genes (DEGs). Values on the right report the log_2_FC (K14cre;Nogo-A / wild-type) for selected genes

Based on these results, we investigated whether Nogo-A deletion could induce relevant gene expression alterations. For this purpose, we isolated the dental epithelium from the lower incisors of *K14;Nogo-A* PN0 pups and control littermates (*n* = 4 animals per genotype) and analysed their transcriptome by RNA sequencing (Fig. 4a). Deletion of Nogo-A led to a significant deregulation of a high number of genes ( >700; Fig. 4b). Gene ontology analysis highlighted a global downregulation of genes coding for proteins involved in cell-cell and cell-matrix adhesion, as well as in the organization of the extracellular matrix, all features in accordance with the phenotypes observed (Fig. 4c, d; Supplementary Fig. S4). Moreover, genes coding for few members of the Wnt signalling pathway, such as *Rspo1*, *Rspo3*, and *Rspo4*, as well as for the Notch ligand Dll4 were downregulated in the dental epithelium of *K14;Nogo-A* PN0 pups (Fig. 4d, Supplementary Figure S4). Conversely, Nogo-A deletion led to a general upregulation of genes coding for proteins involved in ion transport and biomineral tissue development (Fig. 4c), both processes strongly involved in ameloblasts differentiation.^28^ Indeed, we observed that most key ameloblast differentiation markers, such as *Amelx*, *Ambn*, *Enam*, *Mmp20*, and *Klk4*, were strongly upregulated (Fig. 4d; Supplementary Fig. S4). The upregulation of differentiation markers was not accompanied by a downregulation of the expression of classical dental epithelial stem cell markers, such as *Sox2*, *Bmi1*, *Lgr5*, and *Gli1*. Few genes coding for members of signalling pathways involved in ameloblast differentiation were also affected: among these, *Fgf13* and *Fgf2* were downregulated, while the Shh receptor *Ptch2*, as well as *Wnt7b* were upregulated (Supplementary Fig. S4).

### Intracellular Nogo-A, and not cell-membrane Nogo-A, is responsible for gene expression modulation

The evidence presented above, together with the epithelial Nogo-A expression pattern, suggest a role for Nogo-A in the expression of genes relevant for ameloblast differentiation. While Nogo-A has been shown to act predominantly by signalling from its localisation on the cell membrane to adjacent Nogo receptor-expressing cells,^16^ a large fraction of Nogo-A molecules is localised intracellularly, and it may affect gene expression in a cell-autonomous manner. To distinguish between a signalling or a cell-autonomous role of Nogo-A, we set out to investigate whether the significant impact of Nogo-A deletion on ameloblasts transcriptome is mediated by intracellular or cell membrane-bound Nogo-A. For this purpose, we employed LS8 mouse cells, which is a highly representative line of preameloblasts.^35^

**Fig. 5:**
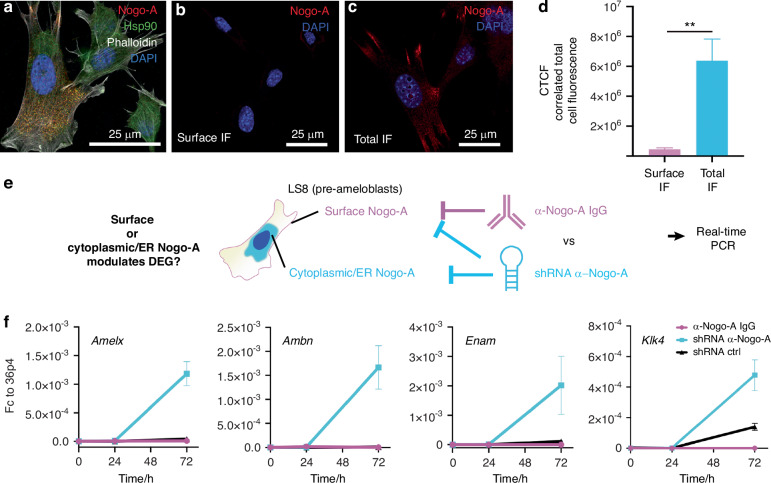
Inhibition of intracellular-, and not extracellular membrane bound, Nogo-A is responsible for gene expression modulation. **a** Immunofluorescent staining showing expression of Nogo-A (red colour) and Hsp90 (green colour) in LS8 cells. White colour: phalloidin; blue colour: DAPI. Immunofluorescent staining showing surface-bound (**b**) and total (**c**) Nogo-A (red colour) in LS8 cells. Blue colour: DAPI. **d** Quantification of correlated total cell fluorescence in (**b**) and (**c**). **e** Experimental strategy to determine whether cell membrane-bound or intracellular Nogo-A mediates the regulation of ameloblastic differentiation genes. **f** Real-time PCR analysis of the expression of *Amelogenin* (*Amelx*), *Ameloblastin* (*Ambn*), *Enamelin* (*Enam*) and *Kallikrein 4* (*Klk4*) upon antibody-mediated inhibition of membrane-bound Nogo-A (α-Nogo-A IgG, magenta line) or overall Nogo-A knockdown (shRNA α-Nogo-A, light blue line). Gene expression is expressed as a fold-change over the *36B4* housekeeping gene. The graph represents the mean and standard deviation (*n* = 3)

We first performed a double immunofluorescent staining against the Nogo-A and Hsp90 interactor identified within the Nogo-A interactome (Fig. 3). The staining revealed that both proteins are expressed by LS8 cells (Fig. 5a), and high-resolution confocal imaging showed that Nogo-A and Hsp90 colocalize in the cytoplasm in ER-like structures and stress fibres (Fig. 5a; Supplementary Fig. S5a–e), confirming the intracellular interaction between Nogo-A and Hsp90 in the ameloblast lineage. We further assessed Nogo-A cellular localisation by staining selectively for the fraction of Nogo-A localised at the extracellular membrane (see Materials and Methods). We observed that only a very minor fraction of Nogo-A was expressed on the plasma membrane, while most Nogo-A was localized intracellularly (Intracellular Nogo-A: Cell surface Nogo-A = 15:1; Fig. 5b–d). To assess the functional relevance of the interaction between Nogo-A and Hsp90 at this intracellular location, we investigated whether Nogo-A deletion affects the nuclear localization of the transcription factor Hsf1, effector of Hsp90.^36^ Notably, the genomic loci of *Amelx, Ambn, Enam*, and *Klk4* contain several potential Hsf1 binding sites (Supplementary File S2). We thus knocked-down Nogo-A expression via shRNA and assessed Hsf1 localization via immunostaining and confocal microscopy (Supplementary Fig. S5f–i). Quantification of the relative nuclear and cytoplasmic abundance of Hsf1 showed a variable yet significant increase in its nuclear localization upon Nogo-A depletion (Supplementary Fig. S5i). These findings suggest that loss of Nogo-A can affect the function of Hsp90, which results in the increased nuclear localization of its effector Hsf1.

We then set out to investigate whether loss of cell membrane-bound or intracellular Nogo-A is ultimately responsible for the overexpression of ameloblast differentiation markers observed upon Nogo-A deletion in vivo. To this end, we selectively inhibited cell membrane-bound Nogo-A via treatment with the anti-Nogo-A antibody 11C7, or blocked all Nogo-A expression (cell membrane-bound and intracellular) via shRNA, and assessed the effect on the expression of the ameloblast differentiation markers *Amelx*, *Ambn*, *Enam* and *Klk4* (Fig. 5e). We observed that inhibition of only cell-membrane bound Nogo-A did not have any effect on gene expression (Fig. 5f). On the contrary, shRNA-mediated inhibition of Nogo-A led to the significant upregulation of *Amelx*, *Ambn*, *Enam*, and *Klk4* (Fig. 5f), faithfully recapitulating the effects of Nogo-A deletion in the dental epithelium in vivo. Taken together, these results indicate that the intracellular Nogo-A, and not the cell membrane Nogo-A, is responsible for the modulation of the expression of genes important for ameloblast differentiation.

## Discussion

Numerous studies have highlighted the importance of Nogo-A in the regulation of key processes involved in the development and regeneration of neural and vascular systems.^4,21^ Our latest studies further involved Nogo-A in the modulation of trigeminal innervation and human dental pulp stem cell differentiation.^25,26^ Herein, we report a new, unexpected function of Nogo-A in the formation of tooth enamel, the hardest tissue of the body. We indeed observed that Nogo-A is expressed in teeth during embryonic development and in postnatal life, as well as in a number of organs of the craniofacial complex, confirming and significantly expanding previous observations^37^ Analysis of teeth in mice lacking Nogo-A, either systemically or specifically in the epithelium, showed disorganisation of enamel-secreting ameloblasts accompanied by disordered prisms that greatly compromised the structure of enamel. It is probable that these important enamel modifications are due to the disorganization of the ameloblastic layer. Enamel formation requires the regulated secretion of enamel-specific proteins, the deposition of minerals and the formation of crystals, followed by the degradation of the deposited enamel matrix proteins by metalloproteases, which are secreted by ameloblasts at the very last stage of amelogenesis.^28^ Alterations in each of these processes can lead to the generation of defective enamel.^28,38^ So far, enamel defects have been mostly associated with loss-of-function mutations in genes coding for enamel matrix proteins such as amelogenin (*AMELX*)^39^ and enamelin (*ENAM*),^40^ as well as proteases such as enamelysin (*MMP20*) and kallikrein-4 (*KLK4*).^41^ Interestingly, Nogo-A deletion upregulated the expression of all these genes involved in amelogenesis. Excessive production of enamel matrix proteins, such as ameloblastin, can induce the formation of hypomineralized enamel. This could occur through a combination of mechanisms, including the alteration of ameloblast maturation timing, protein secretion, and detachment.^42^ Indeed, Nogo-A deletion, or shRNA-mediated knockdown, induced the overexpression of several enamel matrix genes that might cause enamel defects. A concomitant increase in genes coding for enamel matrix metalloproteases, and a broad alteration in the expression of genes coding for cell-cell adhesion molecules, indicates a more complex and multifaceted role for Nogo-A. Nevertheless, the increased expression of genes coding for enamel matrix proteins and proteases in ameloblasts does not affect the total volume of enamel deposition in the Nogo-A mutant mice. By contrast, the enamel of these mice is characterised by alterations in the structure and disposition of the enamel prisms (or rods). Similarly, altered levels of biochemical key components (iron and calcium) are detected in the enamel of Nogo-A mutant mice. All these modifications affect and fragilize the enamel, thus favouring carious infections and enamel fractures.^28^ Nogo-A thus constitutes an important player during amelogenesis by regulating the expression of genes involved in enamel synthesis and deposition. Based on these results, we focused our attention on the possible mechanism by which Nogo-A can affect amelogenesis. The vast majority of the studies investigating Nogo-A function focused on its signalling action from the cell surface to specific receptors,^3,7,11,37,43^ although this molecule is largely localised intracellularly (at the endoplasmic reticulum, ER.^5,44,45^). Only a few studies investigated the function of intracellular Nogo-A, suggesting its implication in modulating ER curvature,^5,44^ as well as in regulating ErbB3 signalling, a pathway activated by many growth factors and important in the regulation of cell proliferation, survival, differentiation.^45^ To better understand the mode of action of Nogo-A in ameloblasts, we analysed its interaction with other molecular players by pulldown assays. We found that a significant number of cytosolic proteins interacting with Nogo-A are involved in the regulation of gene expression, and thus hypothesised that the interaction between intracellular Nogo-A and these cytosolic molecules can be responsible for the strong alterations in gene expression in the dental epithelium of Nogo-A mutant mice. Indeed, the differential mass spectrometry analysis suggests that Nogo-A deletion affects the nuclear or cytoplasmic localisation of different categories of proteins, including factors directly involved in gene expression regulation and transcription. Our hypothesis was further strengthened by the demonstration that the antibody-mediated inhibition of cell surface-Nogo-A was not sufficient to induce upregulation of genes involved in ameloblasts differentiation, while knockdown also of intracellular Nogo-A effectively recapitulated the gene expression alterations induced by Nogo-A deletion. Nogo-A strongly interacts also with the spliceosome and ribosomes, which are two other key cellular components, suggesting additional roles for Nogo-A in important processes such as splicing and translation. The function of Nogo-A during amelogenesis could also be in part mediated by its interaction with specific Nogo-A receptors. While S1PR2 is expressed in differentiated ameloblasts, neither S1PR2 nor NgR1 are expressed by preameloblasts, i.e., the cells where we observed the upregulation of differentiation markers upon Nogo-A deletion. By contrast, P75/NGFR is expressed by preameloblasts. Co-expression of P75/NGFR and NgR1 is however generally required to mediate Nogo-A-dependent signalling. Recent studies proposed a large range of potential receptors for Nogo-A.^7,8,43^ Among these, syndecan-3 and -4 have been reported to be expressed in ameloblasts of rodent incisors, while they are not expressed in preameloblasts.^46^ Overall, these results and the relative expression patterns suggest that the observed functions of Nogo-A in enamel formation are not mediated by these receptors.

**Fig. 6:**
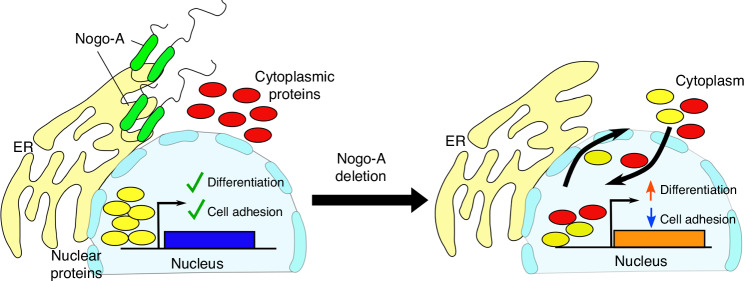
Hypothetical mode of action of Nogo-A in the dental epithelium. Nogo-A can modulate the relative nuclear /cytoplasmic localisation of classes of transcription factors, allowing the timely differentiation of dental epithelial cells into ameloblasts. Deletion of Nogo-A results in aberrant nuclear/cytoplasmic localisation of proteins involved in gene expression regulation, which in turn results in massive alterations in gene expression. As a result, dental epithelial cells overexpress genes driving ameloblast differentiation and downregulate, among others, genes encoding for proteins mediating cell adhesion. This, in turn, leads to the production of defective enamel

Taken together, our results show a completely new function for Nogo-A as modulator of amelogenesis. At the same time, the characterization of its in vivo interactome suggested potential new mechanisms of action (Fig. 6). Among these, we show that intracellular Nogo-A could modulate the expression of specific gene clusters. These new molecular functions could constitute a general feature of Nogo-A that has so far been overlooked. More generally, our work emphasizes the importance of molecules and pathways that act in the later phases of organ development and cytodifferentiation, fine-tuning their outcome. Such fine-tuning of tissue and cell function is often overlooked, but it is fundamental to create highly complex organs and might represent a key basis for individual biological variability and differential susceptibility to pathologies observed among the human population.

## Materials and methods

### Animal handling

All experiments were performed according to the guidelines of the Swiss Animal Welfare Law and in compliance with the regulations of the Cantonal Veterinary Office, Zurich (License for animal experimentation ZH151/2014; ZH146/17; ZH203/2020). The animal facility provided standardized housing conditions, with a mean room temperature of 21 °C ± 1 °C, relative humidity of 50% ± 5%, and 15 complete changes of filtered air per hour (HEPA H 14filter); air pressure was controlled at 50 Pa. The light/dark cycle in the animal rooms was set to a 12 h/12 h cycle (lights on at 07:00, lights off at 19:00) with artificial light of approximately 40 Lux in the cage. The animals had unrestricted access to sterilized drinking water, and ad libitum access to a pelleted and extruded mouse diet in the food hopper (Kliba No. 3436; Provimi Kliba/Granovit AG, Kaiseraugst, Switzerland). Mice were housed in a barrier-protected specific pathogen-free unit and were kept in groups of maximum 5 adult mice per cage in standard IVC cages (Allentown Mouse 500; 194 mm × 181 mm × 398 mm, floor area 500 cm^2^; Allentown, New Jersey, USA) with autoclaved dust-free poplar bedding (JRS GmbH + Co KG, Rosenberg, Germany). A standard cardboard house (Ketchum Manufacturing, Brockville, Canada) served as a shelter, and tissue papers were provided as nesting material. Additionally, crinklets (SAFE® crinklets natural, JRS GmbH + Co KG, Rosenberg, Germany) were provided as enrichment and further nesting material. The specific pathogen-free status of the animals was monitored frequently and confirmed according to FELASA guidelines by a sentinel program. The mice were free of all viral, bacterial, and parasitic pathogens listed in FELASA recommendations.^47^ *Nogo-A KO* (Nogo-A -/-, B6-RTN4A<tm1/Schwab>^30^) and *Nogo-A fl/fl* mice (C57BL/6-Rtn4tm2175Arte^48^ were generated by the Schwab lab. *K14cre;Nogo-A fl/fl* mice were obtained by crossing *Nogo-A fl/fl* (C57BL/6-Rtn4tm2175Arte) mice with K14cre (Tg(KRT14-cre)1Amc; MGI: 2445832) mice. Mouse pups younger than postnatal day 10 (PN10) were sacrificed by decapitation. PN20 and older mice were anesthetized with Ketamine/Xylazine and sacrificed by intracardiac perfusion with phosphate-buffered saline (PBS) followed by Paraformaldehyde (PFA) 4% in PBS. Mouse heads were then separated and post-fixed in PFA 4% overnight at 4 °C. The specimens were further washed with PBS and processed according to the application.

### Immunohistochemistry, immunofluorescent staining, in situ hybridisation

5 μm Paraffin sections were rehydrated by incubation in Xylol followed by a series of Ethanol solutions (100% to 30%) and distilled H_2_O. For immunohistochemistry only, endogenous Peroxidase was quenched by incubation for 30 min, at 4 °C, in 0.3% H_2_O_2_ diluted in Methanol. Thereafter, specimens were blocked with PBS supplemented with 2% Fetal Bovine Serum (FBS) and incubated with primary antibodies for 1 h at room temperature. The following primary antibodies were used: human anti-Nogo-A (dilution 1:300),^1^ rabbit anti-S1PR2 (dilution 1:100; 4385-AP01311PU-N; OriGene Technologies, Rockville, MD, USA), goat anti-NgR1 (dilution 1:100; AF1440, R&D Systems, Minneapolis, MI, USA), rabbit anti-P75/NGFR (dilution 1:200; N3908, Sigma Aldrich, Buchs, Switzerland). The sections were then incubated either with Fluorochrome-conjugated secondary antibodies or with biotinylated secondary antibodies for 1 h at room temperature. The following secondary antibodies were used: biotinylated anti-human (dilution 1:1 000; 709-065-149, Jackson ImmunoResearch, Switzerland), Alexa 568-conjugated anti-rabbit (dilution 1:500) Alexa-647 conjugated anti-mouse (dilution 1:500), Alexa-568-conjugated anti-goat (dilution 1:500) (ThermoFisher Scientific, Basel, Switzerland). For immunohistochemistry, sections were incubated in ABC reagent (Vector Labs, Burlingame, USA) and developed with the AEC detection kit (Vector Labs, Burlingame, USA). Slides were counterstained with Toluidine blue and mounted with Glycergel (DAKO/Agilent, Santa Clara, CA, USA). For immunofluorescent nuclear staining, 4’,6-Diamidino-2-Phenylindole (DAPI; D1306, Thermo Fisher Scientific, Reinach, Switzerland) was used. After immunofluorescent staining, samples were mounted in ProLong™ Diamond Antifade Mountant (P36965, Thermo Fisher Scientific, Reinach, Switzerland), and imaged with a Leica SP8 Inverted Confocal Laser Scanning Microscope (Leica Microsystems- Schweiz AG, Heerbrugg, Switzerland).

Anti-*Nogo-A* riboprobes were used for in situ hybridization on 14 μm cryosections, as previously described.^49^ The hybridization signal was detected using the NBT/BCIP substrate solution, sections were mounted with Glicergel® mounting medium (Dako) and imaged with Leica DM6000 microscope equipped with the Leica DFC420C camera. Images were processed with the Leica Application Suite (LAS) software.

### Scanning electron microscopy

Fully mineralized lower hemi-jaws were dissected from perfused adult (two months old) wild-type (*n* = 6), *Nogo-A KO* (*n* = 6) and *K14cre; Nogo-A fl/fl* (*n* = 3) mice. Soft tissues were removed manually and by incubating the samples in 3% H_2_O_2_ overnight. The mandibles were then dehydrated and embedded in Technovit 7200 VLC (Heraeus Kulzer, Wehrheim, Germany). Light-polymerized blocks were mounted on Aluminium stubs, polished and coated with a 10–15 nm thick layer of Carbon, and finally examined using a Tescan EGATS5316 XMSEM (Tescan, Brno, Czech Republic) operated in BSE mode. Elemental composition of enamel was analysed with the aid of energy-dispersive X-ray spectroscopy (EDS). A Si(Li) detector (Oxford Instruments, Wiesbaden, Germany) served for recording EDS spectra using an accelerating voltage of 7 kV, a working distance of 23 mm, and a counting time of 100 s. For the quantitative analysis of these spectra, the Inca energy software (Oxford Instruments) was used. Comparisons between EDS of wild type and Nogo-A KO lower incisors enamel were performed on *n* = 4 animals per condition; abundance of each element in the two conditions was compared via two-tailed *t*-test (Graph Pad Prism 8.0).

### Transmission electron microscopy (TEM)

Lower hemi-jaws were dissected from perfused adult (approx. 3 months of age) wild type (*n* = 3) and Nogo-A KO (*n* = 3) mice and decalcified in 10% EDTA (pH 7.4) for 10 weeks. The samples were then postfixed in 1.33% Os-tetraoxide in 0.067 M s-collidine buffer for 2 h at room temperature. Thereafter, they were dehydrated in ethanol, transferred to propylene oxide and embedded in Epon 812 (Fluka, Buchs, Switzerland). From the resin blocks, thin sections of 80–100 nm in thickness were cut using a Reichert Ultracut ultramicrotome (Leica Microsystems, Heerbrugg, Switzerland) and diamond knives (Diatome, Biel, Switzerland). Sections were collected on copper grids, contrasted with U-acetate and Pb-citrate, and examined in a Philips EM400 T TEM (FEI, Eindhoven, Netherlands) at 60 kV. Micrographs were recorded using a Hamamatsu ORCA-HR camera (Hamamatsu Photonics, Hamamatsu, Japan) and the AMT image acquisition software (Deben, Bury St. Edmunds, UK).

### RNA sequencing

Samples were isolated from lower incisors freshly dissected from new-born (PN0) wild-type and *K14cre;Nogo-A fl/fl* pups. The dental epithelium was isolated by incubating the incisors in Dispase (2 mg/ml in HBSS; D4818, Sigma Aldrich, Buchs, Switzerland). RNA was isolated using the RNeasy Plus Mini Kit (Qiagen AG, Switzerland) and subsequently purified by Ethanol precipitation. The quality of the isolated RNA was determined with a Qubit® (1.0) Fluorometer (Life Technologies, California, USA) and a Bioanalyzer 2100 (Agilent, Waldbronn, Germany). Only those samples with a 260 nm/280 nm ratio between 1.8–2.1, 28S/18S ratio within 1.5-2, and a RIN (RNA integrity number) of >7 were further processed. The TruSeq RNA Sample Prep Kit v2 (Illumina, Inc, California, USA) was used in the succeeding steps. Briefly, total RNA samples (100–1 000 ng) were Poly A enriched and then reverse-transcribed into double-stranded cDNA. The cDNA samples were fragmented, end-repaired and polyadenylated before ligation of TruSeq adapters containing the index for multiplexing Fragments containing TruSeq adapters on both ends were selectively enriched with PCR. The quality and quantity of the enriched libraries were validated using Qubit® (1.0) Fluorometer and the Caliper GX LabChip® GX (Caliper Life Sciences, Inc., USA). The product is a smear with an average fragment size of approximately 260 bp. The libraries were normalized to 10 nmol/L in Tris-Cl 10 mmol/L, pH8.5 with 0.1% Tween 20. The TruSeq PE Cluster Kit HS4000 or TruSeq SR Cluster Kit HS4000 (Illumina, Inc, California, USA) was used for cluster generation using 10 pmol/L of pooled normalized libraries on the cBOT. Sequencing was performed on the Illumina HiSeq 4000 single end 125 bp using the TruSeq SBS Kit HS4000 (Illumina, Inc, California, USA). Individual library size ranged from 30 million to 50 million reads. RNA sequencing analysis was performed using the SUSHI framework,^50^ which encompassed the following steps: read quality was inspected using FastQC, and sequencing adaptors removed using fastp^51^; Alignment of the RNA-Seq reads using the STAR aligner^52^ and with the Ensembl Mus musculus genome build GRCm38 (patch 5, Release 91) as the ref. ^53^; the counting of gene-level expression values using the ‘featureCounts’ function of the R package Rsubread^54^; differential expression using the generalised linear model as implemented by the edgeR Bioconductor R package and normalized with the edgeR trimmed mean of M-values (TMM)^55^ and; Gene Ontology (GO) term pathway analysis using both the hypergeometric over-representation test via the ‘enricher’ function, and gene-set enrichment analysis via the ‘GSEA’ function, of the clusterProfiler Bioconductor R package.^56^ Differential abundance of splicing isoforms was quantified with sqSeq.^57^ All R functions were executed on R version 3.5 (R Core Team, 2020) and Bioconductor version 3.7. A gene is marked as DE if it possesses the following characteristics: i) at least 10 counts in at least half of the samples in one group; ii) *P* < = 0.05; iii) fold change ≥ 0.5. Finally, gene sets were used to interrogate the GO Biological Processes database for an exploratory functional analysis. Contingency tables were constructed based on the number of significant and non-significant genes in the categories and we reported statistical significance using Fisher’s exact test. RNA-sequencing data are available at https://www.ncbi.nlm.nih.gov/geo/query/acc.cgi?acc=GSE160829.

### Immunoprecipitation and mass spectrometry for interactome characterization

Dissected dental epithelia were minced in cold PBS and treated with a hypotonic lysis buffer (20 mmol/L tris-HCl, 75 mmol/L NaCl, 1.5 mmol/L MgCl_2_, 1 mmol/L EGTA, 0.5% NP-40, and 5% Glycerol). The obtained protein extracts were incubated with 1 mg of 11C7 anti-Nogo-A mouse monoclonal antibody^1^ and protein A-conjugated Sepharose beads (GE Healthcare); they were then diluted in lysis buffer to a final volume of 1 mL. After 4 h of incubation at 4 °C on a rotating wheel, the beads were spun down and washed three times in lysis buffer. All steps were performed on ice, and all buffers were supplemented with fresh Protease inhibitors (cOmplete, Roche) and 1 mmol/L Phenylmethylsulfonyl Fluoride. The samples were then analysed by liquid chromatography-MS/MS analysis. For liquid chromatography–MS/MS analysis, the protein samples, already dissolved in Laemmli buffer, were submitted to a filter-aided sample preparation (FASP)^58^ and digested with Trypsin in 100 mmol/L Triethylammonium Bicarbonate buffer overnight. Desalted samples were dried completely in a vacuum centrifuge and reconstituted with 50 mL of 3% Acetonitrile and 0.1% Formic Acid. Each peptide solution (4 mL) was analysed on both Q Exactive and Fusion mass spectrometers (Thermo Scientific) coupled to EASY-nLC 1000 (Thermo Scientific). On the Q Exactive, full-scan MS spectra were acquired in profile mode from 300 to 1 700 mass/charge ratio (m/z) with an automatic gain control target of 3 Å~ 106, an Orbitrap resolution of 70 000 (at 200 m/z), and a maximum injection time of 120 ms. The 12 most intense multiply charged (z = +2 to +8) precursor ions from each full scan were selected for higher-energy collisional dissociation fragmentation with a normalized collision energy of 30 arbitrary unit. Generated fragment ions were scanned with an Orbitrap resolution of 35 000 (at 200 m/z), an automatic gain control value of 5 Å~ 104, and a maximum injection time of 120 ms. The isolation window for precursor ions was set to 2.0 m/z, and the underfill ratio was at 1% (referring to an intensity of 4.2 Å~ 103). Each fragmented precursor ion was set onto the dynamic exclusion list for 90 s. For the Orbitrap Fusion, the “Universal Method” as described in^59^ was applied. Peptide separation on both instruments was achieved by reversed-phase high-performance liquid chromatography on an in-house packed C18 column (150 mm Å~75 mm, 1.9 mm, C-18 AQ, 120 Å; Dr. Maisch GmbH). Samples were loaded with maximum speed at a pressure restriction of 400 bar and separated with a linear gradient from 3 to 25% solvent B (0.1% Formic Acid in Acetonitrile, Biosolve BV) in solvent A (0.1% Formic Acid in H_2_O, Biosolve BV) at a flow rate of 250 nL/min. The column was washed after the separation by flushing with 95% solvent B for 10 min, automatically equilibrated before the next injection, and exported to mgf (Mascot generic format) format for subsequent database search. Peak lists were extracted from the instrument raw files using Proteome Discoverer (version 1.4) in combination with the FCC [Functional Genomics Centre of Zurich (FGCZ) Converter Control]^60^ and exported to mgf format for subsequent database search. All MS/MS samples were analysed using Mascot software (Matrix Science, version 2.4.1). The parameters were set up to search the fgcz_10090_20140715 database (https://fgcz-proteomics.uzh.ch/fasta/fgcz_10090_20140715.fasta, 51 806 entries), with Trypsin indicated as the digestion enzyme, and the fgcz_10090_d_20140715 database (https://fgcz-proteomics.uzh.ch/fasta/fgcz_10090_d_20140715.fasta, 103 351 entries), also assuming Trypsin. Mascot was searched with a fragment ion mass tolerance of 0.050 Da and a parent ion tolerance of 10.0 parts per million. Carbamidomethyl of Cysteine was specified in Mascot as a fixed modification. Oxidation of Methionine was specified in Mascot as a variable modification. Scaffold (version Scaffold_4.4.1, Proteome Software Inc.) was used to validate MS/MS-based peptide and protein identifications. Peptide identifications were accepted if they could be established at greater than 95% probability by the Peptide Prophet algorithm^61^ with Scaffold delta-mass correction. Protein identifications were accepted if they could be established at greater than 95.0% probability and contained at least two identified peptides. Protein probabilities were assigned by the Protein Prophet algorithm.^62^ Proteins that contained similar peptides and could not be differentiated on the basis of MS/MS analysis alone were grouped to satisfy the principles of parsimony. Proteins sharing significant peptide evidence were grouped into clusters. Gene Ontology^63,64^ was used to analyse the cellular localisation and the participation in biological processes of Nogo-A interactors.

### Nuclear / cytoplasm differential mass spectrometry

Dental epithelia were isolated from the lower incisors of PN6 *K14cre; Nogo-A* pups and control littermates. Cytoplasmic versus nuclear protein fractionation was performed as previously described.^38,65^ For each sample, the total provided volume was taken and reduced with 2 mmol/L Tris(2-CarboxyEthyl)Phosphine (TCEP) and alkylated with 15 mmol/L Iodoacetamide at 60 °C for 30 min. The sp3 protein purification, digest, and peptide clean-up was performed using a Kingfisher Flex System (Thermo Fisher Scientific) and Carboxylate-Modified Magnetic Particles (GE Life Sciences).^66,67^ Beads were conditioned following the manufacturer’s instructions, consisting of 3 washes with water at a concentration of 1 µg/µL. Samples were diluted with an equal volume of 100% Ethanol (50% Ethanol final concentration). The beads, wash solutions and samples were loaded into 96 deep well-plates or micro-plates and transferred to the Kingfisher.

Following steps were carried out on the robot: collection of beads from the last wash, protein binding to beads (14 min), washing of beads in wash solutions 1–3 (80% Ethanol, 3 min each), protein digestion (for 4 h at 37 °C with a trypsin/protein ratio of 1:50 in 50 mmol/L TEAB) and peptide elution from the magnetic beads (water, 6 min). The digest solution and water elution were combined and dried to completeness. Afterwards the peptides were re-solubilized with 20 µL of 3% acetonitrile, 0.1% formic acid and 3 µL of indexed retention time (iRT)-peptides (Biognosys) were spiked in each sample for MS analysis. The peptide concentration was determined using a Lunatic (Unchained Labs) instrument. Mass spectrometry analysis was performed on an Orbitrap Fusion Lumos (Thermo Scientific) equipped with a Digital Picoview source (New Objective) and coupled to a M-Class UPLC (Waters). Solvent composition of the two channels was 0.1% Formic Acid for channel A and 0.1% Formic Acid, 99.9% Acetonitrile for channel B. For each sample 0.1 abs of peptides were loaded on a commercial MZ Symmetry C18 Trap Column (100 Å, 5 µm, 180 µm x 20 mm, Waters) followed by nanoEase MZ C18 HSS T3 Column (100 Å, 1.8 µm, 75 µm x 250 mm, Waters). The peptides were eluted at a flow rate of 300 nL/min. After an initial hold at 5% B for 3 min, a gradient from 5 to 24% B in 80 min and 36% B in 10 min was applied. The column was washed with 95% B for 10 min, and afterward, the column was re-equilibrated to starting conditions for an additional 10 min. Samples were acquired in a randomized order. The mass spectrometer was operated in data-dependent mode (DDA), acquiring full-scan MS spectra (300 − 1 500 m/z) at a resolution of 120 000 at 200 m/z after accumulation to a target value of 500 000. Data-dependent MS/MS were recorded in the linear ion trap using quadrupole isolation with a window of 0.8 Da and HCD fragmentation with 35% fragmentation energy. The ion trap was operated in rapid scan mode with a target value of 10 000 and a maximum injection time of 50 ms. Only precursors with intensity above 5 000 were selected for MS/MS, and the maximum cycle time was set to 3 s. Charge state screening was enabled. Singly, unassigned, and charge states higher than seven were rejected. Precursor masses previously selected for MS/MS measurement were excluded from further selection for 20 s, and the exclusion window was set at 10 ppm. The samples were acquired using internal lock mass calibration on m/z 371.1012 and 445.1200. The mass spectrometry proteomics data were handled using the local laboratory information management system (LIMS).^68^ The acquired raw MS data were processed by MaxQuant (version 1.6.2.3), followed by protein identification using the integrated Andromeda search engine.^69^ Spectra were searched against a mouse reference proteome (version from 2019-07-09), concatenated to its decoyed fasta database and common protein contaminants. Carbamidomethylation of Cysteine was set as fixed modification, while Methionine oxidation and N-terminal protein acetylation were set as variable. Enzyme specificity was set to Trypsin/P, allowing a minimal peptide length of 7 amino acids and a maximum of two missed cleavages. MaxQuant Orbitrap default search settings were used. The maximum false discovery rate (FDR) was set to 0.01 for peptides and 0.05 for proteins. Label-free quantification was enabled, and a 2 min window for match between runs was applied. In the MaxQuant experimental design template, each file is kept separate in the experimental design to obtain individual quantitative values. Protein fold changes were computed based on Intensity values reported in the proteinGroups.txt file. A set of functions implemented in the R package SRMService (https://rdrr.io/github/protViz/SRMService/) was used to filter for proteins with two or more peptides. A modified robust z-score transformation was applied to remove differences among the sample. Finally, *p*-values were computed using the t-test with empirical Bayes variance shrinkage.^70^ If all protein measurements are missing in one of the conditions, a pseudo fold change was computed, replacing the missing group average by the mean of 10% smallest protein intensities in that condition. In addition, we performed gene set enrichment analysis using the R/Bioconductor package fgsea (10.18129/B9.bioc.fgsea) and used gene sets specified in the molecular signature database (http://www.gsea-msigdb.org/). To apply GSEA to proteomics data, we mapped the UniProt identifiers to Entrez Id’s using the UniProt mapping service. Then, we ordered the protein lists using the log2FC. For cases where several UniProt Id’s were mapped to a single Entrez Id, we averaged the log2FC. All MS data have been deposited in the PRIDE (Proteomics Identifications) Archive database,^71^ with accession number PXD030386.

### Identification of potential Hsf1 binding sites

Potential binding sites were identified using the FIMO motif scanning package.^72^ As input, we selected the sequences of the *Amelx*, *Ambn*, *Enam*, and *Klk4* mouse genes, plus 1 000 base pairs upstream of the TSS and downstream of the annotated loci to include the promoter region and potential proximal downstream regulatory sequences. Hsf1 motifs were obtained from Motifmap.^73^ Output files are provided within the “Supplementary_File2_FIMO_HSF1motif_search” supplementary file.

### LS8 cell culture and analysis

LS8 cells^35^ were cultured in DMEM (31966047, ThermoFisher Scientific, Basel, Switzerland) supplemented with 10% FBS and 1x Penicillin/Streptomycin (P/S; 15140122, Thermo Fisher Scientific, Basel, Switzerland). For antibody-mediated inhibition of Nogo-A, LS8 cells were treated with 10 μg/mL 11C7 anti-Nogo-A antibody (*n* = 3),^1^ while control cells were treated with 10 μg/mL mIgG1 (*n* = 3). For shRNA-mediated Nogo-A knockdown experiments, LS8 cells were transfected with plasmids for shRNA against Nogo-A and scrambled shRNA control Sigma-Aldrich;SHCLNG, NM_194054 / TRCN0000071689; *n* = 3 shRNA anti-Nogo-A; *n* = 3 scrambled shRNA). Transfections were performed with the JetOPTIMUS® DNA Transfection Reagent (Polyplus Transfection, Illkirch, France). For real time PCR analysis, cells were lysed with the lysis buffer from the RNeasy Mini Plus Kit and processed according to the manufacturer instructions. Reverse transcription of the isolated RNA was performed using the iScript™ cDNA synthesis Kit and according to the instructions given (Bio-Rad Laboratories AG, Cressier FR, Switzerland). Briefly, 1 000 ng of RNA were used for reverse transcription into cDNA. Nuclease-free water was added to add up to a total of 15 μL. 4 μL of 5x iScript reaction mix and 1 μL of iScript reverse transcriptase were added per sample in order to obtain a total volume of 20 μL. The reaction mix was then incubated for 5 min at 25 °C, for 30 min at 42 °C and for 5 min at 85 °C using a Biometra TPersonal Thermocycler (Biometra AG, Göttingen, Germany). The 3-step quantitative real-time PCRs were performed using an Eco Real-Time PCR System (Illumina Inc., San Diego CA, USA). 5 μL of SYBR® Green PCR Master Mix reverse and forward primers (200 nmol/L), and 2 ng of template cDNA were added to each well. The thermocycling conditions were: 95 °C for 10 min, followed by 40 cycles of 95 °C for 15 s, 55 °C for 30 s and 60 °C for 1 min. Melt curve analysis was performed at 95 °C for 15 s, 55 °C for 15 s and 95 °C for 15 s. Expression levels were calculated by the comparative ΔΔCt method (2^−ΔΔCt^ formula), after being normalized to the Ct-value of the housekeeping gene. Analysis: one-way ANOVA, non-parametric, Tukey correction for multiple comparisons (Graph Pad Prism 8.0).

For imaging analysis, at the end of the culture period, cells were fixed with 4% PFA for 10 min, washed in PBS supplemented with 2% BSA for 30 min and then processed for immunofluorescent staining. Cells were incubated with primary antibodies for 1 h at room temperature. The following primary antibodies were used: mouse anti-Nogo-A.^1^ (dilution 1:200), rabbit anti-Hsp90 (dilution 1:200; 4877 T, Cell Signaling Technology, Danvers, MA, USA), rabbit anti-S1PR2 (dilution 1:100;4385-AP01311PU-N; OriGene Technologies, Rockville, MD, USA), rabbit anti-HSF1 (dilution 1:100, ab2923, Abcam - Lucerna-Chem AG, Luzern, Switzerland), goat anti-NgR1 (dilution 1:100; AF1440, R&D Systems, Minneapolis, MI, USA), rabbit anti-P75NGFR (dilution 1:200; N3908, Sigma Aldrich, Buchs, Switzerland). Cells were then washed with PBS and incubated with secondary antibodies for 1 hour at room temperature. The following antibodies were used: Alexa 568-conjugated anti-rabbit dilution 1:500), Alexa-647 conjugated anti-mouse (dilution 1:500), Alexa-568-conjugated anti-goat (dilution 1:500) (ThermoFisher Scientific, Basel, Switzerland). Cells were then counterstained with Alexa-488 conjugated phalloidin (A12379, ThermoFisher Scientific, Basel, Switzerland) and DAPI, and imaged with a Leica LS8 confocal laser scanning microscope. For cell surface Nogo-A immunostaining, cells were incubated, without fixation, in 4 °C PBS supplemented with 10 μg/mL 11C7 anti-Nogo-A antibody and incubated for 1 h at 4 °C. Cells were then fixed and processed as described above. Images were analyzed with Fiji / ImageJ.^74^

## Supplementary information

Supplementary figures (total 4) and Figure legends

Nogo-A interactome

Motif analysis

## Data Availability

RNA-sequencing data are publicly available at https://www.ncbi.nlm.nih.gov/geo/, accession number GSE160829. All mass spectrometry data are deposited on the PRIDE repository with accession number PXD030386.
