# Correction: An unexpected role of Nogo-A as regulator of tooth enamel formation

**DOI:** 10.1038/s41368-024-00330-y

**Published:** 2024-11-08

**Authors:** Pierfrancesco Pagella, Chai Foong Lai, Laurence Pirenne, Claudio Cantù, Martin E. Schwab, Thimios A. Mitsiadis

**Affiliations:** 1 Orofacial Development and Regeneration, Institute of Oral Biology, University of Zürich, Zürich, Switzerland; 2 Laboratory of Molecular Materials, Division of Biophysics and Bioengineering, Department of Physics, Chemistry, and Biology (IFM), Linköping University, Linköping, Sweden; 3 Wallenberg Centre for Molecular Medicine, Linköping University, Linköping, Sweden; 4 Department of Biomedical and Clinical Sciences, Division of Molecular Medicine and Virology; Faculty of Medicine and Health Sciences, Linköping University, Linköping, Sweden; 5 Institute for Regenerative Medicine, University of Zürich, Zürich, Switzerland

**Keywords:** Mutagenesis, Dental diseases

Correction to: *International Journal of Oral Science* 10.1038/s41368-024-00323-x, published online 20 October 2024

Following publication of the original article [1], there was an error in the article title.

The published title was:

An unexpected role of neurite outgrowth inhibitor A as regulator of tooth enamel formation

The correct title should be:

An unexpected role of Nogo-A as regulator of tooth enamel formation

The original article [1] has been updated.

## References

[1] Pagella, P., Lai, C. F. & Pirenne, L. et al. An unexpected role of neurite outgrowth inhibitor A as regulator of tooth enamel formation. *Int. J. Oral Sci.* **16**, 60, 10.1038/s41368-024-00323-x (2024). 39426966 10.1038/s41368-024-00323-x PMC11490607

