# Supplementary material for: An unexpected role of Nogo-A as regulator of tooth enamel formation
Source: Int J Oral Sci. 2024 Oct 20;16:60. doi: 10.1038/s41368-024-00323-x (PMC11490607; doi:10.1038/s41368-024-00323-x)
Supplement: Supplementary file 1 — Supplementary figures (total 4) and Figure legends [file 41368_2024_323_MOESM1_ESM.pdf]

Supplementary Figures and Figure Legends

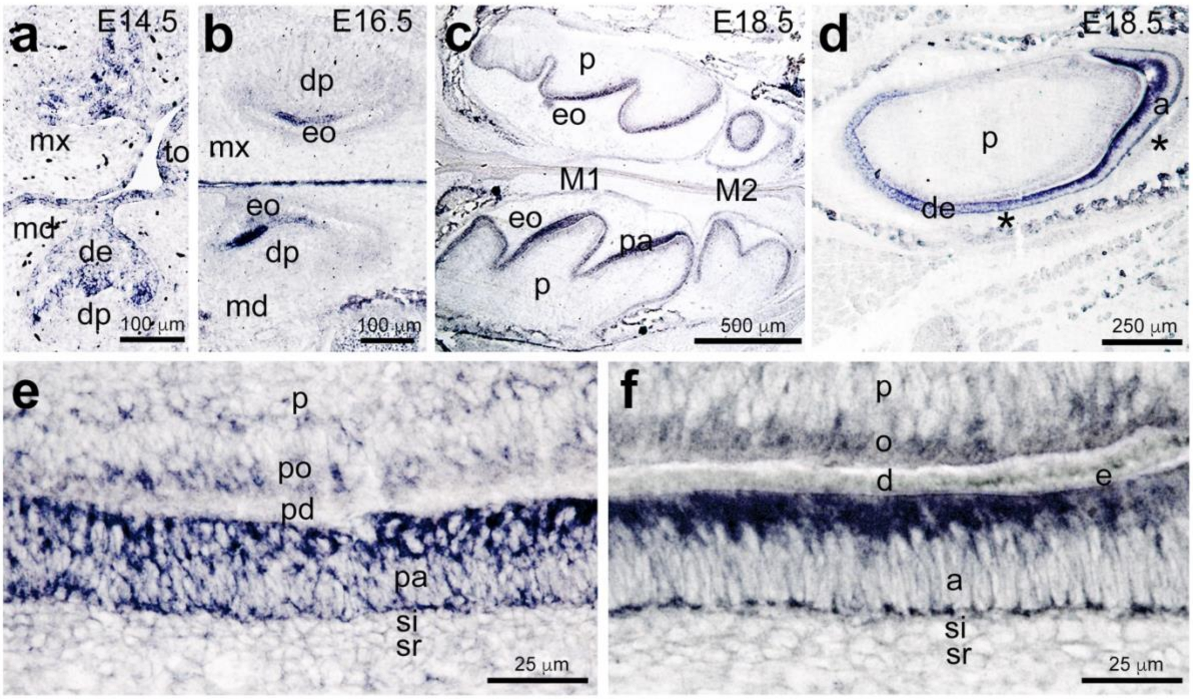

Supplementary Figure S1. *Nogo-A* mRNA expression during embryonic tooth development. a-c) *In situ* hybridization showing *Nogo-A* expression in the developing mouse molars at E14.5 (a), E16.5 (b), and E18.5 (c). **d-f)** *In situ* hybridization showing *Nogo-A* expression in lower incisors at E16.5. Asterisks in (d) indicate higher magnifications showing expression in preameloblasts (e) and ameloblasts (f), respectively. Abbreviations: a, ameloblasts; d, dentin; de, dental epithelium; dp, dental papilla; e, enamel; eo, enamel organ; M1, first molar; M2, second molar; md, mandible; mx, maxilla; o, odontoblasts; p, dental pulp; pa, preameloblast; pd, predentin; po, preodontoblasts; si, stratum intermedium; sr, stellate reticulum; to, tongue. N > 3 animals for each developmental stage.

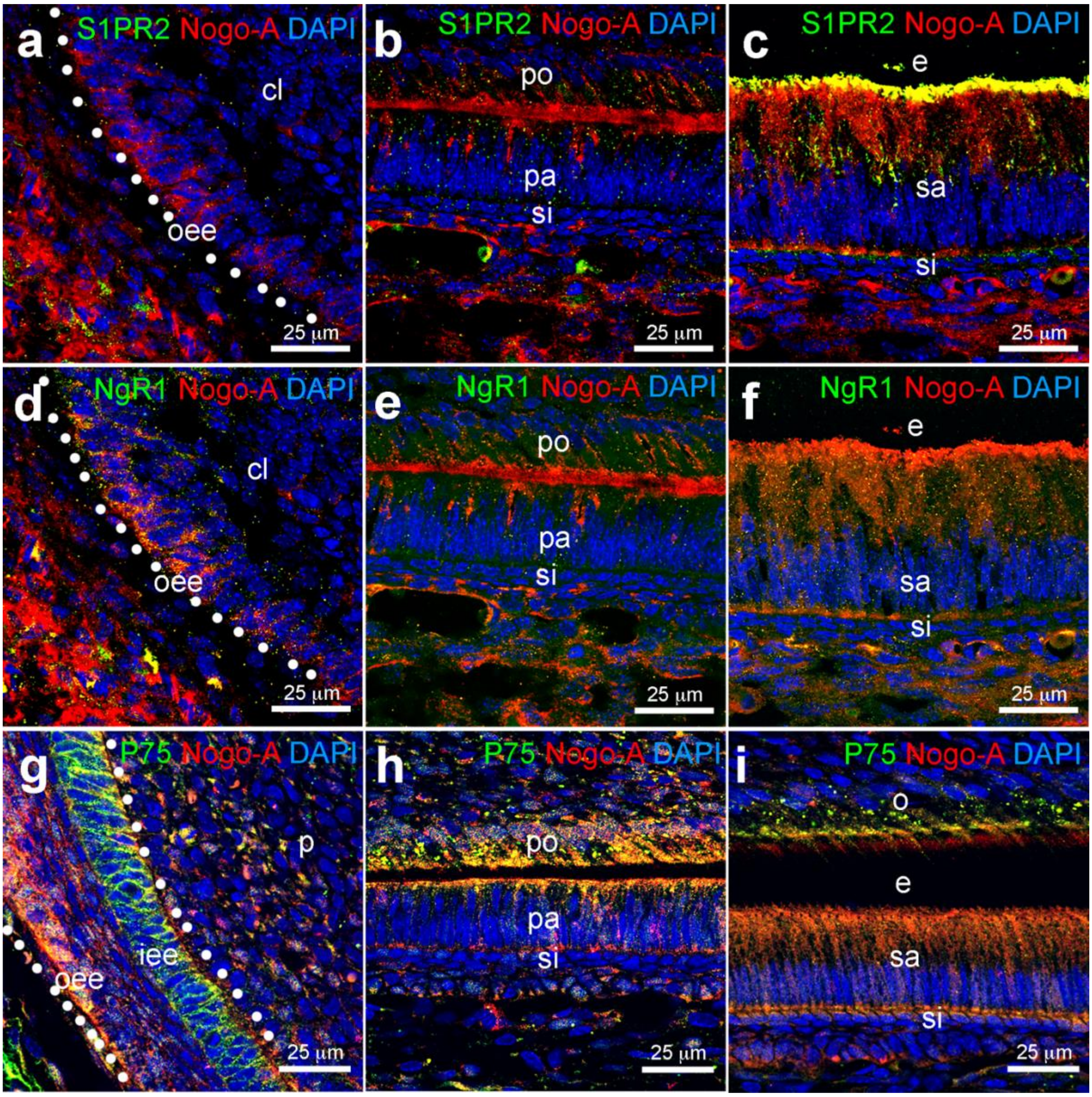

Supplementary Figure S2. Expression of Nogo-A receptors in the incisor dental epithelium. a-c) Immunofluorescent staining showing expression of S1PR2 (green colour) and Nogo-A (red colour) in the labial cervical loop region (a), in preameloblasts (b) and secretory ameloblasts (c). S1PR2 localisation in the secretory ameloblasts (c), but not in dental epithelial stem cells, transit amplifying progenitors (a), and preameloblasts (b). **d-f)** Immunofluorescent staining showing expression of NgR1 (green colour) and Nogo-A (red color) in the labial cervical loop region (d), preameloblasts (e) and secretory ameloblasts (f). NgR1 localisation in axon-like structures surrounding the cervical loop, and in the outer dental epithelium (d). a-c and d-f were performed on the same tissue slide (triple immunofluorescent staining against Nogo-A, S1PR2, and NgR1) to facilitate the comparison of their relative localization. **g-h)** Immunofluorescent staining showing expression of $P75^{NGFR}$ (green colour) and Nogo-A (red

colour) in the labial cervical loop region (g), preameloblasts (h), and secretory ameloblasts (i). $P75^{NGFR}$ localisation in cells of the inner enamel epithelium (g), and preameloblasts (h). Absence of $P75^{NGFR}$ in secretory ameloblasts (i). Abbreviations: cl, cervical loop; e, enamel; iee, inner enamel epithelium; o, odontoblasts; oee, outer enamel epithelium; pa, preameloblasts; po, preodontoblasts; sa, secretory ameloblasts. $N > 3$ animals were used for each immunostaining.

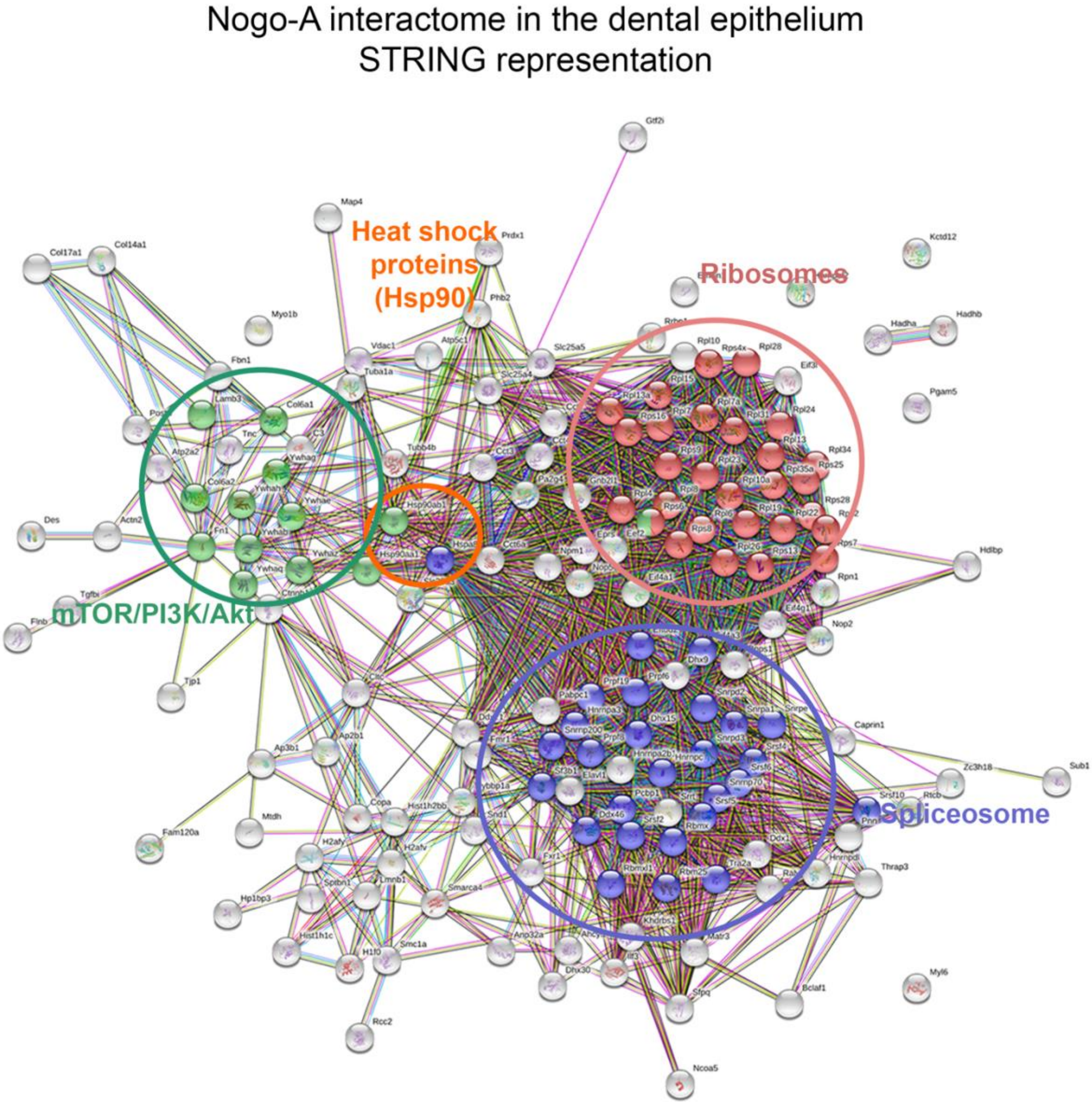

Supplementary Figure S3. Nogo-A interactome. STRING representation of Nogo-A interactors, identified in 2 independent immunoprecipitation experiments and not detected by immunoprecipitating with a control IgG.

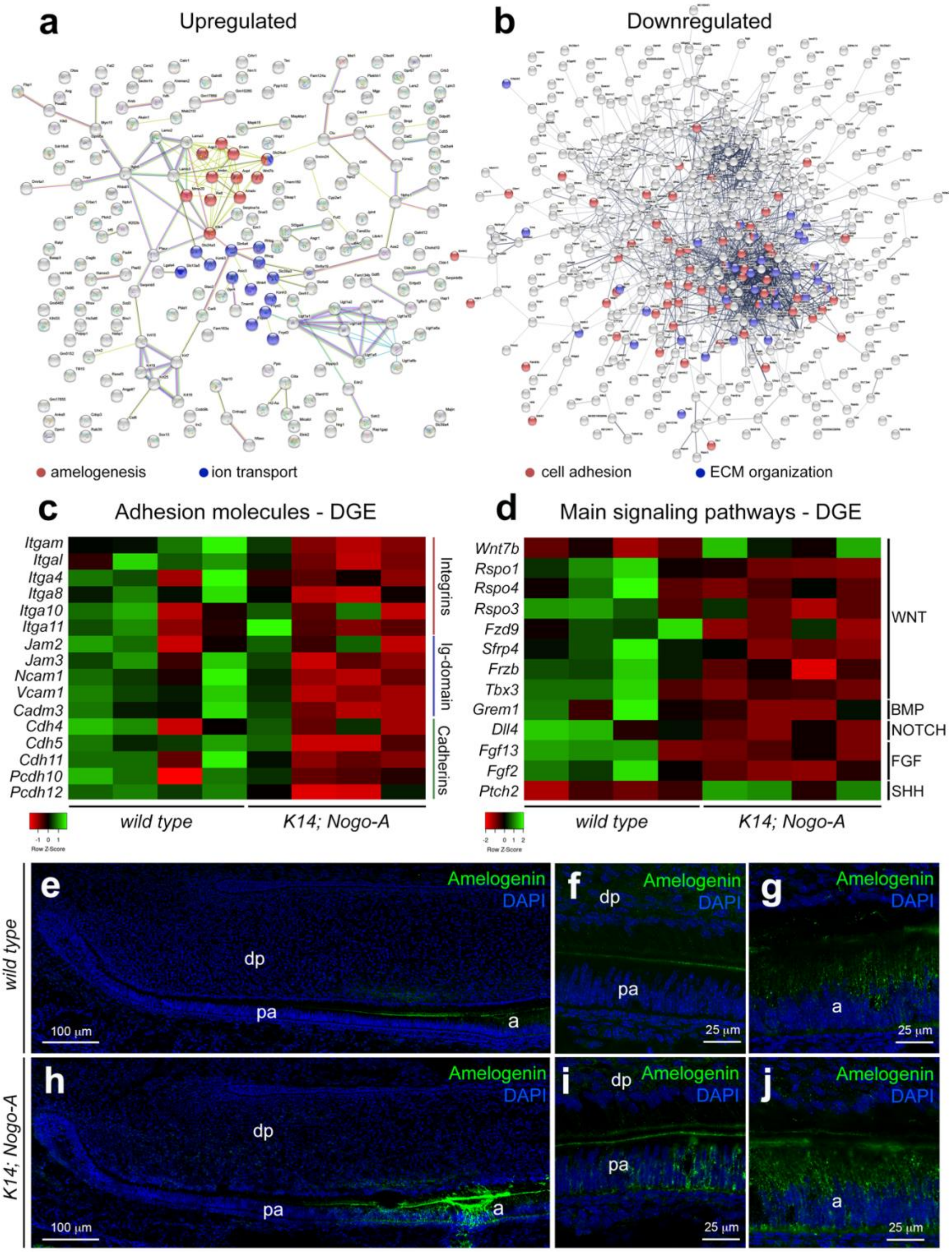

Supplementary Figure S4. Nogo-A deletion affects the expression of genes involved in ameloblast differentiation and cell adhesion. a, b) STRING representation of key networks affected by deletion of Nogo-A in the dental epithelium. **c, d)** Heatmaps showing relevant differentially expressed genes (DEGs) involved in cell adhesion and in major signalling

pathways. **e-j)** Immunofluorescent staining showing distribution of amelogenin (green color) in the lower incisor of wild-type (e-g) and K14cre;Nogo-A mutant (h-j) new-born mice. Blue color: DAPI. Abbreviations: a, ameloblasts; dp, dental pulp; pa, preameloblasts.

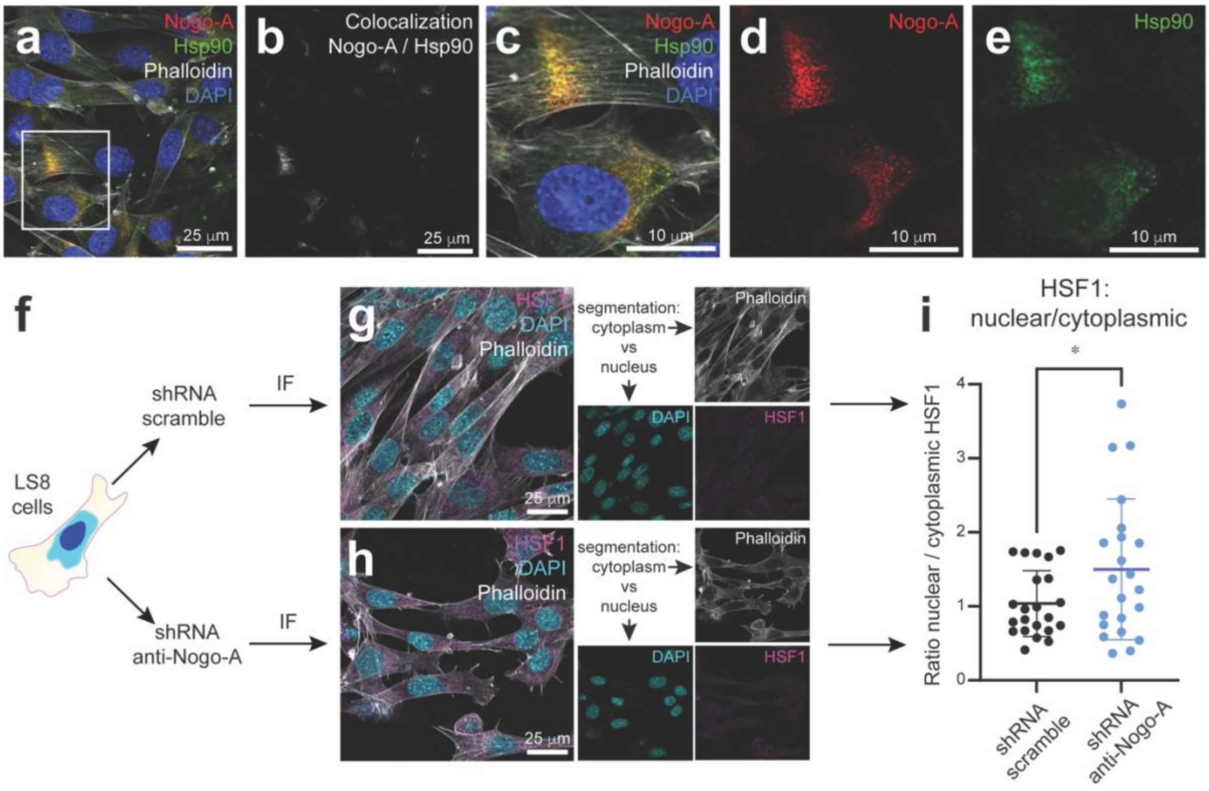

Supplementary Figure S5. Nogo-A interacts with Hsp90 in LS8 cells, and its knockdown affects the localization of the Hsp90 effector HSF1. a-d) Colocalization of Nogo-A and Hsp90 in LS8 cells. (a): Immunofluorescent staining showing an overview of Nogo-A (red color) and Hsp90 (green color) in LS8 cells. White color: phalloidin, blue color: DAPI. (b): Colocalization analysis (performed with the "Colocalization Threshold" plugin, Fiji/ImageJ [74]) showing in white sites of Nogo-A/Hsp90 colocalization. (c): higher magnification of the region marked with a white square in (a). (d, e): single channel images of (c), showing Nogo-A and Hsp90 expression, respectively. **f-i)** Quantification of HSF1 nuclear vs cytoplasmic localization in LS8 cells treated with an anti-Nogo-A shRNA and a scramble shRNA control. (f): experimental approach. (g): Immunofluorescent staining showing expression of HSF1 (magenta) in scramble shRNA-treated LS8 cells. Cyan: DAPI, white: phalloidin. DAPI and phalloidin signals were used to segment nucleus vs cytoplasm and assign the HSF1 signal to these two compartments. (h): Immunofluorescent staining showing expression of HSF1 (magenta) in anti-Nogo-A shRNA-treated LS8 cells. Cyan: DAPI, white: phalloidin. DAPI and phalloidin signals were used to segment nucleus vs cytoplasm and assign the HSF1 signal to these two compartments. (i): Quantification of nuclear / cytoplasmic HSF1 signal in LS8 cells where Nogo-A was knocked down (shRNA anti-Nogo-A) and in control LS8 cells (shRNA scramble). 16 cells per condition, from 2 independent replicates, were analysed. Statistical test: Student's T-test, 2 tails; * = $p < 0.05$.
