# Supplementary material for: An unexpected role of Nogo-A as regulator of tooth enamel formation
Source: Int J Oral Sci. 2024 Oct 20;16:60. doi: 10.1038/s41368-024-00323-x (PMC11490607; doi:10.1038/s41368-024-00323-x)
Supplement: Supplementary file 2 — Nogo-A interactome [file 41368_2024_323_MOESM2_ESM.pdf]

NogoA_interactome

Accession	Species	Protein Name	
1433B	MOUSE	14-3-3 protein beta/alpha OS=Mus musculus GN=Ywhab PE=1 SV=3	
1433E	MOUSE	14-3-3 protein epsilon OS=Mus musculus GN=Ywhae PE=1 SV=1	
1433F	MOUSE	14-3-3 protein eta OS=Mus musculus GN=Ywhah PE=1 SV=2	
1433G	MOUSE	14-3-3 protein gamma OS=Mus musculus GN=Ywhag PE=1 SV=2	
1433T	MOUSE	14-3-3 protein theta OS=Mus musculus GN=Ywhaq PE=1 SV=1	
1433Z	MOUSE	14-3-3 protein zeta/delta OS=Mus musculus GN=Ywhaz PE=1 SV=1	
ACTN2	MOUSE	Alpha-actinin-2 OS=Mus musculus GN=Actn2 PE=1 SV=2	
ADT1	MOUSE	ADP/ATP translocase 1 OS=Mus musculus GN=Slc25a4 PE=1 SV=4	
ADT2	MOUSE	ADP/ATP translocase 2 OS=Mus musculus GN=Slc25a5 PE=1 SV=3	
AN32A	MOUSE	Acidic leucine-rich nuclear phosphoprotein 32 family member A OS=Mus musculus GN=Anp32a PE=1 SV=1	
AP2B1	MOUSE	AP-2 complex subunit beta OS=Mus musculus GN=Ap2b1 PE=1 SV=1	
AP3B1	MOUSE	AP-3 complex subunit beta-1 OS=Mus musculus GN=Ap3b1 PE=1 SV=2	
AT2A2	MOUSE	Sarcoplasmic/endoplasmic reticulum calcium ATPase 2 OS=Mus musculus GN=Atp2a2 PE=1 SV=2	
ATPG	MOUSE	ATP synthase subunit gamma, mitochondrial OS=Mus musculus GN=Atp5c1 PE=1 SV=1	
BCLF1	MOUSE	Bcl-2-associated transcription factor 1 OS=Mus musculus GN=Bclaf1 PE=1 SV=2	
BGH3	MOUSE	Transforming growth factor-beta-induced protein ig-h3 OS=Mus musculus GN=Tgfbi PE=1 SV=1	
CAPR1	MOUSE	Caprin-1 OS=Mus musculus GN=Caprin1 PE=1 SV=2	
CLH1	MOUSE	Clathrin heavy chain 1 OS=Mus musculus GN=Cltc PE=1 SV=3	
CO3	MOUSE	Complement C3 OS=Mus musculus GN=C3 PE=1 SV=3	
CO6A1	MOUSE	Collagen alpha-1(VI) chain OS=Mus musculus GN=Col6a1 PE=1 SV=1	
CO6A2	MOUSE	Collagen alpha-2(VI) chain OS=Mus musculus GN=Col6a2 PE=1 SV=3	
COEA1	MOUSE	Collagen alpha-1(XIV) chain OS=Mus musculus GN=Col14a1 PE=1 SV=2	
COHA1	MOUSE	Collagen alpha-1(XVII) chain OS=Mus musculus GN=Col17a1 PE=1 SV=3	
COPA	MOUSE	Coatomer subunit alpha OS=Mus musculus GN=Copa PE=1 SV=2	
CTNB1	MOUSE	Catenin beta-1 OS=Mus musculus GN=Ctnnb1 PE=1 SV=1	
DDX1	MOUSE	ATP-dependent RNA helicase DDX1 OS=Mus musculus GN=Ddx1 PE=1 SV=1	
DDX21	MOUSE	Nucleolar RNA helicase 2 OS=Mus musculus GN=Ddx21 PE=1 SV=3	
DDX46	MOUSE	Probable ATP-dependent RNA helicase DDX46 OS=Mus musculus GN=Ddx46 PE=1 SV=2	
DESM	MOUSE	Desmin OS=Mus musculus GN=Des PE=1 SV=3	
DHX15	MOUSE	Pre-mRNA-splicing factor ATP-dependent RNA helicase DHX15 OS=Mus musculus GN=Dhx15 PE=1 SV=2	
DHX30	MOUSE	Putative ATP-dependent RNA helicase DHX30 OS=Mus musculus GN=Dhx30 PE=1 SV=1	
DHX9	MOUSE	ATP-dependent RNA helicase A OS=Mus musculus GN=Dhx9 PE=1 SV=2	
ECHA	MOUSE	Trifunctional enzyme subunit alpha	
ECHB	MOUSE	Trifunctional enzyme subunit beta	
EF2	MOUSE	Elongation factor 2 OS=Mus musculus GN=Eef2 PE=1 SV=2	
EIF3L	MOUSE	Eukaryotic translation initiation factor 3 subunit L OS=Mus musculus GN=Eif3l PE=1 SV=1	
ELAV1	MOUSE	ELAV-like protein 1 OS=Mus musculus GN=Elavl1 PE=1 SV=2	
EMIL1	MOUSE	EMILIN-1 OS=Mus musculus GN=Emilin1 PE=1 SV=1	
F120A	MOUSE	Constitutive coactivator of PPAR-gamma-like protein 1 OS=Mus musculus GN=FAM120A PE=1 SV=2	
FBN1	MOUSE	Fibrillin-1 OS=Mus musculus GN=Fbn1 PE=1 SV=2	
FINC	MOUSE	Fibronectin OS=Mus musculus GN=Fn1 PE=1 SV=4	
FLNB	MOUSE	Filamin-B OS=Mus musculus GN=Flnb PE=1 SV=3	
FMR1	MOUSE	Synaptic functional regulator FMR1 OS=Mus musculus GN=Fmr1 PE=1 SV=1	
FXR1	MOUSE	Fragile X mental retardation syndrome-related protein 1 OS=Mus musculus GN=Fxr1 PE=1 SV=2	
GTF2I	MOUSE	General transcription factor II-I OS=Mus musculus GN=Gtf2i PE=1 SV=3	
H10	MOUSE	Histone H1.0 OS=Mus musculus GN=H1f0 PE=2 SV=4	
H12	MOUSE	Histone H1.2 OS=Mus musculus GN=Hist1h1c PE=1 SV=2	
H2AV	MOUSE	Histone H2A.V OS=Mus musculus GN=H2afv PE=1 SV=3	
H2B1B	MOUSE	Histone H2B type 1-B OS=Mus musculus GN=Hist1h2bb PE=1 SV=3	
HNRDL	MOUSE	Heterogeneous nuclear ribonucleoprotein D-like OS=Mus musculus GN=Hnrnpdl PE=1 SV=1	
HNRL2	MOUSE	Heterogeneous nuclear ribonucleoprotein U-like protein 2 OS=Mus musculus GN=Hnrnpul2 PE=1 SV=2	
HNRPC	MOUSE	Heterogeneous nuclear ribonucleoproteins C1/C2 OS=Mus musculus GN=Hnrnpc PE=1 SV=1	
HP1B3	MOUSE	Heterochromatin protein 1-binding protein 3 OS=Mus musculus GN=Hp1bp3 PE=1 SV=1	
HS90A	MOUSE	Heat shock protein HSP 90-alpha OS=Mus musculus GN=Hsp90aa1 PE=1 SV=4	
HS90B	MOUSE	Heat shock protein HSP 90-beta OS=Mus musculus GN=Hsp90ab1 PE=1 SV=3	
HSP7C	MOUSE	Heat shock cognate 71 kDa protein OS=Mus musculus GN=Hspa8 PE=1 SV=1	
HVM27	MOUSE	Ig heavy chain V-III region A4 OS=Mus musculus PE=1 SV=1	
HVM36	MOUSE	Ig heavy chain V region 441 OS=Mus musculus PE=4 SV=1	
IF4A1	MOUSE	Eukaryotic initiation factor 4A-I OS=Mus musculus GN=Eif4a1 PE=1 SV=1	
IF4A3	MOUSE	Eukaryotic initiation factor 4A-III OS=Mus musculus GN=Eif4a3 PE=1 SV=3	
IF4G1	MOUSE	Eukaryotic translation initiation factor 4 gamma 1 OS=Mus musculus GN=Eif4g1 PE=1 SV=1	
ILF3	MOUSE	Interleukin enhancer-binding factor 3 OS=Mus musculus GN=Ilf3 PE=1 SV=2	
KCD12	MOUSE	BTB/POZ domain-containing protein KCTD12 OS=Mus musculus GN=Kctd12 PE=1 SV=1	
KHDR1	MOUSE	KH domain-containing	signal tran
KV5A3	MOUSE	Ig kappa chain V-V region K2 (Fragment) OS=Mus musculus PE=1 SV=1	
KV5AB	MOUSE	Ig kappa chain V-V region HP R16.7 OS=Mus musculus PE=1 SV=1	
LAMB3	MOUSE	Laminin subunit beta-3 OS=Mus musculus GN=Lamb3 PE=1 SV=2	
LMNB1	MOUSE	Lamin-B1 OS=Mus musculus GN=Lmnb1 PE=1 SV=3	
LYRIC	MOUSE	Protein LYRIC OS=Mus musculus GN=Mtdh PE=1 SV=1	
MAP4	MOUSE	Microtubule-associated protein 4 OS=Mus musculus GN=Map4 PE=1 SV=3	
MATR3	MOUSE	Matrin-3 OS=Mus musculus GN=Matr3 PE=1 SV=1	
MBB1A	MOUSE	Myb-binding protein 1A OS=Mus musculus GN=Mybbp1a PE=1 SV=2	
MPCP	MOUSE	Phosphate carrier protein	
MYL6	MOUSE	Myosin light polypeptide 6 OS=Mus musculus GN=Myl6 PE=1 SV=3	
MYO1B	MOUSE	Unconventional myosin-Ib OS=Mus musculus GN=Myo1b PE=1 SV=3	
NCOA5	MOUSE	Nuclear receptor coactivator 5 OS=Mus musculus GN=Ncoa5 PE=1 SV=1	
NOP2	MOUSE	Probable 28S rRNA (cytosine-C(5))-methyltransferase OS=Mus musculus GN=Nop2 PE=1 SV=1	
NOP56	MOUSE	Nucleolar protein 56 OS=Mus musculus GN=Nop56 PE=1 SV=2	
NPM	MOUSE	Nucleophosmin OS=Mus musculus GN=Npm1 PE=1 SV=1	
PA2G4	MOUSE	Proliferation-associated protein 2G4 OS=Mus musculus GN=Pa2g4 PE=1 SV=3	
PABP1	MOUSE	Polyadenylate-binding protein 1 OS=Mus musculus GN=Pabpc1 PE=1 SV=2	
PCBP1	MOUSE	Poly(rC)-binding protein 1 OS=Mus musculus GN=Pcbp1 PE=1 SV=1	
PGAM5	MOUSE	Serine/threonine-protein phosphatase PGAM5, mitochondrial OS=Mus musculus GN=Pgam5 PE=1 SV=1	
PGBM	MOUSE	Basement membrane-specific heparan sulfate proteoglycan core protein OS=Mus musculus GN=Hspg2 PE=1 SV=1	
PHB2	MOUSE	Prohibitin-2 OS=Mus musculus GN=Phb2 PE=1 SV=1	
PININ	MOUSE	Pinin OS=Mus musculus GN=Pnn PE=1 SV=4	
POSTN	MOUSE	Periostin OS=Mus musculus GN=Postn PE=1 SV=2	
PRDX1	MOUSE	Peroxiredoxin-1 OS=Mus musculus GN=Prdx1 PE=1 SV=1	
PRP19	MOUSE	Pre-mRNA-processing factor 19 OS=Mus musculus GN=Prpf19 PE=1 SV=1	
PRP6	MOUSE	Pre-mRNA-processing factor 6 OS=Mus musculus GN=Prpf6 PE=1 SV=1	
PRP8	MOUSE	Pre-mRNA-processing-splicing factor 8 OS=Mus musculus GN=Prpf8 PE=1 SV=2	
RACK1	MOUSE	Receptor of activated protein C kinase 1 OS=Mus musculus GN=Rack1 PE=1 SV=3	
RALY	MOUSE	RNA-binding protein Raly OS=Mus musculus GN=Raly PE=1 SV=3	
RBM25	MOUSE	RNA-binding protein 25 OS=Mus musculus GN=Rbm25 PE=1 SV=2	
RBMX	MOUSE	RNA-binding motif protein	
RCC2	MOUSE	Protein RCC2 OS=Mus musculus GN=Rcc2 PE=1 SV=1	
RL10	MOUSE	60S ribosomal protein L10 OS=Mus musculus GN=Rpl10 PE=1 SV=3	
RL10A	MOUSE	60S ribosomal protein L10a OS=Mus musculus GN=Rpl10a PE=1 SV=3	
RL13	MOUSE	60S ribosomal protein L13 OS=Mus musculus GN=Rpl13 PE=1 SV=3	
RL13A	MOUSE	60S ribosomal protein L13a OS=Mus musculus GN=Rpl13a PE=1 SV=4	
RL15	MOUSE	60S ribosomal protein L15 OS=Mus musculus GN=Rpl15 PE=2 SV=4	
RL19	MOUSE	60S ribosomal protein L19 OS=Mus musculus GN=Rpl19 PE=1 SV=1	
RL22	MOUSE	60S ribosomal protein L22 OS=Mus musculus GN=Rpl22 PE=1 SV=2	
RL23	MOUSE	60S ribosomal protein L23 OS=Mus musculus GN=Rpl23 PE=1 SV=1	
RL24	MOUSE	60S ribosomal protein L24 OS=Mus musculus GN=Rpl24 PE=1 SV=2	
RL26	MOUSE	60S ribosomal protein L26 OS=Mus musculus GN=Rpl26 PE=1 SV=1	
RL28	MOUSE	60S ribosomal protein L28 OS=Mus musculus GN=Rpl28 PE=1 SV=2	
RL31	MOUSE	60S ribosomal protein L31 OS=Mus musculus GN=Rpl31 PE=1 SV=1	
RL34	MOUSE	60S ribosomal protein L34 OS=Mus musculus GN=Rpl34 PE=1 SV=2	
RL35A	MOUSE	60S ribosomal protein L35a OS=Mus musculus GN=Rpl35a PE=1 SV=2	
RL4	MOUSE	60S ribosomal protein L4 OS=Mus musculus GN=Rpl4 PE=1 SV=3	
RL6	MOUSE	60S ribosomal protein L6 OS=Mus musculus GN=Rpl6 PE=1 SV=3	
RL7	MOUSE	60S ribosomal protein L7 OS=Mus musculus GN=Rpl7 PE=1 SV=2	
RL7A	MOUSE	60S ribosomal protein L7a OS=Mus musculus GN=Rpl7a PE=1 SV=2	
RL8	MOUSE	60S ribosomal protein L8 OS=Mus musculus GN=Rpl8 PE=1 SV=2	
RMXL1	MOUSE	RNA binding motif protein	
RNPS1	MOUSE	RNA-binding protein with serine-rich domain 1 OS=Mus musculus GN=Rnps1 PE=1 SV=1	
ROA2	MOUSE	Heterogeneous nuclear ribonucleoproteins A2/B1 OS=Mus musculus GN=Hnrnpa2b1 PE=1 SV=2	
ROA3	MOUSE	Heterogeneous nuclear ribonucleoprotein A3 OS=Mus musculus GN=Hnrnpa3 PE=1 SV=1	
RPN1	MOUSE	Dolichyl-diphosphooligosaccharide--protein glycosyltransferase subunit 1 OS=Mus musculus GN=Rpn1 PE=1 SV=1	
RRBP1	MOUSE	Ribosome-binding protein 1 OS=Mus musculus GN=Rrbp1 PE=1 SV=2	
RS13	MOUSE	40S ribosomal protein S13 OS=Mus musculus GN=Rps13 PE=1 SV=2	
RS16	MOUSE	40S ribosomal protein S16 OS=Mus musculus GN=Rps16 PE=1 SV=4	
RS2	MOUSE	40S ribosomal protein S2 OS=Mus musculus GN=Rps2 PE=1 SV=3	
RS25	MOUSE	40S ribosomal protein S25 OS=Mus musculus GN=Rps25 PE=1 SV=1	
RS28	MOUSE	40S ribosomal protein S28 OS=Mus musculus GN=Rps28 PE=1 SV=1	
RS4X	MOUSE	40S ribosomal protein S4, X isoform OS=Mus musculus GN=Rps4x PE=1 SV=2	
RS6	MOUSE	40S ribosomal protein S6 OS=Mus musculus GN=Rps6 PE=1 SV=1	
RS7	MOUSE	40S ribosomal protein S7 OS=Mus musculus GN=Rps7 PE=2 SV=1	
RS8	MOUSE	40S ribosomal protein S8 OS=Mus musculus GN=Rps8 PE=1 SV=2	
RS9	MOUSE	40S ribosomal protein S9 OS=Mus musculus GN=Rps9 PE=1 SV=3	
RTCB	MOUSE	tRNA-splicing ligase RtcB homolog OS=Mus musculus GN=Rtcb PE=1 SV=1	
RU17	MOUSE	U1 small nuclear ribonucleoprotein 70 kDa OS=Mus musculus GN=Snrnp70 PE=1 SV=2	
RU2A	MOUSE	U2 small nuclear ribonucleoprotein A' OS=Mus musculus GN=Snrpa1 PE=1 SV=2	
RUXE	MOUSE	Small nuclear ribonucleoprotein E OS=Mus musculus GN=Snrpe PE=1 SV=1	
SAHH3	MOUSE	Putative adenosylhomocysteinase 3 OS=Mus musculus GN=Ahcyl2 PE=1 SV=1	
SF3B1	MOUSE	Splicing factor 3B subunit 1 OS=Mus musculus GN=Sf3b1 PE=1 SV=1	
SFPQ	MOUSE	Splicing factor, proline- and glutamine-rich OS=Mus musculus GN=Sfpq PE=1 SV=1	
SMC1A	MOUSE	Structural maintenance of chromosomes protein 1A OS=Mus musculus GN=Smc1a PE=1 SV=4	
SMCA4	MOUSE	Transcription activator BRG1 OS=Mus musculus GN=Smarca4 PE=1 SV=1	
SMD2	MOUSE	Small nuclear ribonucleoprotein Sm D2 OS=Mus musculus GN=Snrpd2 PE=1 SV=1	
SMD3	MOUSE	Small nuclear ribonucleoprotein Sm D3 OS=Mus musculus GN=Snrpd3 PE=1 SV=1	
SND1	MOUSE	Staphylococcal nuclease domain-containing protein 1 OS=Mus musculus GN=Snd1 PE=1 SV=1	
SPTB2	MOUSE	Spectrin beta chain	
SRRT	MOUSE	Serrate RNA effector molecule homolog OS=Mus musculus GN=Srrt PE=1 SV=1	
SRS10	MOUSE	Serine/arginine-rich splicing factor 10 OS=Mus musculus GN=Srsf10 PE=1 SV=2	
SRSF2	MOUSE	Serine/arginine-rich splicing factor 2 OS=Mus musculus GN=Srsf2 PE=1 SV=4	
SRSF4	MOUSE	Serine/arginine-rich splicing factor 4 OS=Mus musculus GN=Srsf4 PE=2 SV=1	
SRSF5	MOUSE	Serine/arginine-rich splicing factor 5 OS=Mus musculus GN=Srsf5 PE=1 SV=2	
SRSF6	MOUSE	Serine/arginine-rich splicing factor 6 OS=Mus musculus GN=Srsf6 PE=1 SV=1	
SYEP	MOUSE	Bifunctional glutamate/proline--tRNA ligase OS=Mus musculus GN=Eprs PE=1 SV=4	
TBA1A	MOUSE	Tubulin alpha-1A chain OS=Mus musculus GN=Tuba1a PE=1 SV=1	
TBB4B	MOUSE	Tubulin beta-4B chain OS=Mus musculus GN=Tubb4b PE=1 SV=1	
TCP4	MOUSE	Activated RNA polymerase II transcriptional coactivator p15 OS=Mus musculus GN=Sub1 PE=1 SV=3	
TCPD	MOUSE	T-complex protein 1 subunit delta OS=Mus musculus GN=Cct4 PE=1 SV=3	
TCPG	MOUSE	T-complex protein 1 subunit gamma OS=Mus musculus GN=Cct3 PE=1 SV=1	
TCPH	MOUSE	T-complex protein 1 subunit eta OS=Mus musculus GN=Cct7 PE=1 SV=1	
TCPZ	MOUSE	T-complex protein 1 subunit zeta OS=Mus musculus GN=Cct6a PE=1 SV=3	
TENA	MOUSE	Tenascin OS=Mus musculus GN=Tnc PE=1 SV=1	
TR150	MOUSE	Thyroid hormone receptor-associated protein 3 OS=Mus musculus GN=Thrap3 PE=1 SV=1	
TRA2A	MOUSE	Transformer-2 protein homolog alpha OS=Mus musculus GN=Tra2a PE=1 SV=1	
U520	MOUSE	U5 small nuclear ribonucleoprotein 200 kDa helicase OS=Mus musculus GN=Snrnp200 PE=1 SV=1	
U5S1	MOUSE	116 kDa U5 small nuclear ribonucleoprotein component OS=Mus musculus GN=Eftud2 PE=1 SV=1	
VDAC1	MOUSE	Voltage-dependent anion-selective channel protein 1 OS=Mus musculus GN=Vdac1 PE=1 SV=3	
VIGLN	MOUSE	Vigilin OS=Mus musculus GN=Hdlbp PE=1 SV=1	
ZCH18	MOUSE	Zinc finger CCCH domain-containing protein 18 OS=Mus musculus GN=Zc3h18 PE=1 SV=1	
ZO1	MOUSE	Tight junction protein ZO-1 OS=Mus musculus GN=Tjp1 PE=1 SV=2	
