# Supplementary material for: An unexpected role of Nogo-A as regulator of tooth enamel formation
Source: Int J Oral Sci. 2024 Oct 20;16:60. doi: 10.1038/s41368-024-00323-x (PMC11490607; doi:10.1038/s41368-024-00323-x)
Supplement: Supplementary file 3 — Motif analysis [file 41368_2024_323_MOESM3_ESM.pdf]

>5 dna:chromosome chromosome:GRCm39:5:88601472:88618800:1
AGATCCCATCTCAAAAGAAAAATGTCGCCTTTAATCCCGGCACTCAGGAGGCAGAGGCAG
GCGGATTTCTGAGTTTGAGGCCAGCCTGATCGACAAAGTGAGTTCCAGGACAACCAAGGC
TACACAGAGAAACCCTGTCTCGAAAAACCAAAAAGAAAAAAAAAAGAAAGAAAGAAAGAA
AGAAAGAAAGAAAGAAAGAAAGAAAGAAAGAAAGAAAGAAAGAAAAATGTATTCAGCTTC
CTTTACCATCAAGAAAATGAAAATTAAAACTACTTTGAGATCAAAGCAATAACTGACAGT
AAGACCTGGCAGGAAGTAGTGAAAGGGAAAGCCTTGGTCACTTTTGCTAAGATTGAAAAC
TGGACAGCCACTACAGAACTCAGTGGGGAGAATCCTTGAAATCTTAAAATAGATCATAGG
ACCAGCTATACCTCGCTCTGAAATACACTCAATGACTTGATATCCTACTAAGAGATAATT
GTATTTTGCCACTTTATTCACAATAACTAGGGAATAAAAGCAGCCTATATATCCATCAAC
TGGTGAGTAAATTGTAAAAAGTGATATATTTACATAATGGAATTCTATTCAGTTGTGAAG
AAAAAATGAAACCATAAAAAATTCGCAAGTAAATGACTAGACAGGAAAACATTATATTGA
GTAAAGTAGCCTAGACTCAGAAAGACACATGGTTCACATTCTCCCTTACTTTCAACTCCT
AGATACAAATCTTTAAGTGTATAGTTTGGCATAACCATAGAAGCTGGGAACGTAGAAGGA
AACCACTGGAGAGGAGAGAGAGCTCTTAGGAAGGAGGACAGCAAGACACAGGTGCCATCA
AGTGACTAGGTTTACAGGCCTACATCAAATGTAATCACAATCCAGTTGGTTGTCCCAGTA
CAGTCATGCCACTATTGCACCGGTGAGTGCATCTTGCCTAGCAAGTCAGTATTGCAACTT
GTTGGCGTCCAGTGCTGGGTAAGATCATCAGTCCTTTGGTGGTGGTGGTGGTGGTGGTGT
TTGTTTGGTTTTGGTTTTTCTCTTTTATTCAGAGGTCTGAATATGACTTTTAGCACTACA
AAAGTTTGACAACAGAGAAGAAGATTCCTGGTCAGCTTGAAATTAATCTTTCTGTGTCCT
ACAACCAAAGTATGTGGTGATGTCTTCAGCAATTCGATCCTAGTATCTATGATGGATAAC
TAGAAATAATGGCAATAGGCTAAGATACTCGCAGCTCTCTGGAGTCTCTCATAAGAAGGT
ATCCCATGTGGAGCACTGGGGTTTTCTTCGCAACCTATGTCTTCGGGGAGCAGGATTAAG
CTTCTTAAATAACATTATTTGAAGCTATTAGGTCTGCAGTTGGGTATGAAGGTGTCTTTC
TCCCAAAATACCAGCTTCCTCAATGTTCCCTATAATTGGTGCAATCTCTACTTTCCAGAA
GAGTCTACTGAGTCATCCCTTCTTCAGAAACTGGTCGTAAATGGATATCCATGTACAAAA
TCATACCCTAGGATAGACGTAGAGGGCAGAGGTACTCTGTCTATATCCACACCGCTCTTA
TCTGCTCATGGAGCAAAGCCCAGCCATGTTGAAGTATACTTTTTAAGTGACCCAGAGAGA
CCTTATTAATCTGGCTTGACACTGATGCCTGGTAACTGAGTGTCTAAAGAGGACAGGTAG
AGTTGTGACCTTAGATTTGTTTGGTTCCAGTAGAATATTCTAAATTATTTTCTCTCCCCA
CAAAGAACATGCATCAAAGATTTTCCATCATTCTTGCCAACTTAAATTTCTTTTTTTTTT
TTTAATTGGTTAACTCCAGACAACTATCCTTAGATGAAAATACCTAGGAAACTCAGAAAG
GAAAAAAAAAAGAGTACAAAATCTTCAACATCTGGTCTACATTTTCATATTCCATAACAA
ACCCTAGAGATAATTTGGTGTTTCCCTCGAGGGTATATGTGTGTTGGTGGGGTGGGGTGT
GGTGGTGGTAGAAGATTTCTTTTACTACAATAAACATGAGGGTCAGAATCTTGGGAGTGC
TGGCATTGGGTCACTAGCTTTATCTGCAGATAAACATTTTCACAAAACAGATAATGGCTT
GTTTTCTACCGTTTACATATATTTCCCGAAGCTCACCAAAATGATTGTCTTCAAAGCCCA
TGTCCATTATCCCCTTACTGTGTTCCCTGCAATCAGCTCTCAGAATAAATACAGGGGCTG
GGTTACAATTTAAAATAATGAGAGCCTGCAGGCCCAATGTCCTCACATGCAATACAAGGG
TGTGGACTAATTGCAGGAGCACAATTTTTTTCCTGCATTTGCCTCACTTTGATTGGTGGA
TTGGCGCTTTAAATAAAGGTTTCTGATCTTTCCAGAATGAGAGATACAGAGAGAGCCCCA
GGAACAGTCCAGAAAAAAAATTAATCTTCTTTTCTTAGAACTGTTTTGATTGGCATCATC
AGGCCTGGGAGCACAGTGAATGTCAGCATCTAAGGTAAAATGTGATTTTATCATTTCCAT
GTGTTTGCTATAAATTCTCTTTTCCTGTCTCCAGATAACCTTTACAAAAAAGATTTATCT
TCATTTATCACAAGATGCTGAATAACAGCTTATTTCAACCATTAGAATATTCCAGAAAAT
ATATTACCATTAACCTAAGAAAATAAACTTATTTGTATGACTTTTTAAAACTATGTTTGT
GATTATTAGTGATAGTGCTAATTTATAAAAGTAAAGAGGATAAGCACACCTGCTGATCAG
ATCTGCTTCCATTCGAGAGTGAACTGACAAAATGCATCTCAAGATTATTCATTTTTAGGA
AGGAAAATCACTATCCAAGCAGATAAATATACTCAACTTTAGCTTGAAGGCTGAGTTTTG
ACATGGTTCTGCATTATGACCCAATCATAGGTTGTATTTCAAATTCTGAGTTTAAACAAA
GAATACAATTCTGTAAATGGTGACTGAAGGATGCATTTTATGAAGCAAAGCATAGTGTTC
AAGGAATATTCCTGGAGGTTGTTCTTAAACATTCATTCAAATTGCAGCTTCATAAAAATA
AGTACTCAGATCCATAGTTTGGCTGCTCGTCATTCCATTCTGATAGCCACTGGTTTAGCA
GCTTTTTGCTTTTGCATTTTAGCACTTTTGCATTTTTGAGTTTTTTAACCTGATTGTCTT

TGGAAGAACAGCCACACGCTCTTAGAAATATACTTTGACCACTATGAATGCAATTTTGCC
TGAGGTGATTTTACAACCCAACTGTGTTTTAGATTCCACTTTTCAAAATGAAGGGCCTGA
TCCTGTTCCTGTCCCTAGTGAAAATGAGCCTCGCCGTGCCGGTAAGTCCATCCCTTACCC
GGGATCTCGGGCTCCTGCATCTGACTACAGCAAACCCAGGGACACTCTGTCCTGACACTT
CAGGCATGCACTCACTCAGTTTGTAGCTCCCAGTGAGTCAATGGTCACAGTGGCCACATG
TCAGCCTTATCATCGAAAGGGGATAAGTAAATATAATTTTTCTACTCTAAACAACAATTT
GGAATATTTCAAAGGCAAATTTTATGTCTTATGACACTCTTTTAACAGGTAGAAGACTAC
AGCATCCTAGTTTTCATCATGTTTTATTATCAAGTCCTTGCTAGCACTATGAAATCGAAA
TGAAAAGGAAAAATATCTCTTGGGCAGGGGGAGTATATGTCTCTTTATATTAGCAACATT
ATCACATGGCTATAGACTGTAGTTCCAGTTAAAGCTAGTATTTAGCTGGCACATTTAGCT
GTTCTACTACCCAACAAGAATTTTTAAATGAAAGAAAAATGGAGGCTGGCTCTCTAACTT
GCCTTTGGATGATAACTGAGACTGGGGTCCGCTTCAGCCATCAATCATCAATCTAAAACA
TTGGCATTCTCTGAATATACAGTGATCCACACATTAGTAATTTAGAGTCACACTGAACAA
TGGGGAAATCACGTGTTTCCTAAGTGACTGCCTGTCAGATCAGGTGTTTCTGCTTTTAAT
TTTCCATAGGGAATGTATCCTCTGTCTATTCACATCCATGTAAAAAATGTTCCTCTTTAA
CTGGTGACTTGGAGCAGATAGAAATCAGACTACCTTGCCCTGTAGTTAGAGTACTCCAAA
ATGTGGAACTAGGGCACTTACCAACAAAGGTCAAGGAGGCTGTGCTCTCATAATACCCCG
AACATCTGAACATCCTCCTGTTACACACACTGTCATGCACACATGCACATAGGCACACAC
AATCAGTCCCCTTGTCTACACAACTCATCTTTTTCTCTACCAGAAGTGTTTACTGCACAT
TTTTAGTGCATTTCCTTTAACACATACCCACAATGAATTGTTTAGAAAATTTTGCACTGT
AATTAAGAAAATGGAGTACTTGCATAAAAAAATTAAGGGTTAAAAAAAAAGTACAGGGTG
TAATACCTTAGCCTCTTACTCCTGAAGGATAATGGCCCCACTGTTCCATACTTGAGGGAT
GGATGGAGAGGCAGACAGACAGGTACTTTCAGTATAAATGAAAGAAAAATTACAATACTA
ACACAACAAAAATGAGGATATCCATGAAATCAGAGAATTCTCAAAATCCAATTTTCTAGT
AGGTAGAGTAACTTATAATTGACCCAGCACATTGGAAGCTGGAAATATTGTGAATTAGAA
GCCAGTTTTGGCTACATAACAAGTTCATACCCAGCCTGAGCTAATTACCAAAACCTTATT
TAAAATAAATGAGTAATTTTTGTTTGAAATTTTAAAATTTAATTTCTCTAATTCTATTCT
TCCAGGCTCCACTCACACTCACACACTGGTATCTGTGCTTATTAACATATGAAAATTAAA
GATGTTTAGAAAGAGAAGTATTTTATAATTTTGAAAAGATATTTGTAAATATGTTTATGC
TTCACAACAGCATCCTACTAAGTACAAGAATACTTTTCATTTAAAGAAAGATCCCCACTA
TCTGATCTGAACAATTTAAATATATAATTTTATTATTTTTATTTATGCACATGTGTGTGT
ACATATGGGTATGTGCACATAAGTGTAGGTCCCCAAAGGTGTCAGAGGCATCAAATCCCC
TGAGAGCTGTCATTACAGATGGTTATGAGCCACCTGACATGGATACTAGGAACCAAACCC
AGGACCTCTGGAAGAGCAACAGGGGCTGTTAATGTACCATAAGTCCAGCTCTGATCTAAA
AAAATTAAACAGAGATCCACAAGCTTAAACTTAAAATAATCATTAAATAATAGCACATAA
GACTTGTTAAAACCTTCTGAATGGGAAGCAAGATAGGTTCTGTATATGATTTTTCTCAAT
GAATAACCTCATACCATATCCCATAACCAAAAGCACACCCATTTATAAAAAGCACAAGGA
AATTCAACTCCTTTGCTTTTACATGTGATCGTATTTATGACTATAAAAATCTTGTGTTTT
CTTCCCTTCCTTTAATTCTCACTGTGAATGTTTCCTTTTAAAGCAACATAAAGCTAAGAT
AAACATACACAAAAATTCTGATGTGAGAGAAAAATTACTAAGTATAATAATTCCTCCAGA
TAACTTTAATTCTTCATTCTATCTGAAAATCCTAGTCATCTGAGTAATAAGCCAATATGG
CAGCCTCTCCACTTGACATTTCATTCTGCCGTGACCTCCTACTCCAAACGTACACTTAGA
GAAGTTTCTCAATAAAGAACTCAAGATCATTCATATTTCTATTTAGGCATTTCCTCAACA
ACCTGGGGCTCAAGGCATGGCACCTCCTGGCATGGCTAGTTTGAGCCTTGAGGTATGTAT
CATCAGAGGCGTGTGTGTGTGTGTGTGTGTGTGTGTGTGTGATTTCTATACTAGCATGTCCC
TGATTGTAGATAAGCATGATGCTTTGTTTCTCAGTTTCCCTATTCCAGAAATCTTTGACT
ATCATTCGGAAGAAATCTAAAATTGTTGATGACGTTCACTACAACTTTATCTCATGATTC
TACTTCATTTTTAAATTCAGAGATATTTTAAGTTTTAGGACATTCATATGTGAATCAAAG
CAACTGTAACAATTAATAATAATAATAATAATAATATATATATATATATATATATTATAT
TATATTTTCTGTGTTGAAACCCATTATTCTGTGCTTGGAAACAGCTTACACTTAGTTCTG
TTGCTTATATCTTAGATGATAAAGTCTCATGACATAAACACACTTGGACACACTTTAGCT
TTCTAAAACAAAGTGCTATCAGTATCAGTAACATGACTTTATAATTGCTCCTGTACAGGT
TGCTTTTTTTTAACTGCAGATCCCATAAATAATGCATTTTTTGTTTTTGAAAACCATGGT
GAAATAGGAAGATTTTATAATGAAGAAGCTAATGGAAACACAGATAAATTTCACAGTTTT

TCACAAGGTCAGGCAAGATTTCATCTGAGTGCAATCAGTACATGTGTGGGCCAGATAGTT
ATTTCACATAGAGGCTAATTTCATTTAGGTGGTTTTTCTCTCAGCTTTTAGCTCAAATTC
AATACAAGCAACATTCTTTTCCATTTAAAAAGTAAATCTAGGCCAGGTGTTCACCTATAA
TCCCAACATCCAGGGAGACTGAAGAGAGGAGGAGGATCATGATTTAGAGACCAACATGGA
CTGTCCGGGGAGACCCATCTCAGAAGGAAGGAAAGAGGAAAGGAAGGAGGGAAGGGAGAC
AGGGAGGAAGGGAAGAAGGAAGGAAAAAGAAATTTAAGTGAAGGAAAAAGAACGGGCTGA
TAAAGCAATAGTCTTACAACTGTCCGTGTATTGTATTTGAATAGACTGTTTTCCTATGTC
TCAGTGTGACCCCAGATTAACTAGTATTTTCCCATTTAATCAGTGACAAAAGCAAGTGTA
CTGTTTTCAGACTGAACATGGCAAAGCTACATTTTACATTTCTTTCTTTCCGTAACAATT
TGTGTCTACTTTAGCTCTATTCAAGTTGCCTTACCCCAAGACTTGTATTGCTATTAAATG
TCATGAGAACTCAGTTGTGTCAAACGGACATCTAACATATGTTAGAGCATTAACCTACTT
TTTCTTATAGACAATGAGACAGTTGGGAAGCTTGCAGGGACTCAACGCACTTTCTCAGGT
AACTATACGAGATGAATGAGATCTTCATAAACACTTTTTAAAATATTTATTACAACATTG
ATAGTTTGTAGTTATGACAGCAACAGTACTATAGTTTCTAGAATTTACTTATTAATAGGT
AAATACCTAAACACTGAGAGAAGAAATGAGTGATTAAGAAAGCCTTAAACAGCCAGGCAT
GGTGCCACACACCTTTAATCTCATCACTCGGGAGGCAGAGGCAGGCGTATTTCTGAGTTC
AAGGCCAGCCTGGTCTACAAAGTGAGTTCCAGGACAGACAGGGCTATACAGAGAAACCCT
GTCTCAAAACAAAAAAAATCCTTAAGCAAAATAGTGTGATGGTCAAACATGAGCTCCACT
CCAATAACCCCAATCTTTGGGAGACTGAGACAGGAAGAATGGTTTGAGTTCGCAGTCAGC
CTGGGCTACACAGTTAATATCAAAAGACACATTTTAGTAATATTAATATGTGCTGGCAAA
TGAAAGTAGAAATAATATATCCAGTACTCGGGAGCTGATGCACAGAGAATGCAATTCAAG
GCCAAGCTGGGAACATGCTGAAGTATAAGCCTTCCTCAGAACACTAACAAAATAAGCCAT
CAAAATATATACATTGTTACTTTAAGTAAAATAAAATTACTTATTACACTAATAATTTTG
TTAAGTTGTCCCAAACAGAAATTTCTAGAATGATAAATCACCAACAAGTTTATGGTATAT
TGTGACATGCCCAGAACATAATTTAATTTTGTTGGAAGAGAGATAATTTTGTTGGAAGAG
AGAATTGGCTTACCCATTCCACACAAACCTGAATTACTTTTGCTTTTGGTTGCTATCTCA
CAATCCTGAAATGTATGTATGCTTTCTTTGTCTCTGTCAGTATTCTAGACTTGGCTTTGG
AAAAGCACTTAATAGTTTATGGTTGCACGGACTTCTCCCACCGCATAACTCTTTCCCATG
GATAGGACCAAGGGAACATGAAACCCAGCAGGTAAGCTAATTGCATCCATGTGTTCAAAC
TCAAGGTTTAAGACTTTTTCCTACTTTTGTTATTACAATAGCAGCAAACACAAATTAGTA
ATTGACCAAAAACTCCCCTAATACCAAATAAATCAAGTCCTATAACTCCTGTTACTTAGG
GAATATTTTTATTTTAAATTTGTAATTTATTTCAAATAGCCTCTGAAAGAAGAGACCTAC
AATAAAGATATCAGGAATAACAATAAACCTCAAAAATAAAAACAGACTCTAGTCTGGATA
ACTTTCATTTTAATCAAAATTTCCTAATTACCTAAAGACTAATTTCAAATGACAATAATC
ATCCTGATTGAAAACACAATGAGCCAAACAGGAACCAATATATTAATCTGTCAACTATTA
TTAATTAGATTCCTAATAAGCATATTATCCAAACTGTGGTCTGGGGGGGAGGGGGGCACG
AAAATAAGGACATGTTAGAATGGCTCAAAGGCTCATGGGATTTCCAACTGATGGGACATA
TTTCAAGGTGCTAATAGTGAAGTAGAAGTAATGGATGGCATAGAAGCTGACACATGAGCA
ATGGCTGAAGATAAAAGGCAGAGCCAAGTTAATATGAGACTTCCATACCCATTCCAGGAG
TATGCAGTTAACATATAGATAATATGCAGCCAATCACAAGTAATTGGTAAAAAACAAAAC
CCACAATAGTTTAATATTACTCTTAATTCTACATGGACTTGCATAACAGCATTATCTACA
AGGCCACAATGACCATATGAATTGTGTTATTATTCACTTCACTTGATTGCTTCCAAAATA
GATTTATGATGAAACATATAAGTATTTTAACTATCCTTTTAATAAATCACAAAATATTGT
AAGTACCCTTCATATATCAGGTGTTTATTAGTAGCAATAGTAATTTGTGAAATACCAAGG
TATAAAATCTGTCAGACAATTCTTTTTTTTTTTTGCTCGTAATTTATTTTTCTATCCACA
TTACATCCCAATTGCTGTGCCCCTCCCTACTCTGTTCCCTGTCCCACCCTTACAAATCCC
TTCCACAATTACTCCCTCCCCTTCTCAGAAAAGAGGAAGCCCCCCTTGGCAACCCTGGGA
TATCTAGTCCAAGCAAGACCAAGCACTTCCTCTCCCATTGACAAATTCATATAAGCCAGT
AAGAAAGTGATGAGATAAGATATATACAGATAGTGGTAATTACCCAGAGGGAATGAGTCA
CACCAACAAAGAGTGCAGGTGTGGAGATTTCCGCACCCCATGAAACAATGGTCCCAACAA
AGGTGGAGAGGTAAGTATGTTTAAAACTTTCTCAAATACTTTCTCTGAAGTGTCACCTCT
CCTCCTGTGTAAACTCTGCTTTAAGACTACTCCCTTCCAAACAGCAAGCCTCCAAGTACT
TTGCTGTAGAAATTCTAGGCACTGTGGTTCAATGAACCATCTCTTTCTGACATTCTTTTC
AAATTTCTCTACAGTATGAATATTCTTTGCCTGTGCATCCCCCACCTCTCCCATCACAGC

CATCCTTGCAGCCTCACCAGCCAGGACTGAAACCCTTCCTCCAGCCCACTGCTGCAACCG
GTGTCCAGGTCACACCCCAGAAGCCAGGGCCTCAGCCTCCAATGCACCCTGGACAGCTGC
CCTTGCAGGAAGGAGAGCTGATAGCACCAGATGAGCCGCAGGTGGCACCATCCGAAAACC
CACCAACACCTGAGGTAATTCTTTGGAGCCCACCTAAGTTACTTCTTTAAAATCAGATCA
GGATCACCCACTAACACAATGAAACAAAAACCAACTGGTAGGGCATACTTAAATAATCAC
ATCTCTCAGACCTAACATGCTAAAAAAATGAACACTTTATTAATATTCTTTCATGAGACA
TAAATATCTATTTCAATCTTTTGCCATCAGAGATGTCAGGTTGCAAAAGATCTGCATCTC
TAACCTTGGCTTAATGAGACCATTCCTTAGGAATAGGACACCTCAGAGAAGTCCAGGCTC
ATCAGCTGGGGGTTTTCAGTGTTGGAAAAAGTATAGTACAGTGATTGTGTCCTCTGCAGA
TAAAATACAGCTATGCAAAATTACCTTGTAATATTGTAAGGAAGAGAACAATGGACAGAA
AAGGGAAGGTGCTATGTAACTCTCAATACTATGTAGCATGCATTTAATATTCCTTCATAT
CATCCTGACTACACCCTATAAAGAAAACATTAGGAACCCTTTTAAGCCCAAGAAGGTGCC
AGGCTTGGGACACACCTTTAATCCCAGGACTTGATCACAGGCAAAGAAAAGAGGATCTTT
GAGGTCAAAGTGAGCCTGATCCACAGATCGAATTCCAGGATATCCAGAACTACACAGAGA
AACCCTGACTTAAAAAAAAATCAATAAATAAATAAACCTAAGCTTTGAGAGGCTACAAAG
TTTTCCAAGGATTACACTAGTGGCCGAGTAAAAATTGAAACCACTCAAGTATGTGCCAAT
TCCAAACCTCATTACCATTCCCTCTATAATGTAGAACAAACCCAAGATAGACATTCTCAT
CTCCCTGTCATTCACTGGAGCCATGTCAATATAAGACAACTTTAAAAGAATAGGTTATCC
CAAGACTTAGAGAATAAGACAGAGAGACATCTTAAACTGCTCTATTATAATCACAAACTA
GAGGTGTACTCAGTTTGTCTGTTTTGAGTGACAGTTTTAATATTTCCTCATGACACAGGT
ACCAATAATGGATTTTGCTGATCCACAATTTCCAACCGTAAGTACAGATTTCAATAAGGT
TCTATCCTGGAGAAAATATATATATGTGTGTGTCTATATTTCAAAGATTTTGAATGAATT
TTCTTCTTTTCTTATTTACCTATAGGTATAGAGTATTTCTGAGTTTCTTTTTCTAAATAT
TAAGTGCTAAGTTTTCACACTAATCAATCAAAAAAATGTGTTCCTGGAACTCTTAAAAGT
CCTTCAGTATGTGCATATCATGTGTGTTCTTTTGTGATTGGGTTACGGAAGTCCACATTC
TGTCAAAAGCATCAGGATGATATCCTCCAGATGCATCTATTTGCCTAAGAATTTCATGAA
TTCATTGTTTTTAATTGCCAGAGCTGTGTCTCTAGTTGCATATGTAGCAGAGGATGGCCT
GTCAGCCATCAATGGGAGGAGAGGCCCTTGGTATTGCAAAGATCATATGCCCCAGTACAG
GGGAATACTAGGGCCAGGAAGCGGGAGTGGGGGGTTGGGGAGCAGGGTGGGGGGAGGGTA
TAGGGGGCTTTGGGGATAGCATTTGAAATGTAAATGAAGAAAGTATTTAATAATAAAACA
GTCTGTCAGTGGAGGTTTGCCTGTTTTGACTGCCTTTTCTGCTTTCCACCTCTTAAAGGT
GTTCCAGATCGCCCGTTCAATATCTCGGGGACCAATGGCACACAACAAAGCATCCGCTGT
AAGTGTCTGCTTTCTACTTCCACATCTGTGTTTGAAACTACTTAAAAGGTTGATCTTGGG
GAGATTCACTTTTTTATAACTATTTTGTTCTGCTCAGTTTTACCCAGGAATGTTTTACAT
GTCTTATGGAGCAAACCAATTGGTAAGTCCACATTCTGTCAAAAGCAGCAGTTAAAAATC
AATAGCATCTTGAGGGCTAAAAACTGTAGAATCCATACCTAACACAAGATATTCCAAATA
TATAATCCACAAATACCTACAGTCATGATTTACATAGTAAAGTGGCACACTTTCCTATTT
GCATAATTCCAAACAGACACAAACACAATCACATTAAAGAAAAGGTTAATTTTAAATCTG
TTAAATTGGCTCTATTTACTCAATAATAGTCAACTCTGTACTCTGCTCTGGACACAGAGC
TATTGAGTCCTAAGAGGATACTGAGTGGCTTTTGATATTGTCTTGAGTAGATGGCTTGAG
TTCATACAGTGACGCACTGTTTCTTTTGAACAGAATGCTCCTGCCAGAATTGGCTTCATG
AGTTCAGAAGAAATGCCTGTGAGTACCACCTTCTAACACTTCTTAAAACAATGGCCAGGG
AGAACAGCTGCTGTCTACCGGAGGCAATGACTGTAAAAGAACCTAAAGGATTATTAGACT
TCAATTCCTCTATTCTAAAACTGACAGCTCAATTCTGAAGCAGTATCAAAAGATTTAAAA
CACTTCAGAGCTGAGAATGGTGGTGATGCACAGAATCGCAGCCCTCAGGATGTGGAGGCA
AGAAGATCAAGAAGAGTTTAAGGCCAGCCTTGACTTCATAAGACCCTGACTCAAAAATAA
TTGCTTTTTCATCACATTTTCTAATCATTTGTTCCTGAGTGGTGGCTATTTTTGAGCCCA
GGATTTTTGTTCCACTCCTCGTATATGAACAGAAGAGTCACCCTTCCAAGAGGAGTCAGT
AGAGTGAACCACAAATCCTTCAAAGTGAAAAAGATGTGTGGTTCATACAGATATCTCTTG
CCTTTTAGGACTATTTAGCTCCCCCCCCCCCCTGCCCTCATGTACCTATAGGATTCTAGA
ACTTAATGTATTTCTTTTGCTGCCATGACCATGGGAGGCATAATCTAGGACTATTTATAC
TTGAGCAAAGCTGTGTGATTTTGTCATTGTTTTGACTTATTTTATTTTTTTTTGGGGGGG
GGGAAAGGTTTTATTTTGTTTATGCTTCCAGATCACTGTTTATCAATGAAGGAAAGGAAA
GGAAAGGAAAGGAAAGGAAAGGAAAGGAAAGGAAAGGAAAGGAAAGGAAAAAGAAAAGAA

AAAGGAAAGAAAAAAGGAAAGGAAAAAGGAAAGGAAAAAGGAAAGGAAAAGGAAAGGGAA
AGGGAAAGGGAAAGGGAAGGGAAGGGAAGGGAAGGGAAGGGAAGGGAAGGGAAGGGAAGG
GAAGGAAGAAGAGAAGGGGGAAAGGAAAATGAGGAAGAGGAGACAGGGGGACATAAAAGA
TAGATGGAGGGAAAGAGTTAAAACTCATACATAGAGCTATATTTGAGGGATACTGAAAAC
ATGTCCCCAGACATTCCTCCTCAACCAAGGCATAACGAAGCTTGTGTTTTGTTTCATGAT
GATAGAGCCCTTTTCAACTGCTTACTGTTTTCTTTTTTTTTTTTTAATTTTTTATTAGGT
ATTTTCCTCATTTACATTTCCAATGCTATCCCAAAAGTCCCCCATACCCACCCTCCCACC
CCCCTACCCACCCACTCCCCTTTTTGGCCCTGGCGTTCCCCTGTACTGGGGCATATAAAG
TTTGCAAGTCCAATGGGCCTCTCTTTCCAGTGATGGCCGACTAGGCCATCTTTTGATACA
TATGCAGCTAGAGTCAAGAGCTCCGGGGTACTGGTTAGTTCATAATGTTGTTCCACCTAT
CAGGTTACAGATCCCTTTAGCTCCTTGGGTAATTTCTCTAGCTCCTCCATTGGGGGCCCT
GTGATCCATCCAATAGCTGACTGTGAACATCCACTTCTGTGGCAGAGTAGTAGTAGAGAC
CTGCCTTTCCTGAAAGAGGCTTTCTCCTGCCCAGATAAGTGGAGAATCCTCTCCCCTATA
CTTCAAAGCCAGTTCTATAAGAAGTGTGTTTCGACAAAGCAGTCAGCCAACTCGCTGTTG
TGTTAGTATTTATAGCTTCTCAATGAATATAATCATGTACAATTGTATGGAATGAATAAC
ACACAGTAATTGTGTCACTGATGGACGTTTGTTTTCCAGGGAGAAAGAGGAAGTCCCATG
GCCTATGGAACTCTGTTCCCAAGATTTGGAGGCTTCAGGCAAACCCTTAGGAGACTGAAT
CAGAATTCACCCAAGGGAGGAGACTTTACTGTGGAAGTAGATTCCCCAGTATCTGTTACC
AAAGGCCCTGAAAAAGGAGAAGGTCCAGAAGGCTCTCCACTGCAAGAGGCCAACCCAGGC
AAACGGGAAAACCCCGCTCTCCTTTCACAAATGGCACCTGGGGCCCATGCAGGACTTCTT
GCTTTCCCCAATGACCACATCCCCAGTATGGCAAGGGGTCCTGCAGGGCAAAGACTCCTT
GGAGTCACCCCTGCAGCTGCAGACCCACTGATCACCCCTGAATTAGCAGAAGTTTATGAA
ACCTATGGTGCTGATGTTACCACACCCCTGGGTGATGGAGAAGCAACCATGGATATCACC
ATGTCCCCAGACACTCAGCAGCCACTGCTACCTGGAAACAAAGTGCACCAGCCCCAGGTG
CACAACGCATGGCGTTTCCAAGAGCCCTGATAACCTTGACATAGCAGCTACTTTGTGTAT
GCACAAGCTTTCCAGCTTTGTCCCCATAGTACCTTGTTGCTAAAACACTTACTATCCTTC
CGCAGTGAAGGTGTTAAGAGCACTAAGCATATATTAATAAATACAAGTGGCTAGAAATAG
TGTAGGTCCCTTCTTGCTTCCATTCTTATCAAAATAAAATGTATCAACTGTCTCCGTGAC
TTAGAAATACTATCAATGATATCAGAGCAAGTCTAAGTGTCAGCACTTGGTGGTATAGCA
TGTAGCTGTCTTAGGCATTATAAAATTTCTCTTACTACATGACATTATTATGCCCAGGAA
ATGTGACACTGCTTCTTTCTCTACACAAAAGCACTTAGTTTCAGAATTCCAAAGTATTTC
ATTTAAACCTTATTAAATGGTGATTGGTGGAAAACCCTGACTGCTATTACTGGTTTCATA
TATTGGATTTAAAATTCTTATTTATAGAATATTTTATTTAATCTAGGAAAAGAAAGGGCA
ATTGGCCTGTTTTAAATAAAGAATTTTTCTCACTGAAAATGTCAGGAATTGTATGCTTAT
TATTTATATGTATTTAAATAGTAAAGAAAAGCATACTCATCCCTTGTTTGCCACTCATTA
ACTTATTTATTTTATCCAAAGTTCTCTGAAGATTTACATAAGTCCTAAGCACTATGCAAG
GTATAAGGTTTATGGTCTCGTCCTTCAAAAATTAAAATGGACACAACTAATAGGTATCAC
AGTAGTAACAAAACAGATGGTCTAAAGCAAGCTCAGTTACTGTACATACATTATCTCACA
CTACTGCAAAAGATAAAAGTGCACTCAAGCCTACTATACATTACCAAAGTTCTATGACAA
GATCGGGTCCTGGTACCACGGAGAAGATGCTTCTGAGGGCCAGCCCGATGGCTCAGCAAG
CAAAGACACATATCACCAGGCCTGATAACATGAGTTTGATCCCCAGGGACTCACATAGTG
GAAAAAAAGAACTGACTCCCGAAAGCATCCTCTGACAGCCACATGTGTGTCATGGCACAT
GTACACACATGCACACAAAATAAATGTTAGTTTTTAAATTAGAGAGAGTAAGGCTTTTTG
TCCAACCTAAGTGACATTAGAGGAGCAATGAAGGACCAGACATGTTTTCTTAGTAACATA
AAGGTTTGCAGAATGAGGCCTCCAAGAGTAAGAAACTCAAGAGTCATTTGTATAGCAGCA
ACACAGGAAGTGGGATGTAGGAAACCACACATGCTTACTACCAGGGACTAGCTGTGTGCA
CATCAGAGCAGTCTGCCTAAAATAAGAGATTGTGAGCCCAGTTTTAGATACCATTGAAAA
TGAGCCTCCTGCTCCTCTCTTTCCACTTTTACTAAGTTTTCTAACCCAGGTTTTGAACTC
TATAAAATTAAATTCTACCCTATCCCCTGATCAGAATATGATCAAAGAAAAAAGAGCCTG
AGATGTTTCCCAACCTAGAACACTTCAGGAATGGTAGGGAGCATGTTGCAACAATTTCCT
GCCACTTCCTCTTCAGGCAGGGCTCTGTCCTCCCAAGCTTCATATACTGGGGGGGGAAGA
TGCATAAGATAAAAATTAAGTTTACCTTCTCAGTGACTGGTAAAGGTACAAGGATGGAGA
ATGATTCCATGGGGACTAGCAGACACTTGCTGAGACTTACCACTGCAGACAATGCCTCTA
AAAGAAGCAGGAAAAGACATGTAATTGCATTTGGATTCTATTCTTGACATCATATCTTAA

AATTTGTAAAACACTGGATATGAAATATAATCTCTTGATTCCTGTAACAGTGCTGCTCCA
CCCCAGGCTTAATGTTGGGATCGAGCTAAACACACAGATAAATTGCTGAGTCACACATCC
TTTTCGTCTTCCCCAACTGACCCATATGATAGAAAGAAGTCAGCACAACAGCAGAATGTA
GCATCTGGAGGATAGAATTGTTCTCGTTATCTGATGCCATTTTGCTTTTCCATCCACACT
GAACACATAGTCCCCTGCTCCTTCCTTGTAATTCATAGCACTTGGGAGACCACATGTACT
GTAAACATCAGATCATGGGTTCAAAGTTGGCTTCTGCTATTAGTATGCTGGGTACTTAAT
AGTGGTAATATCTAAATCAATTTTTGTGAGAAAAATATGTTGTTGGGTCCTTGAATGTCC
TTTATCACTTCCACGTGGTCATTTCCAACACTGACTTGGTACAAACAAGATACCCATGGA
AAGAGGCCAAATATCACTTGAAGGACGGCCATATTAACTATTGAGTTTCTTTATCGTGTT
TTTTTCCTACTAGGCATTTATGTGAGAAAAAGATGACTTCAAACTATATCCCACCCTGAC
AAGTTTACCTACCAACCTTTCTTCTAGACTCACCTCACCACTAATCTTTTGATTTTATTC
TTTTCAAATTCCTGTCCAGGAGACAAATCTGGAATTTCCTGGATAAATATAATCCTGACT
CTCAGATCATCTCCCTTGCCACACAAAATAGACGATCAGACACATCATTTAAATGACTAC
AGTTCCTGACAACTTCCAGCAGACACCAAAACACGACACAGAAATCCATGAACACACTGT
CCTCTACATAAGCTGCTCACAGGCAGAGCAGCCACACATCTGATTCCTCTGAGGTGGGAT
ACATTTCACAGTGATCACTGATGATTACATGAGACAGCAGGGGTAAACTAGCACTGCCTA
AAGCGAACCTGGACATATGGCATGGCACTCCTTATGATGACAGACCTATAAGTGGATGGA
AAGCTCTCAAACTTAAAAAAATAAAAATAAAAAAAGTGCGGGGAGCAAGAATGTGGAAAC
CTGAGTCTGTTGCTTCTGCAATTTGTGTCCTCTAGACCTGCTGCCATATTCTCATTTTCA
CTGTTGTTGTATCCTGCTTTACAGTTCAATGGGTACATACACAACATAG

Ambn_FIMO

motif_id	motif_alt_id	sequence_name	start	stop	strand	score	p-value	q-value	matched_sequence
2	HTTCTRGAAVNTTCKYM	5	2622	2638	-	14.3697	1.83E-06	0.0606	TTTCTGGAATATTCTAA
2	HTTCTRGAAVNTTCKYM	5	7815	7831	-	13.5515	3.74E-06	0.0619	ATTCTAGAAATTTCTGT
2	HTTCTRGAAVNTTCKYM	5	7235	7251	+	13.1455	5.65E-06	0.0623	TTTCTAGAATTTACTTA
2	HTTCTRGAAVNTTCKYM	5	5695	5711	+	12.1576	2E-05	0.166	CTTAGAGAAGTTTCTCA
1	DGAANDDDCN	5	11530	11539	-	9.69697	2.65E-05	0.496	TGAACGGGCG
2	HTTCTRGAAVNTTCKYM	5	2362	2378	-	8.84848	3.66E-05	0.243	ATTCTGGAAAGATCAGA
1	DGAANDDDCN	5	6769	6778	+	9.44242	7.72E-05	0.496	AGAACGGGCT
1	DGAANDDDCN	5	10264	10273	+	9.44242	7.72E-05	0.496	GGAAGGTGCT
1	DGAANDDDCN	5	15900	15909	-	9.44242	7.72E-05	0.496	GGAAGTGGCA
1	DGAANDDDCN	5	16396	16405	-	9.44242	7.72E-05	0.496	GGAAGGAGCA
2	HTTCTRGAAVNTTCKYM	5	1432	1448	+	7.90303	8.13E-05	0.449	TTTCCAGAAGAGTCTAC
**# FIMO (Find Individual Motif Occurrences): Version 5.5.6 compiled on Aug 4 2024 at 17:26:56**									
**# The format of this file is described at https://meme-suite.org/meme/doc/fimo-output-format.html#tsv_results.**									
**# fimo --oc . --verbosity 1 --bgfile --nrdb-- --thresh 1.0E-4 motifs.meme Ambn.txt**									

>X dna:chromosome chromosome:GRCm39:X:167957484:167971609:1
GACTGGCCTTGCATAAGTTTACAGTTTATCCACTGGAATCGAGAAAGATGGGGGCCTATG
CATAACTCAAGATACTCCTGAAAATGAAATTAACATCCTTTCTACTTGTCTCCATGTACC
ATGTATCAGGAGAGCATTCATCCTTGTGCATCAGCCCTCTCATCAATGGGAACTTTAATT
CTCAGCAGAGATCTTTCACAAGATGTGTTGTGAATACTTCTATATCAAACAAATACCATG
GTTTCCCCATTCAATGATAAGCTTGGGGTCCACATAGAAAAATAATCAGAAATTGATCAC
TATGTACACATGATGAATTTATACCATACCCTAGGGTGGGGAATCGAAAGTGAGGTTGGC
ATTGGGGGTAGACAAAACAGCATCTGGAAGATCAGATGTGGTGGAGATCATGAAAGGTCA
CAGTGAGTAAGGAAAATGTATAAATATAAATATATTCATATAAATGTGCACACATTTGCA
CATGCACATACACATGCTACATATACAGACACATTCATGCACATACACACACACACACAC
ACACACACACACACACACCCCTACATTAATTGCATCCAGCAGGGATTGAATTTAGTGGAC
ATTATGGCTGTTAGCTTTCTGTTTTTATGAGACTTCTCTAATAGTGAGATGCAGTGCGCT
GCTGACTCTTTTGCCTGCTCTTAGGATTCTTTTCCTCTACTCGGTTGCCTTGTCTAGCCT
CAATATGAGGGATTTCACCTTGTCTCGTTGCATTTTATTTAGTTATGTTTAGTTGTTGTT
TCTTGAAGGCCTGCTCCTTTCTAAAGGGAAACAGAAGGGAAGGAGATCTGGAGGAGAGGG
GAGGTTGCATGAGCTAGGAGAAGTGGAGTGAGGGGAAACTGTGCTCAGGATGTATTGTAT
GAGGGAAGATTCTATCCCCCTCCACCAAAAAAAAAAGACTGAAAGAAAGATATGAGCACA
TTTGATAGGTGAAGAAATTGAGACACAGGGACAAGAGATTTATCCAACTTTCCACAGCTA
TGGTCATAATTATGACTCAGAAGCTCTGCTTGTAAAGTCACTCCACTGGAAACCATCATT
GAATTTGGGTGGACACTTGAATAGCCCCAGCGTACTCACATTCCACACCCAAATACAGGA
TAATGGATTAGGATTGTGGATGTTTTATGGTGTTGAAGTAATTTGATATTGTTTGAGGTA
TTTTTAACTCATAAGAAAAAGATACATATTTTAAACCCCACAGTGGTTTCTTCCCTCTCT
TCATTCCCTCTTTCCTTTCACTTTTCCATTAAAGGACAGGGACATTGGTAGGAGTGCTGA
GTACTGAGTTTGGTTTTGTAAACAAAGGAGGTGGATATTATTTAGCTTTTACCCTTTCTC
TCCTATAAGCCTCAATTGTTATTTATCTTTTGTAAGTGTCAGGTGTTTTACACGAATCTC
ATGTGGACTCTTCACAGGAAGCTTTTTGAATAACTAATTTCAGCCAATAACCCTTATTGA
TAAGGTTACAGTTTGTTTTTTTTGTTGTTGTTGTTTTTGTTTTGTTTTGTTTTTGAACAG
AGATTGGTTAGAAACTAAATCTCCCCAAAAGAAATCCTCCAAGTAATAAGAAATCCACAA
CGAATGTAAGCCGATGAGAATTTTATTAAGGCAATATTGAACACTACTACATGCCATTAT
GTTCTGGGAAGGTAGGAGAGTGGTTGGAATCATTTTTCTGTTGTGCTTTCCATGTTAAGC
TGTTAAGATAAGTAGAACATCATATTACTTTTATAATCAACACAATTATTTAGTATTTCA
CATACCTTTTAAAGTTTTGTATTTGCATTCAAACTTGGTGTTTCCTGCTATTGTGTCCTA
CCAAGGGGCAGACATCAGGTTTATGGTGAAAGTCTATAGATACATTTTGTATGGAGTTAC
AAAAGTAGATATAAACATGTTTCATGGGGAAAAATGAGCAACTTGATTTATTTCCCAAAA
TTTTATTAAACTGTAATATGTCTTGTTAGTAATAGATAATGTCATCCCTATATATTCTCT
AAGTGGTTGGAATGAGGAAAAGACATTTAAAATTTCAAGTTGCTCAAATATTACCTGTCT
CCACACATGTGCTTTTTTTATAGGTAAACCCAATGGACATGTGTATGTGCAAACATGTGT
GTGCCCTAATAATGCTGTAATTTTTGAACAATGAAACAAAAGCGAATGCAAAATTTTCTA
GCAGTTACAGCCAATAAATCTATGTCAGAATTGAGTAAAAAAAATGTGGTGGTTTCAGGT
TTTTTTAATCAGCTTAAACTTAACCTCTTGCCAGTAAAGCTGCAGGTCAAGGTCAGACCT
AACATACCTCCAGTCCAGATGTGGCTGGTTCAAAGTCTGAAACACCATTCATTTGGTGCT
AAACAATTCTATTACAAACAGGGAAATGAATTTATTTCAATTCCAGATTTGCCTGTTTTG
GAAGGGAACTAGAGGAGAAACCATCATAGTGTATATGGTTTGACAAAAGAAAGCAAAAGA
ATACTCTAGTTCTTAGGAAAATCCTCTTTCATGACAATGATTACTGACATGCTTAGGAAA
TAACATCTTATGGAGAACTAAAAAGTTTCACATTTAACCAATCTGTTAATCTGTTTTGTG
TCATCTTTGGTTTCAAATCTTGGAATTAATAAATATGCTTTGTCTGACTCAGAAAGTAGT
GTATAGAGAAACAGTGATAAAACTCTCAGTACTATTAGCAGTGTTGTCCAATGTGGCAGT
TATTTGCCTAGTGTGGGTCCTGAGCATTTAGAATATATCAACTATAGTAAAAAATCTAAG
TTATACATTTTATTCCTTTTTAGTTCATTAAGATATAAATGCCAACACATGACTAATGTG
TTGGACATCACTGGTATAAAACCTAATTACCACTTAATATATAGCTCTGGTTTAAACATT
CTATTGCCTTTTGATCCTATGTATCTACATTTTGAGTATAGTAGTATGGCAACCACTTTG
ATAAATGATAGTTACTTCCAGAATTTTTAAAAATATCCATCCTCAATTTCTGCTAAAAAG
ATGAATATTTATAGAAATATCAGTTGAAATTAGATTATTAGGTTATATCTTCCATTTTAT
TTTCTAATATAAGAGCTATGCACAATTCTATCAATTAGAATCATTTATAGAAGTTGAACT

CTTCAGTATGGTACAAACCCAATATTATGAAGCATACAAAAAAGTACATAGAAGCCCAAA
AGTAAAGGTTGGAAGGTTAATGTGAGCAATATTTTAGGCATTTATTGAAGTTGGATGTTC
ATTAATATCAAAAGTTATTGTAAACCCTCAAGTTTTGCAGGTTTACCACATAGATATTAT
AATATGCCTGTGAATTCTATGGCCTTTGAAACCATTTCACCTCCAACAGAATATAAGTAG
TGTTAGTCTTAAGTGTATACATTGGAATAAACATATTTGAATTAATACGGATATTTGAAA
TGCATGCTTGTTAACTTAAAGTAATAAAAAGCAAATTGTCTACATACCGGATGCCTTGTC
ATGCCCTGGTACCACTTCATAGGAGAAAACGCCTTAAGAGAAAGAGACATTAGAATGCAG
TTGATTTAGTTTTGTTTCATATGAACTGAATGCTAAACTCACTAATGCAGTATGAAATAT
AGACTCACTAATGCAGTCCTGTCAGTATTGATAGCCTGAGAATGTGACTTCTACAAAAGA
GAGCAGCTAAACACAAGTCCAAGGCTCCTATGTCAAGTATTGTGTCACTAATATTGCAGC
ACATTCCATAAGTTGTCCATGATCTGAGGCAAGCACAAATACCCTCATCTTTTAGAAAGA
GTCATGAAGAGTTAGAGTCACATTATGCAACAGGGCTATAAAAATGGGAATTCTTTTTAT
GCTCCACAGTTCATGCTTCTCATTGAAATCGGAAAGCATGGAGCTCTAAGTGGTCACAGG
GATGGGGAATTTCAGGACACAATGATTCCACCAAGATCCCCACGAGTTTGTATTTTTCTA
CTTCCAGCTTTTGATCACAAGACAGCAGACACAGCTTCATGAATCACAATTCAAACAAAT
GATCAAAAGAACATCAGTTTTTTTTTTCTCTAGGGTATATCCATACATCCTTTAAGAAAA
GGGAAGACTAATCCCTAAGATTTTTTTTTCCCCAGGAAACTTGTCCTTTGCCCTCATTCT
ATATTCATGGTCGACCACAACCTATCCGTCAACTAATAACAGAAAAGAAGTATGGAAACT
GATGTCATAGTTAGTGTGGAAGAATGGACCTCCACTCCCTGGGTAGTGGAGAGGGCACAT
CTCAATCCCAAGTAGGTGAAAGGCCACATGCTGGGATCAGTTCCAGCTCTACCAGTGAGG
AATAAGTTGTGAGCAAGCTGATGAACAGCTATTAATAGTGACTGCTATGTGCCATAGAAT
TTTGACTTTACTTATGCCATAGGATTAATGCCATTTGAACATCCTTTCCTTTTGATGGCT
AGGTTATTGTATCCCTTTGGTGGATTCTAGAACCCTAAGGATAAGGAATTACTGTTTTCA
CTATCACATACAGTAATACCCAGTCTAGTGAGTGCTTGAGAAATATTTACTCAGTTGATT
AGAAGTGGCCTATGCTAATTCATTAAATGCTTTATTTTTACCTTTATTACTGCATTTTAG
TTCTTTAAAATCAACTAATTTGTTTGCATATTGATATCTTCTCAGAGAAAGCTATGTTTA
TTCATTCCAGAAAATGATTAAGAGAGACCTTAATCATGAAGGTACTGGAATAAAGTTACT
GTGGGTGCTGTGTAACATAAATCCTGCAAAGACTGTTTACAATTTTGTGTGGGTGTAAGA
GGAAAAATGATTCAAATATAAATATGAGTGGGAAAGAAAACTTGTTTTGAGTCCATTTTA
CGCATCTGTTAGCGTTTTGAAGACCATGTGGTAGACTTAATCAGCTTAAAGATTATGAGG
TGAAAGAAATGACTTGTGTATAAAATCACTGCAATTCTCTGTTTGAGGCTCCGTTATTAA
TTTTCTTAGGAAAAATAATTATTTTAATAATTTAAATTTCAGTGAATTACCATATATATC
ACATAATTTCACTCAAAATATTTTTCTATAGAATGAGATCAAAAGACCAGATTTTGGTTA
CTGATGTGGTGTGAAGAATAAAGCTAAAGTTCCAAGTCTTAATTTGATTTAGAATATAAA
CATTCCTTAAAGTTTATCTTTTTCAATGTAAAGAATATTTCTTCTAAACTTGCCTTCATT
TGGTTCAGGTTCAGTGACTATCCATGCTTAACTATTTCTGTTAACTTTCTCAGCTATTGT
TTATGATGTACAAATTTCTATTTTAAGAAAACATTACAGATAATATAAAAGTTCTCTAAG
AAGATATAGTCATGATTTGCCTGCTAAATGGTCAATTTGTAGGAGCAAATGACCAGTCCC
TATATTATTCACTTATAAGTTCTTCTCTCAAGTGATAGGAAAATTTAAATTTAATTCAAC
ACAAAGACATGAATGATTTTTCAAACATTTTATTATCTGCTAATGATACTTATTTTTAGT
TACAGAATTTACTAAGTGATTGTAGGCACAAATCATTGTGTTCTTGAGTTATTCCTAAAA
ACAACCAAAGTATCCACTTCGGTTCTCTCATTTTCTGAATTTTTTAATCCTGAAAAAATA
TAGCAAAAGGAACAATTAACATAATTTACAAACATTCCTAGTGAATACCATTTACAATAA
TAAGTTATGGCTTCAGTTTTTTCAAAACAATGTATAACCATTAACTTGATTTTGTAATTT
CTGAAGTTTCTTGTCTTGTCATTAAATAGAAGCTCTCTCTGTTGAAATCACTAAGGCATA
TTAATAATATATTAGTAGCTGCCACCTTGCATCAATTGTTGCCTGCTATAATGTCATTAC
TTTATTTTATTTTCCATAGGGCAACTCTAGGCAATCATCAATAATTTTACTCTAATTTTA
AACAGGAGGATACTGAGTTTAAAATGATCAAACTACCTACCCAAATTCACTTTTCCACAA
ATATGTTTGGCAAGATAAAACCCATGATCAAATCTACAGTGTTAATCGTTAGTAAAGACA
CAAATAAACCCACTTTGCATGCAAACATAGAGCATGTACCTTAGTCTAGGATAATCTGTA
CTAGCTACCAAGTCATTCAATTCTAGTCAATTCTCCAAAGCTTTGCCTCATTTCCATAAA
CAGGCTTACAATTGTGAAGACATTCATGACAGTGTATAAGGAAATGCTGGCATTAATTCT
AAGTTTTAGAAGTTATCTGTTTTTCCCCCTTTTCTTACCTTAAAGAGGGAAAACACCCTC
AAGATGGAAGTAACATGGCACCTTACCATCTCTTTTTTTTTTTTTTTTTTTTTTTTTGAG

GGAAAAGCTAGGGCTAGAACCTAGATGCCCTGGTTCTAAGGACAAGCTAGGGCATGCCTC
CCTCACTCCTTTCTGATTCTGAGTAGCAAATCACTAATAGCACCTAAAAAATGGAATCAT
ATTTGGGTGCAAGTGCATAGTATAATCAAATTATTTCTTATATGTTACTTGTGTGTGTGT
GTGAGTGAGTGTGTGTGCATAAGGATAATTTTATTAACTACTTTTCCCTTGGTCAATAAG
AGCTTTCTGTAACATAGGAGTATTTAATGCTAAATTCTGAGAAACTCTTGTCAAATGCTT
AGTCTATGGAATCCTAGTAATGAAAAGGAACCTAACAGCATTGCCTATAGCCACTTTCAA
ATTGAATGATCACTTTAGACAGGCAATCGCCCTTTATGTCTTGAAAGATAGTTTCCATCT
ACCTCTTTCAGCTTGTTGAAATATCTGTCCTCCTTACTCTCAAAACTACTCCTTTTATAT
TCTTCCTGCAGTTTTAGCAACTACTCAAGCCTTTGATGTGTATGTGTGTGTGTGTGTGTG
TGTGTGTGTGTGTGTGTGTGTGTAACATGATTTTAACATCTTCTGTTACTCTTAAAATGG
GTGCATTTCAGTAATACATATACTGGTAAGAATTCTATAATTAGAATTTAAATATGCCAT
AAGACCTATAGTGTTAATTATGAAGCCATGTCACCAAATTCATTTGTAAAAACCAAACAG
ATATAATGTATGCTTGGATCAAGAAAGGCAAAGATCTATGGTTTCTGCAATTCTGGGCTC
TGATTCATTTTGAAACTTAGAACTCTGGGACCAATTCTGTTAAGTCACTTTCCAAGGATT
TGTCTTTTGCTGGTTTCAAGGTATACTCACCACTTCTTCCCGCTTGGTCTTGTCTGTCGC
TGGCCAAGCTTCCAGAGGCAGCTCAGGAAGAATGGGGGACAGGGGCTGCATGGAGAACAG
TGGAGGCAGAGGTGGCTGTGGTGCCAGGGGCTGCATGGGATGCAGAGGTGACTGGGGCTG
CATGGGCTGATGAGACTGGGGTGGAATGGCCTGGGGCTGGAAGGGCTGCTGGAAGGGCTG
CTGGGCGGATGGAGGGATGTTTGGCTGATGGTGTTGGGTTGGAGTCATGGAGTGGTGGCC
AGGAACTGGCATCATTGGTTGCTGGGGGGCCACGGGCTGTTGAGCTGGCACCACGGGAAG
GTGGTGATGAGGCTGAAGGGTGTGACTCGGGGGATGCTGTTGAGACAGCACAGGGATGAT
TTGGTGGTGCAGCCATCCACCCATGGGTTCGTAACCATAGGAAGGATACTGGTGAGAAAC
AAAGAGTCAGGTTTACCATTGGCTCAGTTGATTTCTAACTGGAGAAGTAGCAGTAGTGTT
TGTTATTATGACCTTATGAATATTTTAGTCATTTTTCCTTTGCAAGAATTTCTAACAGTG
TACACAGATATGACCTTTTACAAACTAAAATTCCCATTAGTGTTTGAAGGTGGAGTAGAT
ATGATATTTCAGTTAAAAAGGGACTTAAAACACATGTCTATTCTCTTAAGGGAAGGAAGA
ACAAAATGTTTACATACCGGCTGCCTTATCATGCTCTGGTACCACTTCAAAGGGGTAAGC
ACCTTAAGAGAAAGAGACATTAGGATGCATTTGATTCAGTTTAATTACATATGAACTAAA
TACTAAACTCACTAATGCAGTATGAAATATAGACTCACTAATGCAGTCCTGTCAGTATTG
ATAGCCTGAGAATGTGACTTCTACAAAAGAAAGAAGAAGGTAAACATTTGTAGGATGCTT
TCTATGTGTCAGGCACTGTGTTACATCCATCCCATACACTTTTCATGCTTTCTCGCATGA
TTGGAGGCAGGTATCACCTTCACTTTTATAGGTAATGTTATGAAAACTCAGGTAGGTTCA
TTCACTCAGCTGAAGTAACAAAAGCAGTAATTCAGTTTAATCCTCTTCATTGCAAGAGCA
AAACAATTGAAAACTTATAATAATGTGATATAATAATCATTACTTGTTTATTTGATATCA
CAATGGAAAAATGAGGTAGAAACATTGTCTTTCATCCTTCAGACATAGATTTCTTGGTCA
AATATATCAATCAACCTTTTCAGATAAAACACTAAAATTTGCCTCAGATCCTTATGATAT
TTTTCTTCTGTATATACTAGTTAACAGACTTAGGTTTTTTTTTATCTGATCCCATGAGAT
AAGTAAAGAGTTTCATTCCTTCTGGACAGTATAGTCTTATTAATTAGTCTCAGATAGCTC
TTTCATATAGTACATCATTTAACCAATACATCAAAAAGCTATTAACCTTACTCAAGTTAT
GAGTGACACAGCACTTTTCAAGCTCTTCTGGATGATATTTTTTTGGAAATGTTCTTATCA
TTGTTTCCCTTGTTTAGGTGAGGAAGCCAAAGTTACAGAGTGGGGAACTGATTTTCCCAG
GGTCATATAGTTTGCAGGCAGTTTAGGAGAACCCCAAATCCTGTTTCTAGAACATTTCTG
TGTTCCATCACTCTGTAGAGACTTTGGTAAACCACTTTTCATTGATCACTTAGCATAGCC
AATGAGAAACACACACAGTGTGTGGATGTCTTTGAATCAGCAATTTTTTTCCAGCTAGAA
AAGATTTTAACATCTTACTTACTCCTTGAATTTTATAGGCTGGCATAGAACAAGACTGTT
GAAGCTGAGTTCACTCTCTGCCCTATCATGGAGCCTTCCTGAGTGGTAGTTCATGGTGGA
TACATTTATAACTAGGTTTAAAAGTAGAGGTTGAGTCAGAGTGACCAGGTAGGAAGGTGC
GGAGAAATGCAGGTTTGAGGACATCAGTCAGGGCTTAAAGCTGCTGGTGGGAACTGAAAA
AGTGTGACCATTTGAGAAACATCTTGAATGAAGAATCAATACACCACTCTTTACAGAACT
CAGGGAATCTTTAACTCAAATAATAGTCAAAATTAGTAAATAGAAAAATTACCTCATAGC
TTAAGTTGATATAACCAGGGCTTCCAGGATGAGGTGGTAGCTTTTTTAGAAAGGAGAGAA
GGCAAAGTGAAAGAGAAAGGGAGGGAGAAGGAGAAAGAGGAAGAGAAAGAAAGAGGTTAA
GTCAATATGCAATTGTTCATATTAAGGTGTCTAAAGCTATTTTAATATATATATATATAT
ATACACAAAGTTTGCAGAGTGTATTTGAGGTCTACTTTTAGTCTGAGCATGTATGCTGTG

AAGGAGGAACCCCATGGTCCAGAGTCTTCTAGGGAGATCTAGATGCAGAAAGCATCATAC
ATTTCACCTCCAGAGATCACTGGGTTAAACCCATCAACTATCAAAGGACCCCTCCCTGTT
GTGCCCACCCATTTACTTGTTGATCAAACTCCGTGTTAAAAAAAAAGAGGTCTGTTTCTG
TGGCACAATTTAAAATGAACAATAAGCCAGGAATTGTTTCACAGACGGTTGTTTTATAGA
TCAGCTGGTTAAATGTTCAAGAATCAAAGGGATTCCCAGTAATTAACATGCAAAGTAAGT
AAATAATACACTAACTCCATTGTTCAATGTGAGAACAAGACGAGACTAATAAAATATAAA
TATTCTAAATCCATCCCCTGGACAATAAAATAATTGATTTATTGAAGTTCAAGGCCAACC
CTTTTTATTATTGTCAATTATTTATGGCAGAAATTTAGAAGTTCCAAATAAAAAGCAACC
TCAACAATTTTCTAAACCAGGACTCTCTGTGCTTCCCAAAGTAGGGGCCTGACTAGTGCC
TGTTTTATGGAGTTTCTTATGCCCTCCCCTGGTGATGGGGGCAGGAGACAGAAAGCTGTG
AGCATAAAGCTGAAAACCTCCCTCAGTCACAAAGCTGCTTAAGGGTCACTTCAACTTTAC
TGTTTCAAAATGCTCATGTTTTGCAAACGAATTTATTCACTCTCATCTGAATGTTAATTG
CATCAGTTGGGTAACTGTTTTCCCTAAACTGTATTGATATAAGTTCTCTGAGAGTTTTGT
ATTGAGAGGGTTGAGTTTGTAAATGCAGGAGCACCTTGGAGGACTAGGATTTCAGGAATG
CAGAGCACACAATCTTGGTTTTATGTGTGAGAGAAGTGTGGCTGATATACAAGCTTACAT
GTATACAGGAGTAGAATCAACTGCTCTTTATACCATTGAAGAGTATTGCTCGCATGTGAT
CTAAGCCTTCTGCCATTTTGTACTTTTGTCTAGAGAATGGACCAAACTTCCTGGTCTTTT
GTGCAAATCTGGTACTGTATTTGCATGCTGTCAATGTGTATTTGGCACAGGCAGGCAGGA
TTACAGTATCTTGCTACTGCCAAATCCTATATAAGGAGTAACCAGATCCACCAAAGGTAG
AGGGCTTCATTGAAGTGGCACAAGAAGAGAATTTTTAATGTATAAATATTACCCCCTTAA
GACAACAGCAGTGGCATCTGGAGGGCAAGAAGAAATTTTGGAACTTCTGAGTAATTAAGC
CTTAGAGATTTTTTTTGGGGGGGGGCTGTTATATTTGCCTAAAACAAGTAAACACTCTCT
GGAAGCCTTTCAAGGGTCCCTTCAAATAGATAACTACTTGTAGAGATGGGCAGTGTTGTT
TACAAGCTAATCGTTACAGGAAGATCTCCTAGGGGTCAAATGCTTGTTAAAATCTTGAGT
GCTTGGTCCCACTTCAGCTGAACTGAATCACAGTCTAAGGTGGAGACTTTCCTGATTTTC
TTTTTAACAAACTCAAAAATGATTTCTTCAACACCTATTAGATACTTGGGGATATAAGAT
CTTGCACTGTGTTACACAGTGAAAATTACCAACTATGGGAGCAGATTTCTAGTTCCAAGA
TTATTCAATTGGATGCAATTAGTAAATGGATTGTTTTACTCACGGGCATAGCAAAAGCTG
CTCCCAGGAGGCAGGCAAACAAAATCCAGGTCCCCATTTCTTGATGGTTCTGAAATGTAA
ATCAATACCAACCATTTAAAAACTTTTAAAAGCATGAATATAATTATGCTTCATACATGT
ATATAATCCACATTAATGTCTTGCTAATCAAAAAGAAGGTGCGGCCCTTATTGTAAAGTG
ACATTCATATGGAATTATCATTGCTTCTGGAGTACTAATATTGCAATGTGTTTATTTGTT
TAAAAACTAAGCTGAAGCTGAAGGTTAACGAGTTAGAGGACCAAAAGTGATCTACAAGAA
TGATGCTGCAAACCATTTTCTATGCTATACAGCCTAAGGATGCCTAGAAGCTAGATGGAA
ACAATAAAACTGATTAAAAGAATCGGGTTGAGTATAACAGTTGGAGAGATAATCAAATGA
ACATGGTTTTATACTTTGGTAACAAGAAAAGATAGAAATGTTGTTTTTCTTTTGGGGGGC
ATAACCCACTTGAGCATTGCTACTGTTAAATACATAGAGTAATTATTTGAGAAAATATTG
AATAATTTTACAGAATGGATTAATGGCAGAGTGGGTTTAGGTCCACTTGTAGAATTTTAG
GCAAGAATCTCTCATAATGCCTTTAAACTCCAGGTTTGAAGCAGAAGGCATGCTTAATCC
AAAGAAAGAGAATAGTGCTACCATTCTAAGCTTCTTTAAGGGTTGTGGAATGGTTTCTAC
GTAAAGAGTTCTGTCTTAAGCCAGTAGCACCCTCTTCTTGCTTGTCAACTATACTGTATT
CCTTCTACATATTAAGGCCTTTGAAACATGAAGCAAGAACTACCTATCCTAAAATATGCC
ATTGGTTTTGGACAACACCTTGAAAGTAAATTTAGGACATGAATGTAAAATGCATTGGTG
TTACCTATGAAAAATACATTGAGGATTTCTAACATCTAAACCTTTTCCCCCCAAAGGTTG
AGGTAATAAGAAAAAGGATTCACAAGATTTAACAAGATTTTGTAAAGAAGTAGCCCTCAG
CCTGTTAAAACAGCATCTTAACTAAAAAGCAAATACACATTTTTGTATCAAATCTGGCTC
CCAAAAGGCTTTGGAATGCTTGACCTAAAATTTACAGAAGTACATACATTACTGTACTAT
AAAGTTAAAACTTCTTTTCAATGATTTAAGTCTATAATTTCATTATAGTAAAACTAAAGA
AGTACATATGGTGTAGATGGCACAAAGAATTAGAAATGTGCAATTCATTCACACATTTGA
AACATGAAATTCAGAGAAAGAAATGACCACAGTGGAGATGGTAAATCATGAATGAAATCC
ACATACCTTTGAGTGTATGCTCAGTGAGTTTCTGTCTGAAGCTCAGGGATGCTTGATCCG
ATGGTTTCTTCCAACTCTGTGCCCCAATTTATATCATGCAGGGCACTTTGAACAGCCAAT
CAGGTTTCTGAATGAAAAATGTGTTGAGTCTAAAGAAAAATCCCTGTATCATGATTTTTT
TAGATGCCGTGAGCACTGTCAAGACTGCATGTTGTGATTAGTGCATATAGTCGTTCATTA

CAGGCAATAATAGGTTTGGGAAGCTGTCTTCAAGTCAGCAAATTAGTTGACTGCATATGC
TGATATATTTGAATTCATTTTTAAGAAAGTGCTATGCCATTGTCGACGTCTCAGTTCTAG
CAATTAACGTTACTTTTTTTTTTTTTTAAGTGAATGGTCAAGTTTCTCCAGTGTACTCAA
AACAGTCACTTAAGGTTCTCCAGACTTTTCATATCAAGCTGCATGGATTGAATCCCCATT
CTTGTAAAAATCCAAAGTTCGCCCAAAATTCCACAATCGCTGGTGTTCTTTCTCTGCTTC
TGACACAGATATTTTCTCTGTCCCTTCTGTCCTCCATTACCACTCTGGTCTAGAAAACCA
CCTGTCATGCAAACAGCCACGACCTCAAGACTATTACAGTAGCAACACTTACTCTAATGC
CATAAAAGACAAAGTTAACAAATGTCATATACCATACAATGTGCTTGAAAATCTACTTGA
ATACTTACAAGCGTGCTACAGGAAATATCACCCTGATTTTACTACAGAGCTATTTGGCTT
CTCTCTAATCCAGTCGTTCCCAAACTGTTGGTCGGTCATGACCCGTTGGGGAGGTCAAAT
GACCCTTTGACACAGGTCACCTAAGACCATTGGAAAACACAGATATTTACATTACATTTC
ATATCAGCAGCAAAATTACAGTTATGACATAACAACAAAAATAATTTTATGGTTGGGGGT
TACCACAGCATGGGGAAACATATTAGAGGGTCTCAGCATTAGAAAGACGGAGAACCACTG
CTCAAGTCTTTTCTAGACCATACCAATGCCAGATTCTTATATTGCAAACTGAAATTCCCT
TCACATTTCAAAGAATTGCCCCATGTTTTCAGCAGGCTCTGCTTTGTTTAAGCAACATTG
ACCAAGTCAGTTCACCTGCAGGGACCTCTGATTTAGCTACTCAGTTTACATGTATTGTTA
CCTCTTTACTGTAATAAAGCATGGGTTGGTTTTACTTTATTCTGCAATTTCATCATTTAA
GAAATAAATTTATAATTATTGACAGGAACTACTAGGTTTTGCGTCAATACTGAATTACTG
CAGTAACTGGTGATAGAGTTGGATCTTTTGAAAAACTGAGTTTATACATTTATATAAATA
TGCACACTTTTCTTTAGCTACATATATACATACTTTCTACAGATTCTTCAAATTGATAAT
ACAGGAACATTATTAAATTGTGTAAT

Amelx_FIMO

motif_id	motif_alt_id	sequence_name	start	stop	strand	score	p-value	q-value	matched_sequence
1	HTTCTRGAAVNTTCKYM	X	7322	7338	-	13.2121	5.64E-06	0.149	CCTCTGGAAGCTTGGCC
1	HTTCTRGAAVNTTCKYM	X	6200	6216	+	12.1697	1.81E-05	0.16	ATTCTAGTCAATTCTCC
1	HTTCTRGAAVNTTCKYM	X	9313	9329	-	12.1697	1.81E-05	0.16	ATTCAAGATGTTTCTCA
1	HTTCTRGAAVNTTCKYM	X	11880	11896	-	11.6121	2.57E-05	0.17	ATTCTGTAAAATTATTC
2	DGAANDDDCN	X	9232	9241	+	9.69697	2.65E-05	0.674	GGAAGGTGCG
1	HTTCTRGAAVNTTCKYM	X	11377	11393	+	8.84848	3.66E-05	0.194	TTTCTTGATGGTTCTGA
1	HTTCTRGAAVNTTCKYM	X	8924	8940	+	8.31515	5.52E-05	0.243	TTTCTAGAACATTTCTG
2	DGAANDDDCN	X	7562	7571	+	9.44242	7.72E-05	0.982	GGAACTGGCA
**# FIMO (Find Individual Motif Occurrences): Version 5.5.6 compiled on Aug 4 2024 at 17:26:56**									
**# The format of this file is described at https://meme-suite.org/meme/doc/fimo-output-format.html#tsv_results.**									
**# fimo --oc . --verbosity 1 --bgfile --nrdb-- --thresh 1.0E-4 motifs.meme Amelx_X_dnachromosome_chromosomeGRCm39X16.txt**									

>5 dna:chromosome chromosome:GRCm39:5:88633380:88656492:1
GGGCTGGAGAGATGGCTCAGAGGATAGGAGCACTGGCTGTTCTTCCAGAGGTCCTGGGTT
CAGTTCCCAGCAACCACATGGTAGCTCACAACCATCTGTGGTGAGATCCAGTGCCCTCTT
CTGGCCTGCAGGCATACATACAAGCAGACTGTATAAATAATAAATAAATCTTTAAAAAAA
ATTTCAAGACTTCTCACCCTGGGTTCGAAATTCATTCATTTATGTGTGCATAGGCTTTTC
CCTCCTGAAATAAAACACATCTTCAAAGGCTGGGGGGAAGGAGGGTAAACTGAGAAAGCA
ATCAGGAATGGAGTTTGGTGGAAGCTCCCAGTAGCCTATCTACTGAGGCCAAAATAAAGT
GTCCAGATTCTGTGGGGGACTGCACGCATAGCTCAGGGCTGTCAACACCATACTCCATCC
CCATTCCCTCTAAGTGACCTACTTCTTGCAAATTTATGTCAATGTAAACAGTGTTATGCT
CACTACTCAATTACTATAGAATCAGACAAAGGCAGCCTGACACTGCTGGCTTCCTTTATC
AGATACACCTTTGCAAATCTTGCATACATATAAATTGAAAAATATTTCTAATTTACTCAA
AGACTGTCTTTAAGTTCTTTCTAACACTGTCTTATTCTTTGTATCACTAAACTTTGGAAC
TACGGTCCCTAAAAACAGCTTTGTTGTATGGGCCAAGATGAGACATTCCACTCCCCATGC
CTTGGAAGAATGGTAAATATATAACCACTCGGTAGACAGAGACATAAGAAGTAGGAGACA
GTGGCTGGAGGAGGACATCAGCCCACCCACAAAACCCAAAATTTGACCTGCCTGCAAATT
GCAGAGGGATAAAGATGGAGCAGAGATTGTGAGAACAATCAACCAATGACTGGCCCAACT
TGAGACCCATCCCATGGGAGAGAGCCAACACCTGACATTATTAGTGGTACTCTGCTATGC
TTGCAGACAGGAGTCAAGCATAACTGTCTTCTGACAAGCTTCATCCAGCAGGGGATAGAA
ACAGATGCAGAGATCCCTAATCAAAGATCAGGCCGAGCTCAGGGAGTCTTATGGAAAAGT
GGGGTATAGGACTGAGCAATCTGAAGGTGTCAAGGACACCACAAGAATACCTATAGAGTC
CACCAACCTAAGCCCATGAAGGCTCACAGAGACTGAACCACCAACCAAAGGGCATGAAGT
GGCTGGACCTAGGCCCCCTACATATTTGTAGTAGATGTGCAGCTTGGTCTTCATGAGGCT
TGCTCAACAATCAGAGCAGGGGCTGTCTCTGACTTGGTTACCTGCCATTTGATCCCTTTC
TCCTAGCTGGACTGCCTGGTTGGACCTCAGAGGGAGAGGATGTGCTTCAACTTATCCCAA
GGCAGGGTGTTACCCAAGGAAGGCTTCCCCTTCTTTGAGGAGAAGGGGGATAATGAGAGA
AGGGATTTGTGAGGGTGTGACTAGAGGAGAGAAAGGAGGGGGCTGCAATCAGGATGTAGA
TTGAATTAATTAAGTAATGAAAAAAAGAAGTAGGAGACAGCTTTTGGCACTAAACTTAGT
TCTCAGTTTTTAAATGACAGCATTACACTAAGCAAACACACTCGGACAAATATGATCTAT
TAGGAAGATATAGAAAGACCAGAGTAACTCAGGTCAACCAAAAATGCAAAGCTTGCTCTT
GTCAGTAAAAAAGAAAATTGAATTTATTTAGAAGTGAAGTCTTTGATCAGGATGTAAAGT
GAATAAATAAATAACGATAAATAACAAAGGAAACTAAAAACAAAAACAAAAAAGTGAAGT
CTTTTTTATAAAAAAAAATCATGTTGCAGAAAAAAATAATACTAATCACAACTCCAAGTG
AATTCCTCAACTACAGTTTTTCCAAAGTTGTATGATCATAAACATCTGAGAGTTACTTGT
CATTTTGGCCAGCTCCTCTAAAAGTCTAAATCCTAACAACGAAGCTACATCTGATTTTTT
AAAAAGGAACTATACCCTAGGTTTTTATATAATGCACAGCAAATAACTGAGTTGGTTATA
GATGTAACATTCCAGTTTAATTACGTATTAACTATATATCAACTAGATCCATATCATTCC
AAAACCTTTTGATGGTGGCCAGTGATTTGTGTCAGTAGCATATGGTTAATAGTCACTTTT
GTTCCCAAAATATATTTGTATAAATGAAGTTTCTATACTTCAAAGCCCAAATTCACAAGG
AAAGCACCTTCTAAATCTTACTGCTGTAAGCTTGTTTTTCTGAGCTGTTATTATAGATAA
TAAGAGACTCCATTCCACAGGTTCTATAATAACACATTATAAATTTTGCACTGGTTTTTT
TTCCACTAACTCTTGTGATGGCAAAACCATTTTTCTCAAAGCTGATTGGTTGTGCATGTC
TACAACAGTGCTTAAATGTGATCCTGCTATCAAGCCTCCTGACAAGGAGGGATCTTTTTA
TGTTAAGACTTTTGGGTACTGAACATGACACCTTCTTGAAGAATTTTTGTTTTATTATTA
AGAAATAAATGTGTCTTGCTCCTTGGCTCTCTCTGGCTTCAGCAGTTTGTAGTCATTTCT
ACCATAGACCATTAAGAACATATTTCAAAGACAGGTTAACAAGGTTCAAGTTTACTTTTT
ATCTAGTGCAGCTTCTAAGTGGCATTGGGTGAGTATCAAAGATTATCAACATATATTGTA
TAGTGTTAAGTAATGTCCTTGTTTATAGATGTTTGTTAACTTTCCTATTGTATTTGTGGT
GTTTCTAAATCACCTTTGTAGACTAACTTTTGATAAAAGTTTCAACTCTTAGTTTATTTG
GTATTTGTTTCTCTTGTAGGTTTTCTCCCAAACTACTCTGACCTCCTTAACACTGTACCT
TTGCACATTTCCATAAAGAAGTTTTGTCAACAATGCACTAGCAGTGTCTCAAAATACGAT
GCCTGTCTCATTTTCTCTCTGATTTAGCTCTCATCCTAACCTTAAAGCTCCGAAGATTAA
GCTCGGTTTTGAGGACCCTCTCATAAGGGTATGGTTGATGGTAATAAAATGATTACCATG
ATTTGGGCTAGCTACCCTTAGAGGTGAAGTTTTGGGATTTGGCTCACTTATTTGCTCTCT
TTCCAAATTGGAGTTTCTTCTCATACAAAAATCCTTATTTTTCCCAAAAAGGCTTATGAA

AGTTTTGGTGAAAACATGTTGCTGCTTCAGTGCAGAAATCCGACTTCTCCTCCAAAGCCA
TGTGGCCTGGTGAGTATGCTCCCCATTTGTCTATTATCTCCTCATTTGTCAGACTATGAA
GTTCACTAAGTGCACCTGAGAGGACAAACCTCACTAAAGTATGTAAGACAATGCGGAAGA
TGCCAAAGTTTACAGTCGTACTTCAGAGCAGCCTCTGAGTATTATTTTCAATCAAAAATA
TAATCAAAACTAATCAAAAAATCAGCTGTTACTCAGTTATATGTTCTTTTACGGGTAGAA
AGGACTCATCTGACATAATTTGATAGTTTAGCAAAAACAAAGACATTATATATACACAAA
ATAAGTGAAATGTCTCCTTTCATGGGTACAGAATCTAGAATTACTGTGACTCTACTGTAA
AGATGAGATGAGATGGGTATCTGAATGAGATGAACACCCTGAGATACCAAAAAGCCTTGA
TCCACGACCTGAGAGATGTTGAGAAAGGAGATCCTCTACCTACTCTCTTTTCACTTCCTG
TGGTTTTGTAACCTACACTAAATATATCAGCCTCAGCAGTTACTTTTCTAAGGAAGAAAA
AAAATATACATTTCTGTCTCTTCATTCTGACTTTGGCAAGTAATGTCTCGAAATTAACAT
AAAGTCACAGTAGAATCTCTTTAGAGTTCTGAACTCTGACAGAAAAGCAAAGACATTAGT
ATTAGTACCTAACTGCAGAGTGCCCAAAGCACAGTCATTTTCAATGTCCCAAGATAATAA
TCAGTTTTATATTCTAGGTACCAAATGTAAAGATGAGTCTCCTTGTTTTCCTGGGTCTGC
TTGGTGTCTCTGCTGCCATGCCAGTGAGTATTTACGCCTGTGTTGGGTCTTCCCTTTGCA
CTCTGTTGTTGCTTGACTCAGGGACAGCTGGCAGATTATTTCAGCTAAGGCTCTGCATTT
CTCCCTCAGTTCCAGATGCCAATGCCCCGAATGCCTGGATTTAGCAGTAAAAGTGAAGAG
GTATGTATTATATTCTGTACCGGAGGAGACACGTGCCAGGTCCTATCTAAAAGGTCAGGA
AAAGGAGAGTATTGGTACTTTTCTTAGAAAATAAAAATTCTTATGAATGGACTAAGGGAG
GTAAAATTCAAAGTCTTGCATTCAAATCAAAGGAAAAAAAAATCCCCCGCCCCCGAGCCT
GCTATGTGTTTCAGTGTTGATATATGTTAAAAATTAGTTGTTAGCTCTGGGGCCAGCTTT
ATTCACTAGTAAAGAGCCATTTGTACTTGGGAGTGTTATAAAGTACATACTTGATTATAT
CTTTAGCATAACTCAACCTAGTTAGCATTAATCAAATTTCATGCTCATTAGAAACAGGAA
TCTTTGTATGTTTGCTTGGGTGTTTTGGTTTGGTTTTGAGACAAGGTCTCTTTATTATGT
AGCCCTAGATATCCTGGAACTCTCATCCTTCCTTCTTAATTTGGTTTTGGTAATCAATAT
CTGCTAGTTAATACAAAATTACAGTAGAATCCCTTTATATCTGTGAACTCTGCCTCAAAG
AAACATTAAGGAGCTAGTGCCAAACCAACCGTAGAGTTATAGAGAGCAGGCTGGCCTTGA
ATTCACATAGACCCATCTTATGGGATTAGAGGTGTGCCAGAGAAGCAGTTATCTTTTTAC
CATGAAATTATCTTGTTCTAAAATCCATAATTCTAAATAAACAGAACATTCAAAAGCTCC
AAAGTAAATTATATTTATTTTCTATTATTCTATATTGATTCAATATGAAGTCTGACATCA
AATTCAGGAATCTGGGGGCTAGAAAATTGTCTCAGTTGGTAGAGTTTGTGGTACAAGGGT
GAGTCTGATCCCCAGAACCTCCAAGTTTGGTGACAGTCACTTGTAATCCCAGCACTGAGG
AGTGGGAACTAGAGGATTCATGGACCTCCCTGGCAAGCCAGGGAGGAAGGAAAACCCAAA
ATCAAGGAATCCCCAGATACTAATGAAACTTAGTCTCATAACAGAAGGTAGATACCTCCT
AAGGAACAATATTTAAGATGCCAGCTTCCATATGCACACATATGAACACATATACAATCC
TGTGCACATGGGGCTGAAGAAATGGTTCAGTGGTTATGAGCACTGGATATTCTTCCAGAA
AACAGGGAATTTGATTCCTAGCCCCTACATAATTATCTGTAACCCCAGTGCCAGGGAGTC
TGACATCCTCATATGTCTTCTTTGTACAGCACACACACACACTGTAAGGCGCAGACATAT
GTGCAGGCAAACACCCATACACATTAAATAAATGTTTAATATTTTTTAAAACTATACATG
TATAAAAAATTACATACTCCAGAATTTTTTAATGAATACAAATTTTAAAGTAATATATAAT
TAAGAAATTATGACTATTGTATCTTCAGACCATGAACTCGATTTAATTCTTCTGTTTCGT
GAGTTAATTTGAACAAAAGCTTTTCATTTTATGCCACATTAATGGATTCCTTTCATTTCA
GATGATGCGATATAATCAATTCAACTTCATGAATGCCCCACCAGTAAGTCCTTTATACTG
TTTCTGACACTTTCTCTTTTATATCCAGAAGCTGGGCTGTGTTTGTCTTAGCAGATCACA
GTGCTAATTACCATCCACAAGTCTTAGTTAAGATAACCATTAAAATAGTGAAGGAGAAAA
ACCGCTTCATTTGTTCCTAGTGACAAACATTCTTAGTCATTTCTGGGTTTTTTTCCTACA
AGTGAACATCATCTACTAATAACAAATATTCGTTGTGTCTCAGAAAACTGTTCTCACTAA
AGTTTCTAGGAGCTTTGCACACGACCAATGGCAACCAACACAGGATAGTGGATACTATTT
GAGGAGAGACCTCTGTGTTATCTTACACAATTCTAAAACTGTGAATCTTTTTGTATTTTG
CTGGAGTGATCATGGGATATACTTGTGGTTGTATGCTAATTCTAGAGCAAACTAAACCAT
GACCGTCAGCTCATCTTCTAATAAGATTGTGTGAGGGGGAGTGGGTGGGCAGGGGAGTGG
GGGTGGGTGGATATGGGGGACTTTTGGTATAGCATTGGAAATGTAAATGAGTTAAATACC
TAATAAAAATGGAAAAATAAATAAATAAATAAATAAATAAGGATATAAAGCTAAAAAAAA
AAAAAAAGATTGTGTGATAGCCCAAAAATGCATATGTCATACTACTGTCGTGATGTGGTT

ATAGGCTTCCTCGATTCTGTCCTAACCTTTCTGTTCAGTGACTGTCTTAAAGTTTTTACC
TAAAGTCATCATTTTCAATGCTCCCCTCCTAGATTCTGGTTGGCCCAACTTTCCTTGCTG
TTTTTGTGTCTGTGAACTGAAATTTCCACCAATGTAAAATCGTGCATCTGTTTCCGGGTT
TTGCTTGTTGTTTGGTTGGTTTTGGTTTAGGTTGTTTTGGTTTAGGTTATTTTCATTTTG
GTTGTTTTATTTCATTTTTCTCTCATTTTACCTGGCCTTAGATATAGCAAGTCACATGAG
TTTTGAATTTCCATAAAAATATATATACTCAGAAAAACATAAGATTCTTATGCAAGGTGT
GGTGGTACACCCCAAAAATCTCCACACTCAGGATTTCAGTGACATGAAGATTGCTGTGTG
TATGCATGTGTGCAGACAAAGTGTGTGTGTGTGTGTGTGTGTGTGTGTGTGTGGTTCAGA
AAACAACTTAGAGCATCCGTACTTGCCTTTCACCTTTCTTGAGACGGTGTCTCTTTGTTG
CTTACACTGCACATACCAGGCTACCTGGACCATGAGCCTCCAGGGGTTCTCTTGTCTTCA
TTTCTTATCTCACCATAGGAGCACTGGGATTATCACACTATTGTGCCCAACTTTACATGC
ATGGTTCTGGCTAGACAGAATCAGGGTCTCACATTTGTGCACCAAGCACTTTGCCCACTA
AGCCATTCTTCAGCCACCAAGTTCTTGAACTGACATCTCTTATTGAAGTCTGCCTTTGAT
TCGTTTGACAGGCTTTCTAGAAAACTGAGGTCTCATTCAGTAAGACAAAATGAAATGAAG
TCTTCGTTAAAGATCTTGGAATCAAATTCCACTGGAGTTTTAAGCTTCTTGACTTGGTTT
TGGAAGCTTGACTTTAGAAGGTGGGACGAAACTGGACTTCAACCTCATTCGCTGTTACTG
CTTTTGCTGCTCTTTCAGATGATGCCTATGGGCCCATATGGAAATGGTATGCCAATGCCG
CCACACATGCCTCCACAGTACCCTCCATACCAGATGCCCATGTGGCCTCCACCAGTACCC
AATGGATGGCAGCAACCCCCAATGCCCAATTTCCCAAGCAAGACTGATCAAACCCAGGAG
ACCGCCAAACCCAACCAGACCAATCCACAAGAGCCACAGCCACAAAAGCAGCCTTTAAAG
GAACCACCAAATGAAGCAGCACGGGCCAAAGATGACGCCCAGCCACCTCAGGTGAGGCTA
GAGCAGAAAACTTCCATATCACATGCATAAGCATACCTTAAAAGAGAGGTCCCCACCTGT
CCAACATACATACTTCCTCCTAAATTTAGTACAGGTATTGCTATGTAAGAATATGATATT
TAATTTTGACATTCATCAAAAGTTGCCTGGCAGGTCAACTCCAACCTTATTTAGTTCAAT
ATTACACAAATATTATTGCCTTAAAGATCCTTTTAAAACACAGAATCAAGCTGTGGTTGT
AGGATATGCCGATAACACCAGCAATAGGAAGGCTGAGGTAGGATCATAAGTAGAAGGCCA
CCATGGCTATGTGGTAAGATACTCCTTGAAAATAAATAAGAATAAAATATAATACAGTCT
TGCAATATAGAGTCTCATGACTACTAGTAGAAGAGTCAGGAAAGAAAGATAAAGTTATAC
CCCAGCCTTCATTGGTCAATTATACATATAACAAATACAAACTTAATAAAAAAGGCACTC
CTAGATTTGAATCTCGTTTTCTGTCATTTCTCTGCCATTTGGGGCAACTAATTTCTCTGA
CCTTCAGCTTCTCAAGTGAACTGAGAATAACAATGATGCAAGGTTGAAATCTGAAAGAGT
CCCATAACCAACTACCATTTGAATACAAGTGCTTAATAATATGACTATTACCATCATCTC
TACTCAAAATATGCAGAGAAGGAGGGAGGGGAGAAGGGGTAGAAGAAGAGAAAGAAAAAG
AAATTTGATGCAAACAAAAATACAAGCTGTCTCTAGTTTTCAAATAATTATTATCAATTT
CTGTCATTTCTCATTTTCAATGTCACTGCCAAATAACTTTAATTTGTCTTTCTTTCTAGC
CATTCCCACCATTTGGCAATGGACTTTACCCCTATCCACAACCACCATGGCCAATTCCAC
AGGTAAGAACTTTCTATATGCTATAAATTAAAAATAATATCAGTATGTAAAAATTAACAT
AAGTTTACATACTGACAGCAGAGAATATGAAACAAAATCAAGTTAGTAATTATTCAAAAA
ACTGATTAAAAATTGAATATATGTTTTACTCAGACCAGTTTGCAACATGAACAGAGTCAC
TAGTAACATAATTTAACTGTTTAATTTTAAATGGTTGTACTATAATGCCTCACAAAAGAA
ATTAAAATATCTTCATTGAGTATCATAATTTATCATAATGCAAAATAGCACTAGACAAGA
AGAGAGTTTAAAACTAAGAAGCAAGCCATGATTACATTGTAATCTCCCCATCATATTTTT
TTCTATCAACACAAACGTCATTATAAAGAAATCTGCAAAGTAATACATGAATTCTCAAAG
AGTTCTTAAAGTTTAAAATTAACAAAATAAAGTATAATATTTAACTTATTTGCACAAAAA
GAATAGTAAAGGCAGTGGTTTTCAACCTTCCCAATGCCAAACCTTTTAATGCAGTTCCTC
ATATTGTGTGACCCCCAAACTTATTTTATTTTCATTGCTACTTCATAACCATGATTTTAC
TATTGTTTTTCATTATAATGTAAATGGCTGTGTTTTCTGTGCTCTTAGGTTACCTCTGTG
AATGGGTCATCCAACCCCCAGAGGGGTTGCAACCCACAGGTTGATAACCACTGAGTAACA
CAAGCTGTCGAAAACAGCTACACACCAAACAGTACTCAGTGACTGTTTACTTGGCTATGG
AACAGAGTGTTTGTTTGTTTGTTTGTTTGTCTTGTAATTTACTGATCAAGAGGGTTTTAA
TGAGAAAAAATTCACTCCTTACTCCTTTTCCTTAAAAGGGTGGCTTTGCCACATTAAAGG
AAGGAAACATTCCAGAGAGTTTAAAAGCAACATGATTTCCATGTTGAAATGACTATTAAT
CCCATTACATCAATCTCTTGATGAGAAGCAAAAACTGCTTCTCAACCCTTGCTTCTATGT
AACTATCCCTAAGCTTTCATTTCTTTAAATTTGATGTACTCTTATTTTTTTTTAACTTTT

GACATTAGTATACAAAATCCCAGTTTTGTTATAGCACCTTCTGTGTTTGTGTGTTGGTAT
TGTGTGTCTGTGTAGGTGTGTGCCCCTGTACACATGTGGAGACCAGAGGATGGTGCTCAC
TGTCTTCCTCAGTCACTCTCCACCTTAGTTTTGAGGCAGGGTCTCCTACTGAATCTGGAG
TTCACATCTTTGGTTAGGCTGCCTGGCAACCAGCTCTAGGGATCTGCCCATCTCTGCTCC
CAACACTAGAACTGCAGGTGTCACGCCCAGCCTTTTTATAGGGAAGAGTGCTGGGAATCT
GAACTCTCGGGTGCTCAGCTTTCATGGAAAGCAATATTCCAACTGAGCTGTCTCTTCAGC
TCCTCAGCACAGCACTTCCACATACATTTGCCTTCTTACTTTTTTCTTTTTTTTTTTTTT
TTTTTTTTTGTTTGTTTGTTTTTTGCTTTTTGAGACAGGGTTTCTCTGTATAGCCCTGGC
TGTCCTGGAACTCACTCTGTAGACCAAGCTGGCCTCGAACTCAGAAATCCGCCTGCCTCT
GCCTCCCGAGTGCTGGGATTAAAGGCGTGCGCCACTATGCCCAGTTTTTTTTTCTTATTC
ATCCTCCTCACCAACTACGTTGTCTCCTCCTTGGCTCCTCTTTTGCTGGTTGCTTACCTT
GCTTCTAAAAGTTCCTACTTTCACTTTCATGACACATATATTTTTTATTAGATATTTTCT
TCATTTACATTTCAAATGCTATCTCGAAAGTCCCCTATACCCTCACCCTGCCCTGCTCCC
CAACCCACCCACTCCTGCTTCCTGGCACTGGGGCATATATATTTCATTACTCTTTTCTGC
CTCTCTCTTACTTCCTTTAAGATCTCTTCATCCCCTCTTATGGGCCCCCTTCTACTTTAC
TGAGTTGAGCACATGTGTACACACATACATGTGCGCGCACACACACAATCTAGAATCCAC
ATGTAAGCAAAAATGTGGCATTTTTCTTTTAAACTCAAACATATTATGCTTAACGTGATG
ATCTCTTAGACCCATTTTCCATAAGATGTCATTATTTCATGGCTAATTAAAATTCCTTGT
GTAGACTGGTAGTGGTGAAACATGCCTTTAATCCCAGCACTTAGAAGGCAGAGGCAAGCA
AATCTATGTGAGCTCAAGGGCAGCCTGGTCTACAGACCAAAGGCTATGCAGAGAAATCCT
GTCTAAATAAATAAATAAATAAATAAATAAATAAATAAATAAATAAATAAATAAAATTCC
TTATGTAAATGCACATATCTCTACCCAGTTTAAGAATCTTTGTGATATGTCTAGTGCTGC
AGATATAACCTCAGAAATGGTCCTGCGAGGTTCAATAGACAGCTCTATTTTTAGATTTGT
GAGTAACTTCCACTCTAGCAGCCGTAGTGGCTGTGTCACTCCATATTACCACTTGCAGTT
TCTAACAGTCCTCTTCCCCACATCCTTGAGAGTGTTCCTTAACATTTGTGTGCTGGAATG
AAAACTTTTCTATGTTGAGATGCTATCTTAAAACAATTTAAATTAGTTCTGATAGTAAGG
AATGTTCAATATTATCATAATTATTGGCCATTTGTATTTCTCCTTTTAGTAAGTGTCTCT
CCACTCCATTAGACCATTTACTATTACTTTTAATATTCAATGTTTGCAGTCCTTTCTACA
TTCTTCACATTTATCTCTTAAGTATAGTTGGCAATGATTCCTTCTCACTCTGTAGCCAGT
ATCTTCACTCTGGTGACTGTATACTTTGATATGCAAAAAGCTCTTTAATCTCATGTAATC
CTCCCATTTGTCAATTCTTGGAAGTCTACTGTGCTACTGGAGTCTTTGTCAGAAAATTCT
TAACATGTCTGAATCTTAAAATACTTTATATGTGTTTTCTTTTATTAGCTTCAAGTCTTA
CATATAAGCTTTCCACACATTTAGAGTTGGTTTTGGTGTAAGGTGAGAGATATGGACTTA
GTTTAATTCTTCAACAGATGGATAAAGTTTTCCCAGCACCATTTGTTAAAGAAGTTGTCT
TTTTTTTGCAATGTATGTTTTTGACACCTTTATTGAAAATTAGGTGTTTGTAGATATGTG
GGTTTAATTCTGCATTTGATTTTCTATTTTTTCTGTTTTCTTCTATTTTCTGTTTGTACC
AATGTCATACTAAATTTGTTACTATAGCTCTGTACTGTATAGAGCTATATCAGGTATAGT
GAGATTACCAGAATCTCTTTCCATTTAGAATTTTTCTACTTATTTTAATCCTTTGTATTT
TCACATAAAAAATTGTATAATTATTCTTATTTCTGCAAAGGATATAACTGAAATTTTGAT
GGATATTGAATTAAGCATATGGATCACTTTTATTAATATGATCATTTTATAATCTTGAGT
TGATAAATCCATGTACATTGACTTTTCTTATATGAGAATTCATATTTTGTTAAGAAAATT
AACTTCAAGTGTACACAAAGAATGTTAAATATAACATTACAATTTAATCTCATATTGTTG
TGTGCTCTAATATAGATACTTGGAGCCCACTATTACTCAAATATTAATATCTGTCTCTAT
CTTTCTCTCTCCTTTCTCCTCCCTCTCTTCTTCTTCCCTCCCCAGAGATAGTGAGGTAAG
GTGATTGATAAGACAGGATATTTTTAATAAGATAGCCTAGGGTCATCTTACAGCTTTATA
AAAATAGTACTCACAAATGGATCATAAGTATCAATAGGTAGTTAGTGAGTTCACAAAACG
AAGCATCATGACTAATGAGCCAGTTAAACATACCTCCTGACTTACAAACAAATGGCAACA
TAGAAAGTGCTTTCCAGAGCTGAGGAGTGAGCTTAGCGGGTCAAGTGCTTGCTGTACAAG
CATGAAGACTTGAGTTCAGATCCCCAGCACTCCCTCTCAACCCTAGTGCTGAGGAGAGGA
GACAGGAGAATCCCAGCTCTTGCTGGCTCAGCTACTCTAGCCAACCAGTGAGCTCCATGT
TCAATGAGAGATCCTACCTCAAAAGGTAAAGTGAAGGAGGAAAGAAGACAGCCAACACCA
ACCTCTGGCCTCCACACACGTGCGCACATGCACATGTACACAAACACACACGCACAAGCA
CACATAACACACACAATCATACACACATATGCACGCACATACAAACACACACATACACAG
ACAATCACACACACAAACACGCACACATAGACACAAACACACATATACATACAATCACAT

ACACATTTCTAGTCCAGTCTAATGGTGCACAACTGTAATCCCAGCACTTAGAAGGCTGAG
GCAGGAAGATTGCCAAGAACTCAAGGACAGCCCAGATCCATAATGGTCCTGATATAAAAC
AAGATCCTGCCTTTAAAACAAAGAGAAGAAAGCAAAGTGGTTGGTTCCTTGCTCACTGTG
CTCTTCATCTTTCAGAGGGGACCACCAACAGCGTTTGGACGGCCAAAGTTCAGCAATGAA
GAAGGAGTAAGTACGAGTGGCTCTGTCACCCTGGTTAAGAACTATAGTCTATCAGCTGCT
ATCATTTAACCCAATGCCACAAGAACTTAAAACCATCGCTAAAAACGTCTTTAACTCGAG
CTCTCAAGTTTTTTTTTTTTTAAGTGAAATAAAATCAAGCCAGTAGTGTGACTAAGTGTGA
TGCAGTGCTGGTTGTGGCAGCACCGGCCTAGTCTCAGCACAGCAGGCAGCTGCGTGGCTT
TGTCCCTAAACCCAGCACCACTTCCATGGTGCATAATCCCTCTACATGGATTGAAAGACT
GTACACAGATTTGCTGTGGAAACTATTAGCTTCTTTGCAAACCTGATAGTGAACCCTAGA
AAGTGAAACACACAGAAAGAAATTAAGATCATCTTAGAATATTCTCTTCACAGGAAACTA
CATTATTGTCTAAATATCTCATTGAATAAATACTGAAATCCAGAGAACATAAATTAATAT
CCAATTTTTCCTCTTGGGTTACAGGATAAACTGGACCAAACCCAAGTCTTATGATACGAT
ACCATTTTTACTATATTCCAGGAAAGTAATCAAGTATGCCCAGTATGCCAATTCTTAGTA
TGTCAGCAAATAAACTGTTCTCGTGGTCCACCCTGGACTTGGCTTACTCTTTACCTCTAG
CTATGTGGCATGCACTCCGTGAAGGCAGAAATGTACACCTTTGCCGGAGGAGGAAGGAGC
AAGGTATCACTAAAGTAATGGTGGTGAAATATTATTTCAATAATATCACGGGACGTTTAA
AATACTATATCACTATAAAATACCCACGTAAGAGTGTCTCAGGCCATTGTATACAATACC
CTTACTTAGAAACTAGAACTTCAGATTTTCATAAAATTTAGCAAGTAAAACAGAGTAATT
CAGACATAAACATAGGCTTAAATCTGTGTCCTATCTCTTGGGAAAACTAAGGCTGGGGAT
ATAGCTCAGTAGTAGAAGACTTAACTACCATGCCTATGTTTAATCACCAGCACAGGGGAA
AAATTAAATAACCAAATGATACACACGTCTAAAAAAATCACATAGGTTGTTAATTTAATT
CAAAATTATTAAGTATTTCCCACTGGTCTAGAAAACTAGAATAGCAAGACAATATTTTTA
CTTGAAAGCTTCCCATGGAGATGTCAAGTGGGAGGAGCCCAGTGAAATAAGCAAGTCAAA
CCTGCTGCGCTGGTAACCACAGCCGAAGGCTGCAGACATGAGCAGCAGAGCCTGAGTCCA
GCACTGTTCCAGGAGCCAGGTGCACCTGGCTGTGGCACTCAAGCTGCATCTTGAAGTCAA
GTGTGCTGGGAGGGCTGAGGGTGTAGGTGGGAAACAGTTAGATAGGGAGAGGCCATGTGC
AAAGACTCAAGAGAATTAACAGCGGAGGCAATTTGCAACCACATCAAAGTTTAAGGAAGA
GGAACTAAGGTTAAAGGTCATGGCCATGACTCAGATGATTGTAAAAGTTTTAAAATGTTT
AGTTCAATATGTAGCATTTATTAGAAACAAAATTCCAGATATGAAGATCTAGAGAAAGAT
ATAGATATATTCTCATTTGACTCTCAGGACAAATTGTATGGTTTTTACTATCCTATTATT
ATTGTCTCCATATTACAATTGAATAATGTGAATTTAGCCAACAGTTATAGCCCTAAAAGT
TGTAGAAAGTCCTGCATTGCACATGTATATAGCACCAAGGCATTCTGCTTTCTGTTATGC
TAAAGAATTTATAGTTTATCCTTTAAGGGAAAGGCAATTCTGAACACATATTTAAAGAGA
AAAATGTTTCCATTCCCAGAAACAGACCCCTAGTGTCTCCTACTCTGATGGAAATCATAA
AGTAAAGTAAGATTACACATACAGTGGTCACTGAGAAAGCAATGTCAATAGTTTGGGAGT
GGTAGGCAAAAAAAAGACAGGGCAAGGCTGAAAGTTCAGATAGAACGTTTGGACTTGGTA
ATTAACTGTCTGGGGAAATAAGATAGAAAGCAGAAGCTGGGGCAACTGGGTTTGAGATTA
GAAAATGCTCCTTAGATGCAGGATAATGACGTTTATGGGGAGATGAACCCAGTTCTGGGT
GCATTCAGTTGGAAGAGTCTGGAGCCTTACTCATTGGACCTTCGGCAAGAACTGTAGGAC
AGGCTTTTGCCTTTGTGAAATAAAAGCATGTATCCATATTTGAAGTTCAGGCTCAGCAAC
ATAGGAGAGTAAAATGAGAGCACAGAAATGAGGAGAAAAGGGGATGGAATCAAAAACAAA
AACTATTAATCATGATGTTGCCCTGCCTGTAATCCTGGCACTCTGAAGGCAAAGACAGGA
GGATCCCTGCAAGTTTTGCACGACACTGATCCACATAGCAAGTTACAGGCCAGCCAGGGT
TATATGGCAAAGCTCTATTTCAAAAATAAATAACTAGATACAATATCAAGAATTATAACA
GTTAACTTGGACACTGTAGCTCACATGTGTAACCTCAGCACTCAGGGGCTCAAGCATGGG
GGTTTTCACACATTCAAAGCCTAAGCAATAGTCTCATACTGTGAGATCCTAGATCAAAAA
GCATTTTAACCCTAAGGTTCAGGATGTGGGCCTAAAGGCTAGAGAGACTACTCAGCACCA
AAGGGTAGAACAGAGGCCTCGAACATCTAGAGGAGAGTGTAGGCTGTCATGGTATGAGAA
GCCTGTGGAGGAACTTTCCCAGTTACACTAAGCAATTCCAAGCAACACCATGGAAGCAAG
CAAAGAATGATTCTCCTTTCCTGTCTTCTTTCCTATAGAATCCTTACTATGCATTTTTTG
GATATCACGGCTTTGGGGGTCGTCCTTATTACTCAGAAGAGATGTTTGAAGATTATGAAA
AACCCAAAGAAAAAGACCCTCCTAAACCAGAGGACCCACCTCCAGATGACCCACCCCCAG
AGGCCTCTACAAACTCAACTGTGCCTGATGCTAATGCCACTCAATCAATTCCTGAAGGCG

GAAATGACACTAGCCCAATAGGAAACACAGGCCCTGGGCCGAACGCTGGGAACAATCCTA
CAGTTCAAAACGGTGTCTTCCCTCCCCCTAAAGTTAATGTTTCAGGCCAGGGAGTACCAA
AAAGCCAAATTCCGTGGAGACCAAGTCAGCCAAATATTTATGAGAATTATCCTTACCCAA
ATTATCCTTCAGAAAGACAATGGCAAACCACTGGTACCCAGGGGCCTAGACAGAATGGAC
CTGGCTACCGAAATCCACAAGTTGAAAGGGGTCCTCAGTGGAATTCCTTTGCTTGGGAAG
GCAAACAAGCTACTCGTCCAGGAAACCCAACTTACGGTAAACCTCCCTCTCCTACCTCCG
GGGTTAATTATGCAGGAAATCCAGTCCATTTCGGAAGAAACCTGCCAGGGCCAAATAAAC
CCTTTGTGGGAGCCAATCCGGCCTCAAATAAACCCTTTGTGGGAGCCAATCCGGCCTCAA
ACAAACCCTTTGTGGGAGCTAATCCGGCCTCAAACAAACCCTTTGTGGGAGCCAATCCGG
CCTCAAATAAACCCTTTGTGGGAGCCAATCCGGCCTCAAATAAACCCTATGTGGGAGCCA
ATCCGGCCTCAAACAAACCCTTTATAGGAGCCAATCCGGCTGCAAACAAACCATCTATAG
GAACCAATCCAGCCGCAAACAAACCATCTATAGGAACCAATCCAGCTGCAAATAAACCCT
TTGTGAGAAACAATGTAGGTGCAAATAAACCCTTTGTGGGAACCAATCCCTCCTCAAACC
AACCATTTCTGAGAAGCAATCAGGCCTCAAATAAACCATTTATGAGAAGCAATCAGGCCT
CAAATAAACCATTTGTGGGCACCAATGTGGCCTCAGTGGGTCCTAAACAGGTCACTGTTA
GCCACAATATGAAAACTCAAAATCCAAAAGAAAAGTCACTAGGTCAAAAAGAAAGAACAG
TCACTCCTACCAAAGATGCAAGCAACCCCTGGAGAAGTGCTAAACAATATGGAATTAACA
ATCCAAACTATAATTTGCCTCGCTCTGAGGGCAGCATGGTAGGCCCAAATTTTAATTCCT
TTGATCAACAAGAAAACTCCTACTTCTCAAAAGGAGCTTCCAAAAGAGTACCAAGTCCTA
ATATACAAATCCAAAGCCAGAATTTGCCCAAAGGAATTGCTTTAGAGCCAAGAAGAACCC
CATTTCAATCAGAAACTAAAAAACCTGAGTTAAAACATGGTACACACCAGCCTGCATACC
CTAAGAAAATCCCTTCTCCTACAAGAAAACATTTCCCTGCTGAAAGAAATACCTGGAATC
GTCAAAAAATCCTTCCACCCTTAAAGGAAGACTATGGGAGGCAAGACGAAAATTTACGTC
ATCCGTCCTATGGCTCTAGAGGAAATATTTTTTACCATGAATATACCAATCCTTATCATA
ATGAAAAATCACAGTACATTAAAAGCAATCCATGGGATAAGAGCTCTCCCAGTACTATGA
TGCGGCCAGAAAACCCACAGTACACCATGACTTCTCTAGACCAGAAGGAGACAGAGCAGT
ACAATGAAGAGGATCCAATTGATCCAAATGAAGATGAATCTTTTCCAGGACAAAGTAGAT
GGGGGGATGAAGAGATGAACTTCAAAGGAAACCCAACAGTTAGGCAGTATGAAGGTGAGC
ACTACGCCTCAACCCTAGCGAAGGAATACCTTCCTTATTCCTTAAGTAATCCACCAAAAC
CCAGTGAAGATTTTCCTTACAGTGAATTCTATCCCTGGAACCCACAGGAAACGTTTCCAA
TATATAACCCAGGTCCTACTATAGCACCACCCGTGGACCCCAGAAGTTATTATGTTAATA
ATGCCATAGGACAAGAAGAAAGCACTCTCTTTCCTTCATGGACCTCCTGGGACCACAGGA
ATCAAGCTGAGAGGCAGAAAGAGAGTGAGCCATATTTTAACAGAAATGTCTGGGATCAGT
CAATAAATTTACACAAATCTAATATACCAAACCATCCTTATTCCACTACATCCCCTGCTA
GATTTCCAAAAGATCCAACATGGTTTGAAGGTGAGAATTTGAACTATGATTTGCAAATTA
CTAGTTTAAGTCCACCAGAAAGAGAACAGTTGGCTTTCCCAGACTTCCTGCCTCAAAGTT
ACCCAACAGGTCAAAATGAAGCACACTTATTTCACCAAAGTCAAAGAGGGTCTTGCTGTA
TTGGTGGCTCCACAGGACATAAAGACAATGTGCTGGCTCTACAAGACTACACTTCATCCT
ATGGTCTTCCACCAAGGAAGAACCAAGAAACCAGTCCAGTGCATACAGAAAGCAGTTATA
TCAAGTATGCAAGACCTAACGTTTCCCCAGCAAGCATCCTACCTAGTCAAAGAAATATCT
CAGAGAACAAACTAACTGCAGAAAGCCCAAACCCAAGTCCATTTGGAGATGGTGTGCCTA
CTGTGAGGAAAAATACTCCATATTCTGGAAAGAATCAACTAGAGACAGGAATTGTGGCCT
TTTCTGAAGCCAGCTCTTCTCAGCCAAAAAACACGCCCTGTCTTAAAAGTGACCTTGGAG
GAGATCGGAGGGATGTTCTGAAACAATTTTTTGAAGGCAGCCAGCTGAGTGAAAGAACTG
CTGGCCTTACTCCTGAGCAGCTCGTCATTGGTATTCCTGATAAAGGCTCTGGCCCAGATA
GCATACAAAGTGAAGTCCAAGGAAAAGAGGGTGAGATGCAGCAACAAAGGCCACCTACCA
TCATGAAGTTGCCATGCTTTGGCTCCAATTCAAAATTTCACTCTTCTACCACTGGACCTC
CAATTAACAACAGAAGACCAACCCTACTTAATGGTGCTCTCTCCACACCCACTGAAAGTC
CTAACACATTGGTTGGGTTAGCTACTAGGGAACAACTTAAAAGTATAAACGTAGATAAAC
TTAATGCAGATGAACACACTACACTCGAATCTTTTCAAGGAACCAGTCCACAGGACCAAG
GCTGCTTACTGCTTCAGGCTTAGGGATCGCTTCAACTGAACATCCTTGGGGGATGAAAAA
AAGAAAAAAACTAGCAATCCTATGTCAATGATTCTGCAACTTTGTACCCTGAGACTTGAT
TACCTTCTTATGACCCCTAAGCCTTTTTCTCTCCCAACAGTAGAAATAGTGGCCCAGGTA
GAGTTAATGAGATTGGGCCTCTGGGGCTGGCTGGATCCAGCACTGTAACAAGTTAGTGCT

CACTTAAGAAAAAGCATGAAGGCCATGACCATTCCTGCCTTGAAACTTGGAAGCTATTTG
ATTGTCTGAGTTAGTTCCCTCTTTCTTGTTAACTTATCCTGTTTCTCTCAAATTACTTAC
AATGCTCTCTGGTTTTGCACGTCTAGCAGGCTGTTGAGTTTTCCATTTTCATACAGAGAT
CTGCACACCAGTATGGTCCTTATGCACTCTTTCCTGTATGTTGTTTCTGTGTGTTCTATT
TCTAGTTAAGAGTCTCTTTGGCATGTTTCCTCCCACTGTCTCCTTGCTCTCTTCTCTCTG
CTTCCTTCTCCATGCAGCTCGGTGTCCCTCACATTCTCTCTCTGTCCGTCGTTTCTCACA
TGCTGAGATACTGACTGGCACCACTGGCAGTCCCTTATGTCTGATGACCCCTTCATGATT
TCCTTTCCCTTCAATTTTCTGTTCTGTCTCCAAATGTGGAACTTTGATCTTTTACCAAAA
TTAACCTTTCCTCTCTATTTTTTGTTAAATTTCTTTTTCCCTCACTTTATATATACAGTT
TTCGTGCGTTAAGACTATACATCACCTTCACTCTCTGAATCCACTCTGCAAATTCTACTC
TCTTTTTCTCAGCCAAACCATCACTGTAGATAACACGTCTGAAATAGCTGCTTTTGTTGT
TAATTTTCTCATGACCTCTCCACATAGACACCCTGGTTGTAAAAGCACATTCTAGGAGAA
TGGAAGACAGCCTAGACACCAGATAGATCATAGAGCTTAGGAAAGTGGAAAATGAAGAAG
AGAAGAAATTGTCGCTCTGGGACTGAGCATCTAGGAAAAAAGACTCTCACTGTACTGTGT
CATCAATTTTCAATCTCCAAGATCTGCAGAGGACATTTCTTGAAATACTACCTTATGCAG
ATAAAGCACTCAATGTTTAGTTTTCTCTACTAGTTCAGTATAAATTATTGTACCATTATA
TATTATGACTATTATGTTTATGCTTCATATCCTTGTAGATTTTTCCCAGACACAACTTCC
AGGTATCATGATTAAATAGTGTAAATTATTTTGTAAAGTAGATATTTCTATATTGTCTTG
CTTAGAAAATATTTGAGTTTTATTGATATTATTTTTACTGTTATTAAAGGCATTTTGGCC
TGTTATCTGAATATTTCCTGTTTTGAGTCTTGGTGTACATTTTTTTTCTAAATTACCTAC
AATGTTACTTTCTATTCCTAAAGACTGTCTTGAGAAAATGTTCCTTTAATAGATTTTATA
TGACCCCAGAAATTTGGTGAAACTTTCCTGAGAAAATTAACTTTGCCCTTGACTCCAGTA
TAATGTCTTACCAATAGGAGATTCCTAGTAAACGTTTACTGGTTGGCTGTAGTGTCACTG
GCTCTTTAATGCATGTAAGCTCACACCGCCACTTACTCTTCCTGTGAGAGGTGTGCAGGG
ATGCAGAGGAAAGCACCATGTTTCCTAGCCTTGTGTTCAGAAGCACCTGCCCTGGCCCAC
TGCAGACTGTGCTGCCCGCTGATGGCCTAAGTTCCCTCTCACCCTACAGACTGTGCTGCC
GGCTGATGGCCTAAGTTCCCATTCACCCTACAGTTTGATACTTCAGTCATATTTAAAACA
ACAATAACACTAGCTTTACCCAAAAGTTTCACAGTGAATACTTGTACTATCAAATGTTCA
CTTTGTGTTTCACCATAATGAATGGGTAGCACAGTCATTTTGGCAAATAGTATCATGCCA
AGAGTGATGAGCCCAAGGCACAGAGATAGAAGTATTTATCAAACCAGAACTGATATAAAA
GTTTTAGTGAAGAACAGACACTGTGGACAAAAACATAGTGGGCAAGGTCTCATGCTCAGA
GTCCCAGAGATGAAAAGGAATAATGTGGTTCTTTCATAACAGTTAAGAGAGCTTGCCCTG
TGAGCCATTCACATTGAGATGCATCAGAACAAAGTTTGAGATGGCAGCTCCCTATCTTGA
GGTAGTCAGTTTGATATAACATCATTTGCCTCGTTGGGGACCATTAGGAATGAGACCCTG
ATTAAAAAGGTGTTCTAAGTTACTGGGTCTGGCGCCAAGCAGTAATGATCCAACAACTGC
CAACTGAGTAAGTTTCCTCTGTAGGGGACTTCGGTTTTTTGTTTTCTTTTGTTTTGTTTT
TTTTGCTGTCTTGTAGGTTTTTTTTTAAATTAATGTAAATTCTTATTGTTTTGTGGTGGA
GTTACGGTATTAAAATTTTTCCCTTTGGCTTTGGCCTTTTTGTCTCTATATGTTGGTACA
TTCACATCTGTGAGATAATATCTCTGTCATTTGTCATGCAAAGTGTAAAAAGAAGAATGT
AACCAAAAGAAAATCTACTAAACTTGAGAACATAGTACAAAGAATGAAAACTGTTCCTGT
AGATGATGCTTCTTCCTATTAGTACAGGGTGCTTCTCCAAGCCTACGTAACAGGCATGAG
TTCCTTTCATTAAATACATTGCTCATTCTGTGTCCTGCCTAAGATGTCCACAAAGCTTGT
GAAGTCTAAAGTTAAGGGGCATGCGCTACAGGTTCTGACAACAGCATCTACCTTAGCTCT
CGAATCTCCACCCTGCTACTGCTACCCACTTCTACAGTGGATGACGGAACCTAACAGTTG
CCCTCTGCTGATTCTAACGTAGCTAGGTATGACCTCCATTTCCCAAGAAGACAACTTATA
CAGAATAAAATCATATCTGAAGGTAACAGAACATAAGACAGTCCAGAGGCCCATACATAC
CATCTGTGGCAGCATCTCCAGAGGCACAAGTCAGTTCTCTGAGAAAAGCTTGGGCTTCTC
ACTCTGTGCATGGTGAAGTAGAATGATGATGCGCAGGGTCCAGCTGATCTTATGAGAATT
AGACATGGCAGGCATTATCATGGAGACTCTTCCTCCAGGTTTCTGGATCATCTAAAGATG
TCGACAACACATTTAAATTTGGCTGTTCAGATCAGAGTAACAAATTTAATACTTTTTGTG
GGTGGTGGTGGTGGTGGGTTTTGGTTGGTTGGTTGGTTGTTTGGTTGGTTGGTTGGTTGG
TTTTTGTTGTTGCTGTTTTGTTTGTTTGTTCATTTGTTTGTTCTGTTTTTTTAAGACAGG
GATTCTCTCTGTAACATCACTGAATGTCCTAGAACTCATCTCATAGAACAGGCTGGCCTC
AAACTTACAGAGGTCCACATGCCTCTCCCTCCCGAGCTGGAATGAAGGGGTGCCCATGCC

CAGGCATTTAACACATCTTTAAAGAGAGTTTTGAGTTACTTTTATTGTTTATTTAGGCAT
TCCTATCTGTTCCTCTTGTCACATTTAATCCAGTTTCAACTACCACCTTCAAAATATAAT
TTATCATTGTACTGGCTGGTTTTGTGTCAACTTGACACAAGCTAGAGTTATCACAGAGAA
AGGAGCTTCAGTTGAGGAATGCCTCCATGAGATCCATCTGTAAGGCATTTTCTCAACTAG
AGATCAAGGGGGAAGGGCCCCTTGTGGGTGGTGCCATCCCTGGGCTAGGAGTCCTGGGTT
TTATAAGAAAGCAAGCTGAGCAAGCCAGGGAAGCAAGCCAGTAAAGAACATCCCTCCATA
GCCTCTGCATCAGCTCCTGCTTTCTGATTTGCTTGAGTTCCAGTCTTGACTTCCTTGGAG
ATGAACAGCAGTATGGATGTGTAAGCTGAATAAACCCTTTCCTCCCCAACTTGCCTCTTG
GTCATGATGTTTG

Enam_FIMO

motif_id	motif_alt_id	sequence_name	start	stop	strand	score	p-value	q-value	matched_sequence
1	HTTCTRGAAVNTTCKYM	5	5325	5341	-	12.4242	1.55E-05	0.363	TTTCTGGAAGAATATCC
1	HTTCTRGAAVNTTCKYM	5	6003	6019	+	12.4121	1.68E-05	0.363	TTTCTAGGAGCTTTGCA
1	HTTCTRGAAVNTTCKYM	5	8584	8600	-	11.8788	2.44E-05	0.363	CATATAGAAAGTTCTTA
1	HTTCTRGAAVNTTCKYM	5	4631	4647	+	8.1697	6.15E-05	0.574	ATCCTGGAACTCTCATC
1	HTTCTRGAAVNTTCKYM	5	17250	17266	-	8.14545	7.36E-05	0.574	CTTTTGGAAGCTCCTTT
2	DGAANDDDCN	5	8569	8578	-	9.44242	7.72E-05	1	GGAATTGGCC
2	DGAANDDDCN	5	13852	13861	+	9.44242	7.72E-05	1	GGAAGGAGCA
1	HTTCTRGAAVNTTCKYM	5	5761	5777	+	7.89091	8.98E-05	0.574	TTTCTGACACTTTCTCT
1	HTTCTRGAAVNTTCKYM	5	10322	10338	+	7.89091	8.98E-05	0.574	CTTCTAAAAGTTCCTAC
# FIMO (Find Individual Motif Occurrences): Version 5.5.6 compiled on Aug 4 2024 at 17:26:56									
# The format of this file is described at https://meme-suite.org/meme/doc/fimo-output-format.html#tsv_results.									
# fimo --oc . --verbosity 1 --bgfile --nrdb-- --thresh 1.0E-4 motifs.meme Enam.txt									

>7 dna:chromosome chromosome:GRCm39:7:43528328:43537375:1
CACACACACACACACACACACACACACACACACACACACACACACAAAGAGGGGTGGGAGAAAG
AAAGATCTCAATTTCTTCTGGACCAAGCACTCTGGGTAAAGAATCTTCCAGTCCTACTGT
GTACAAACCCTGAAGGTAGATTCAGAAGCAAAGGAGTGCTCCCCAGTTAAGTAAATAATA
GTAACCTATTGCTATGTCTGTTACTGTGACTCTGCCTATCAGTCTCCTCTCTCCCTGTCT
GCCTCGGTGCTTGTGGTCCTATCTCTAACTCTACGTGCACCTGTTTAACCCCTCCATAGT
GAATGAATATCACCAGCAAAAACAAGACACAGGAATCTGTCCTCAAAAGACTCCCAGCTT
AGGGGAAAACAGATGTTAGGAGAATTCACACACAAACAGAAACATGCATATGTTTTTCCA
TGGTCTCTGAGAGATGTACGGTGTAAAGCTCCTACCCAGAATCCTCTAGTGAGGGATGAG
TGTGAGGTTGTCATGTGTCCCTCCAGGTATGCGCTTCTGCTCTTCCCATCTGCAGATCTC
CACGTCTCCATCTCTCTGAACCTTCACGTCCTTCTGTGGTCTGGCATCCCCCAGTTCTCT
TGGTTCACATTTGTCCCTTCTTGTCTCTGAAGTGGATTCCCATCTTAGTGCAAATAACCC
CAATATGTGAGACCAGAAATGTTCCCAGTGCCTACATCCATAACTCTAGGTCACCTCCAC
CTGACCCAGTGGGAAGACAGAAAAGCACCACCCACCTGTTTGCTCTTTGTCTTAGCTGGC
CTCCCTGTCATCAGTACTGGCCTCATCAATCACAGTGTGTACCTATGAGTCCTGATAGAC
CCAGTATGCCCATTTGAATCCCAGACTTTCCCACCAAACCCCAGTATCTCCCTCCTGAAC
TCCAGCATCTCCCACCAGACCCCAGCATCCCCCTCCTGGACCCCAGCATCTCCCTCCTGG
ACCCCAGCATCTCCCTCCTGGACCTCAGCATCCCCCTCCTGGACCCCAGCATCTCCCTCC
TGGACCTCAGCATCTCCCTCCTGGACCTCAGCATCTCCCTCCTGGACTCCAGCATCTCCC
TCCTAGACTCCAGTATCTCCCTCCTGGACCTTAGCATCTCCCACCAGACCCCAGCATCTC
CCTCCTGGACTTCAGCATCTCCCTCCTGGACCCCAGTATCCCTTTCCTGGACCTCAGCAT
CTCTCACCAGACCCCAGTATCCCCCCTCCTGGACCTCAGCATCTCCCACCAGACCCCAGC
ATCTCCCTCCTGGACCCCAGCATCTCCCACTTGAATCTCAGTGCTTTCCCAGTCATGGTC
CAGCCTTCAATCCCTACCTCTGTCTCTCCTCACCAAGAGCAATTTCACAAAGTAAGCAAG
CCATGTCAATTCCTCTACCACTGAACCTTGATATCTAAGCCATTTCTGAAGGCTGCCTGT
CTGCCTTGCTTCCCATGGGAATGTCATCCACGGACAAATGCCAAGACGTCTTCTGATGCA
ACCTCACTTGTCTGGTTCCCACTCTTCCTCATAGTCATCTGACTCGGACACTACCAACTA
CCATTTATCTCTTGATTCCTCTGTCTTCTTGATTGTACATCTCTCCCCCTGATCACCGGG
ACCGGAGTCCCAGGAACATGGAGAGATCACTCTTCCTAGTTCCTGACTTGATTTAGAATT
CCAAGCATGAGAGCTGCCAATTGCTGTTGACTAAGGGGTGCCTCCATCACACCCTCGTTC
CTAGGAGCCTCTGCACTAGGACATCTGCAGAGGGAGGATCTGTGTGCAGGGTTCTCTGAA
TTCAGGCTGAGCATGGAGTGATGCTTACCCAGATCCTATGCATGGGGAAAAGGAGATGGA
GAGGTGGGTCACAGATAAGATTTCCAAGGAAGTACGAGATGCAAGCTGGATTTGGTGCTG
GGGCAGTTTTCAGAGGACGAACAGAAGGACACAGAGGGAGACTACGGGAGTGAGGTAGGG
GGAATTTCATTTAGTGAGTAAAATGAGCCTGAGAGAAGGAAAGGATGAATGAAAGAACAT
GAGGGGGGGCAAGGGAGGGGGAAGAATAAAGGCCAGAGAGGCTGGGGTCTGGACACTGTT
CCCTCCCTGTGCCCCAGACTCCACCTGACTTGGAGGCAGCCTGAAGGCTTTGAGCTGGAG
CCTGCAGCATCTGACGTATGGAATGTGTCCCCTGGGGGGCAGGCCAGCCCCAGCTGAGGA
AGCTTATAAGCCCCATAGGGGCCCCTGTGGGTAGCAAACCAGCTCAGCTTAGCTCAGCTC
AGCCGCAGAATTCGGAGCCCAGAAGCAGCAGGTGAGACACACACACACATTCTCTCTCTC
TCTCTCTTGCTCTCACACTCTCTGTTTAGAAGCATCCCTGCATTCCTCTCTGAACCTCTG
GCTCTGATCTCTGCCTCTCAGACTGCCTCCAACCAGATAGGTCTCTTGAATGCCCTGTCA
GGGTCTTCTATCTGTCCTAAGTGAAGCCCTGTCGGGGTCTTTCATCTGTCCTAAGTGAAG
ATCTATGCCTCCATTTCTCCACACCCCCACCCATCCCTAGATAGATGCTTCTCCCTCCTC
CTCCTCCTTCTCTTCCTCCTCCCTTTTACTTCTTCCCCCTTCTCTCTGTCTCTTTCTATC
CGGTCACATCCCACAAATGAAAAAGGCAAGGGAGAGAGATCATGGGGAGGCAGGGAGTAA
GACTACCGTCCAGATTCATTGTTGTCTTCATTTCCAGGAGCCAACATGATGGTCACTGCA
CGAACCCCCTGGGGCTGGTTCCTGGGGTGCCTCATCCTTGAGGTCACAGGTATCTGAGTG
TGTGTGTCATGGTGTGTGCCCGAGTGAACAGTGTCAGCCAACAGCAGGACTCAGTGTCCT
ACTGTTTCTGACTCTCTGATGTGGCAGTATGATGTGACTGTGTGTGGTCATGGCTGTAAA
GGGTGACAGGTCCTGAGAAGGAGAGACAGTCTTAGTGGTACCACTAAGGGGAAGACGGGA
TCTGTGCCCAGTTCCTGAGTGACTAGTTGATTCTTTCCGTTAAACTCCCTATGTACAACA
GGCTAGTCTCAAACTTTCAGAAGTCAGCCTGCCTCTGCCCCTGAGTGCTAGAGTTGATAC
CTGACCCACTTATTTATTTTTTAAGAGTCTTACTTAGTCCAGGCTGGCCGTGAATTCACT

AAGCAGCCAAGAACATCCTTAGATTTCCAATCCTTCTGCCTCTGCCTAAGTGCTGGGGTT
ATAGGTATGTACCACTATACCCAGTTTACGTGGTGCTGTAGTGCTGGGATCCAAACCCAA
GACTTTGTGCATGCTAAGCCAGCACTCCCTCCCAGCCCCCTCTGGGATAAAGATGTTGCC
TGGGTGAATGGGCTGTGGCTGGGTGTGCCTGAGGGGAGGTGCATGGAAGCACAGCCGTGC
CACAAAAGCTGAACAGCTCCTGAAGTCGTGACTGTGTGTGAGGGAGAAATGGTTCCTGGG
TCCTGAAGATCAGCCTGAGAATCCAAGCTGACCCGTCTTCCTGACAAATGACCCTACATC
CAGTGTGCTGATGATCCTGGCTCCCAGACTAAGGAGGCTCCTGGCTTTAACTCCTCTCAA
GGGACCTAACTCCACCCCTCTGAGCCCTGACCCGGAGCCCTCTCAGTCCTGACTCCACCC
CTCTGAACCCTGACTCCACCCTGAAGTTCTCAGACCACTCCAGTCACCAGTTGCTTGGTG
ACTGAAGACTAGTCCCATGTCCTATATCCTGAATCACCATCTAGTCCTGACTCTGCCAGC
CCCTTCACCGTAATTCTCCTCTCTCTGGCCCTGGCCTTTATGAACTCTGAAGCTTCACCC
ATACTCCTGTTCTCAAACTCGGTCCCTAAGAACCAGCCTCTTTGCCTGTCCCCAGACATT
CCCACATCCCTGAAGTACGACCTTGTCCCCAGATCTGCCCAGCTTATGTGGTGAACCCTA
AGCCTTCTCAGGAGGCAGATACCAAGGTCTCTACATTGACACCCTGCTATGATGAGCCCT
GCCTGCAACCAGTCCAGACTCTTGCCTCAACCACTCAGCTTTAAAGCAGTTTCCTTCCCC
TTTTCCATGACACCTCCCCTGAGACCTGGGAAGCCCCAGCCACATTTCTGCTCTGAAACT
CTCCCTGGTCCAGCGCCCTCATTCTGTCCCCAGCCCTGGCTCCACCCCTGAAAGCCTGGA
CAAGCCCCTGCACTAAATCTTACCTCGAGAGACAGCGAGGCTGTGTCCTGAACCTGTCTC
TAGCAATGACCCAGACCCTAAAACCTAATTCCATCCCCAAACTCTTAACCAAGTCCTTAA
CTTTGATATCCTACCCTTAGGAGCCCTAATCCTACCTCTTGAACTGGTTCCCTACCATGA
CCTCCCCCTGAGGCTCTAACCACTCCCCTTCACTCATACCCTGTGGGAAAGAACTCTAAT
CCAGGATTCATAGCTTGTTTCCAGTCTCGACCCTACCTCCTCCCCACCCCACCCCCGCAA
CAACACCCACCCACCCACTGCAACCTCTGATTACGTCCCTTCACTCAAACATCACCCCCA
TAGCTCTGAGCTCTCATCTGGACCCTGCTCCCATCCCAGAAGCCTTGAAACGCCCCTGCT
CCACACCCTCCCAATCCTGACTCCATCCCTAGGAGCCTCCGCCTCAAGTGTCAGCAGCCG
GATCATACAAGGCCAGGACTGCTCCCCACACTCGCAACCCTGGCAGGCGGCACTGTTCTC
AGAAGACGGTTTTTTCTGCTCGGGAGTCTTGGTGCATCCGCAGTGGGTGCTGTCAGCAGC
ACATTGCTTACAGGAGTGAGTCGGGGTGGTAGGGCAGGATGCTGAGGGGAGGGGTTTCAA
TATCACACGCGGCCTGCAGAGGGCGCGCAAGTGGCCACAGTGTTGAGGGGAACCCAAACC
TAAGGGACAGGGCAGTCTAGGGGAAGGGAAAGGCGAAGCAGGAGATCGCTGTTGCTTTAG
GGTGGAGGATGAGTTGGGAGATATAGGTGCCACTGTATACTCACCTTCTGGGTGATCCTC
AGGTCCTACATCGTGGGACTGGGCCTGCATAACCTGAAAGGCTCCCAAGAGCCTGGCAGC
CGGATGTTAGAGGCCCACCTCTCCATCCAGCACCCCAACTTCAACGATCCTTCTTTTGCA
AACGATCTCATGCTCATCAAACTGAACGAGTCAGTGATAGAGTCTAACACTATCAGGAGC
ATCCCTGTGGCTACCCAATGCCCCACTCCTGGAGATACCTGCCTAGTCTCTGGTTGGGGT
CAACTAAAGAATGGTAAGCTGCTGGGAACAGGGTTGCCCTCTCTGAGAAGTTCCCCACAC
AGCCAAAATGGACTGACCCTCCATTGTGTGCCCCAGGGAAACTGCCCAGCCTCCTGCAGT
GTGTGAATCTCTCAGTGGCGTCAGAGGAGACTTGCCGGCTGCTGTATGACCCTGTGTACC
ACCTCAGTATGTTCTGCGCTGGAGGAGGACAGGACCAAAAGGACTCCTGCAATGTGAGAC
TCGGGGAGGGTGGAACAGAGACGAGGCAGGGAGGTGCAGAAGGTACACCCAAGGCACACT
GAGGTAGACTGTGAGACATGGGGGAGCCACCGCTTTAATCTCAGCAGGGTAGGCAGAGTC
AGGTGATGTCTGTGAGTTCAAGACCAGCCAGATATACACAGTGAGACCCTTTCTCAAAAA
GAGGAGGAGATGGAGGAAGAGAATGGGGTAGGGGAGGAAAGAGGATCAAGGAGGCAGAGA
CCCAGAAGCCAGGCATAGTGGTTCAAACCTATAATCGTAGCCATTCAGTCCCTCCCACAG
TGAGGAGATAGGGCGTGGCTCAGGGGTAGAGCACCTCAATAAAGGCACTGGGTTTTGCTT
CCAGTACTACAAACAAACAAACAAACAAACAAACAAACACCACTAAATAGATCTCGGAAG
AAACAGAAATAGCCATTCTGAGAAAAAGTAAAAACGAAGGAAACACAGACACACACAGAA
CACAAAGACACAGTGTAGGCAGAAAAGCCCTGAAGGAGACAGACAGATAGACAAGGCAAG
TAACAAGCCCCAGGAAAGCAGCCAGTTGTGTGGACACAGGGCTCTGGCAAGGGTATGCGG
CTGGTTTCCTTCTGTCTCTGTCTACAATGCTTACTTACGGGGATGCTCTGAGACTTTCTG
CTCATGCCCTCCTGGATTCTGACTCTCTCTCCCTCACCCTCCCCTTCCCCCCTCACCCTC
TCCCTCTCTCTCCCACCTCCGTTTCACTTTCCCCCTCCCCCTCTCCTCCTCTTCCATTCC
CCTCCCTTCCTCCCCCCTTTTCTCTCTCAGGGGGACTCTGGGGGTCCCATAGTGTGCAAC
AGATCCCTACAAGGGCTCGTGTCTATGGGACAAGGAAAGTGTGGGCAGCCTGGCATACCA

AGTGTCTACACCAATCTCTGCAAGTTCACTAACTGGATCCAGACCATCATTCAGACAAAC
TAGCTATGGACTGACTACAAGGCCCACATGAAGCCTGCTGAGGGAACCTGAGACTCAGGA
TTGCTCCTAGCCCTTCTCCCTCAGATCTACAAGGTCAGGCCCTAGCCTCCTCCCTCAGAC
TCACAAGTCCAGACTCCAATCTCCTCCCTCAGACCCACAAGTCCAGACTCCAATTTCCTC
TCTCAGATCCAAGGATTCAGACCACAATACCCTCTTTCAGATTCAGGGATCCATGCCACA
ACCTCCTCCCTGGAACACAGGAGTGGAACCCACCTGCATTTTCCTCTGTGTGCAGTGTCC
TCTAGTAGCCCAGTATTGACCCAGCCTTGCCTTTGTCACCCTTAGGCTTGTTTCCCCCCC
CAAACACCTTATGCCTGTCTCCTCAGGGATCCACAAATAAAAGACCTCTCAGTTCTGTTT
GAAGTATCCTCTTCGTCCCTTTCCACTCAAGGGATATACCAACACATGGCTTTTCCTCTT
GGCTTCCCATCTGTGCAATGAGAATGTAAACCAGGAGGCCCCTGGCTTTTGATGCAGCAG
CCTGTGTCAGTGATGCTAAGAGACACCCATTCCATGATGTCCTCTCATTTCCAAAAGGGG
GATTTCCAGCTTTACAGCTTCTGTTGTGATTGGTCTGCTTTTGGTTTTGTCTTTTGATTT
TAAGATCTACTTCTTACTTTTATTTCTGTGTATTTGTGTGTGTGTGTGTGTGTGTGTGTG
TGTGTGTGTGTATGTACAAGCACACACGCTTGTGTAAGTGCAGTGGCCATGGAGACCAGA
AAAGGGCAGCATATCCCCTGGAGCTGGAGTTACGGGGGCTGTAAGCCGCTGAACATGGTT
GCTGGGAATCCAATCTCCATTTTCTGCGGGAGCAGCCGGTGTTCTCAACCATGCAGCCAT
CTCTCAAGCCCATTTTTTGTCTTCTCACACTTTAAGAACGTTTCTCTGTACCTGTCTCAG
GACCCAGCTGCTATTGCTGGAGCTGATGGAAAAAAAAATCTAAGATGCCTGCTGTGAGAC
CCTGAGACAGAGACAGGACACACTCAGCACCCGTAATAGGAGGAATTGCTATTAAGCTCC
TCATTAATAAATTATACCAGTAAAGTGGTGCATATCACATATCTGGAGTACAAGCTGAGG
TAGGGAGAGGAAGAGTTCAAGGCCATTCCAAACAAACAAACCAGATAGGTAGGTAGGTAG
GTAGGTAGGTAGGTAGGTAGATAGATAGATAGATAGATAGATAGATGATTGATTTTTGAA
TGTGCCTACAAAGGAAGTATAAAACTTTCGGCCGCCTATGTATTTAGTCTTGTTGTGCTT
TATTTTGTTTTGCTTTGAAACAGAACCGTAGGTAGCCCAGGCCGGCCTGGGCTTCATTAT
GCAGCTAAGGATGAGCTTAACTTTCTGACCCTCCTGGCTGCACCTCCCAAGCGCTGGGAT
GACAGGTGTGCGCCACCACACCCGACTATCAAAAATGTTTATGTATCATTAACAGATTCT
CAGACAGATGAAATATGGAGTAAGTTACTAAAGGTAGCATCAACAAGTTCCTCGTCATTG
AAGAGTTTCACAGTGACTTGGAGAAAATGACAGAAAGACCAGCCGAGACAGAAATGAGTG
GGTGTGTGCAAATGATTCACACAGTGTGATCCGCACACCTGTGAAACAGTGCATCCTCTG
TCTGCAGAACACACAACTCCTCACATGCACACACCTACCAAGATTGCTGACACTGCAGAA
TGTAACCAGCTCAAGTGTCAATTATACTCAGAGCAGGAACTTTAGGGAGATTTTAAAATG
GCACACCAAGTTAGAAAACAATCTAGCAGATTCTTTTTCAAAGTGTGTGTGTGTGTGTGT
GTGTGCATGCATGTGTGACTATTGCTGTACGTGTACCACAACACATATGTGAAGATCAGA
AGACTGCCTTGAGTGCTGGTCCTTCCACCTTGAGGCAGAGTCTCTTGTTTGCTGCCATAG
TCAGCAGGCCAGCTTCTAGGAATTCTCCTGTCTCTACCTCCCATCTCACCATACAAGTGC
TGGGATTACAGTTGCAGACACTATTTTGTCTAGCTTTCATGTGGGTGCTGGGGACCATCA
ATGGTTGCACAGCAAGTGATTTACAGGGGGGGCATCTTTCCAGCCCTCTAGCAGATTCTT
GCACATGCATCTGCTTGATGGTTATCACTTTCACCTCCAGTTATTTACCGGAGATCTGTG
GGGAAAACTAAAAATAGAAGCCCTGCTTAGGAATGGGGAAGGCAACTAAGTAGAGTGCTT
ACCTTCCAGGCATGGAGCCCTGGGTCCCATCACCACACTGTATAAGCTGGGCGTGCTGGA
ATAAGCCCATAACTCCAGTACTTGGGAGGCAGAGGTAGCAGGATCAGGAGTTCAAGACAG
TCCTCAGTTACTTGCCTAGTTTAATGCCAGTCTGGGCTGTGTGAGACCCTGTTGTCCCTT
CCCCTTATCACACAAATGTTTTACTTAGAAATCAGGTTGGAAATAGCTCAAGTGTGTGCA
GTTAGTGAATGACATAGGGACCTGGGATGAAATGGATCATGCAGTGAG

Klk4_FIMO

motif_id	motif_alt_id	sequence_name	start	stop	strand	score	p-value	q-value	matched_sequence
1	HTTCTRGAAVNTTCKYM	7	8060	8076	-	8.44242	4.6E-05	0.49	TTTCTGTCATTTTCTCC
1	HTTCTRGAAVNTTCKYM	7	4575	4591	+	8	7.5E-05	0.49	CATCTGGACCCTGCTCC
2	DGAANDDDCN	7	3778	3787	-	9.44242	7.72E-05	0.687	TGAAGGGGCT
2	DGAANDDDCN	7	8933	8942	-	9.44242	7.72E-05	0.687	GGAAGGGACA
1	HTTCTRGAAVNTTCKYM	7	8625	8641	+	7.90303	8.13E-05	0.49	CCTCTAGCAGATTCTTG
**# FIMO (Find Individual Motif Occurrences): Version 5.5.6 compiled on Aug 4 2024 at 17:26:56**									
**# The format of this file is described at https://meme-suite.org/meme/doc/fimo-output-format.html#tsv_results.**									
**# fimo --oc . --verbosity 1 --bgfile --nrdb-- --thresh 1.0E-4 motifs.meme Klk4.txt**									
